# The Essential Transition Metals Transport Systems in *Mycobacterium tuberculosis*: A Scoping Review of Emerging Targets for Antitubercular Drug Discovery

**DOI:** 10.3390/molecules31213992

**Published:** 2026-11-05

**Authors:** Moisés Maia Neto, Laura Helena Schroeder Borges, Gustavo Torres Corrêa, Cristiane Cunha Frota, Raul Edison Luna Lazo, Roberto Pontarolo

**Affiliations:** 1 Pharmaceutical Sciences Postgraduate Program, Federal University of Paraná, Av. Lothário Meissner, 632, Curitiba 80210-170, PR, Brazil; moimaian@gmail.com (M.M.N.);; 2 Department of Pathology, Federal University of Ceará, Alexandre Baraúna St., 949, Rodolfo Teófilo, Fortaleza 60430-160, CE, Brazil; 3 Universidad Científica del Sur, Lima 15067, Peru; rlunal@cientifica.edu.pe; 4 Pharmaceutical Sciences Postgraduate Program, Department of Pharmacy, Federal University of Paraná, Av. Lothário Meissner, 632, Curitiba 80210-170, PR, Brazil

**Keywords:** tuberculosis, transition metals, membrane transporters, metal ion transporters, metal homeostasis

## Abstract

In 2023, tuberculosis (TB) once again became the leading cause of death from a single infectious agent, with 1.25 million fatalities. Current TB treatment relies on the combination of drugs with different targets of action, as monotherapies have proven less effective and promoted the rapid emergence of resistance. Transition metals play an essential role in the metabolism of *Mycobacterium tuberculosis* (Mtb), influencing its growth and virulence. Restriction or exposure to toxic concentrations of these metals represent host cell defense mechanisms against Mtb. This scoping review aimed to map the main protein targets involved in the transport of essential transition metals across the Mtb membrane and to assess their potential in the development of novel anti-tuberculosis agents. The review was conducted according to JBI Collaboration guidelines and reported following the PRISMA-ScR checklist. Searches were carried out in PubMed, Web of Science, and Embase for studies published up to March 2025. A total of 72 studies were included, with iron being the most investigated metal (*n* = 48). The mechanisms of iron influx and efflux in Mtb are relatively well characterized; however, significant knowledge gaps persist regarding the role of the ESX-3 secretion system and the translocation of siderophores across the outer membrane. For each of the 79 entries mapped, database annotation was recorded separately from the evidence demonstrated in the included studies; in 65 entries the annotated direction of transport was not demonstrated experimentally. Comparing candidates by the level of functional evidence obtained in *M. tuberculosis*, the evidence available from macrophage and animal infection models and the existence of alternative pathways indicate that ESX-3, IrtAB and the siderophore export platform are the most completely supported systems; no inhibitor of any of these systems is reported in the included studies.

## 1. Introduction

Tuberculosis (TB) remains the leading cause of death from a single infectious agent worldwide, with 1.25 million deaths reported in 2023 and 8.2 million people newly diagnosed; an estimated 400,000 people developed multidrug-resistant or rifampicin-resistant TB, of whom only 44% were diagnosed and treated, with a treatment success rate of 68% for this form of the disease [1].

Current treatment of TB infection is based on a combination of drugs with different targets of action (Supplementary Material, Table S1). The importance of using a multi-target combination approach lies in the fact that monotherapies have proven less effective and have led to the rapid emergence of resistant forms. Resistance is established primarily through mutations in these protein targets or via the action of efflux pumps that expel the drug [2]. First-line treatment for drug-sensitive forms involves the use of isoniazid, rifampicin, pyrazinamide, and ethambutol. Second-line alternatives include streptomycin, moxifloxacin, linezolid, cycloserine, and ethionamide, with the more recent incorporation of bedaquiline, delamanid, and pretomanid. The inclusion of pretomanid in BPaL (bedaquiline, pretomanid, and linezolid) and BPaLM (bedaquiline, pretomanid, linezolid, and moxifloxacin) regimens has enabled the shortening of treatment duration for multidrug-resistant tuberculosis (MDR-TB) [3].

The need for investment in new therapeutic tools for tuberculosis treatment is increasingly urgent and remains below the targets set by the UN high-level meeting on TB. In 2022, approximately USD 1 billion was invested in TB-related research, while the annual target is USD 5 billion by 2027. According to the WHO, as of August 2024, there were 29 drugs for tuberculosis treatment in Phase I, Phase II or Phase III trials, corresponding to 18 new chemical entities [1]. Metal transport as a therapeutic target remains underexplored. Among the inhibitors of the mycobacterial membrane protein large 3 (MmpL3), only SQ109 has advanced to Phase II clinical trials for tuberculosis; BM212 has not progressed beyond preclinical models, and rimonabant, an anti-obesity agent withdrawn from the market, has been explored only as a scaffold for antitubercular analogues. In all cases these compounds act by blocking trehalose monomycolate transport rather than through functions associated with heme group translocation [4,5,6,7].

When Mtb is phagocytosed by macrophages, it must withstand several strategies developed by the host cell to combat it within the phagolysosomal microenvironment. These strategies include the depletion of essential metal micronutrients required by the microorganism (Fe^2+^ and Mn^2+^) or the increase in the concentration of other agents that may induce toxicity (Zn^2+^ and Cu^2+^) [8]. Three groups of metal ions are considered in this review. Essential transition metals, iron, zinc, manganese, cobalt, nickel, and copper, act as enzymatic cofactors in the response to oxidative stress, the biosynthesis of proteins and cell walls, energy metabolism, signal transduction, and DNA replication; zinc and copper are required in trace concentrations, yet they are also employed by the host at toxic concentrations. Non-essential, toxic metals, specifically cadmium and lead, have no known physiological role in *M. tuberculosis* and appear here only as alternative substrates for transporters whose primary function relates to essential metals. Non-transition metal ions, such as Mg^2+^, are included only when they share a transport pathway with an essential metal, as is the case with the CorA channel for Mg^2+^/Co^2+^ [9]. Even small changes in the availability of these metals have been experimentally shown to significantly impact the growth, virulence, and viability of the microorganism [10]. Many of these experiments use Mycobacterium smegmatis (Msmeg), a non-pathogenic, fast-growing, and easily manipulated organism, widely employed as an experimental model due to its close biochemical and genetic similarity to Mtb [11,12].

Future drug discovery strategies targeting metal transport in Mtb appear promising. Research in this field typically begins in the preclinical phase with the screening of large compound libraries through in silico and in vitro methods. For anti-infective agents, it is first necessary to select a target that performs an essential function for the microorganism, often proteins involved in enzymatic activity, signaling, or transport. This selection is based on the impact on microbial virulence and viability resulting from the deletion of genes encoding these proteins, as well as on structural information that includes binding sites, key residues, and conformational changes.

This review will address studies on proteins associated with the transport of essential transition metals in *Mycobacterium tuberculosis* and *Mycobacterium smegmatis*, organizing experimental evidence related to their structure, function, localization, transport mechanisms and identification in specialized databases. By exploring these aspects, the aim is to highlight the potential of these proteins as promising targets and to encourage research focused on the discovery of new anti-tuberculosis agents.

## 2. Results

**Figure 1:**
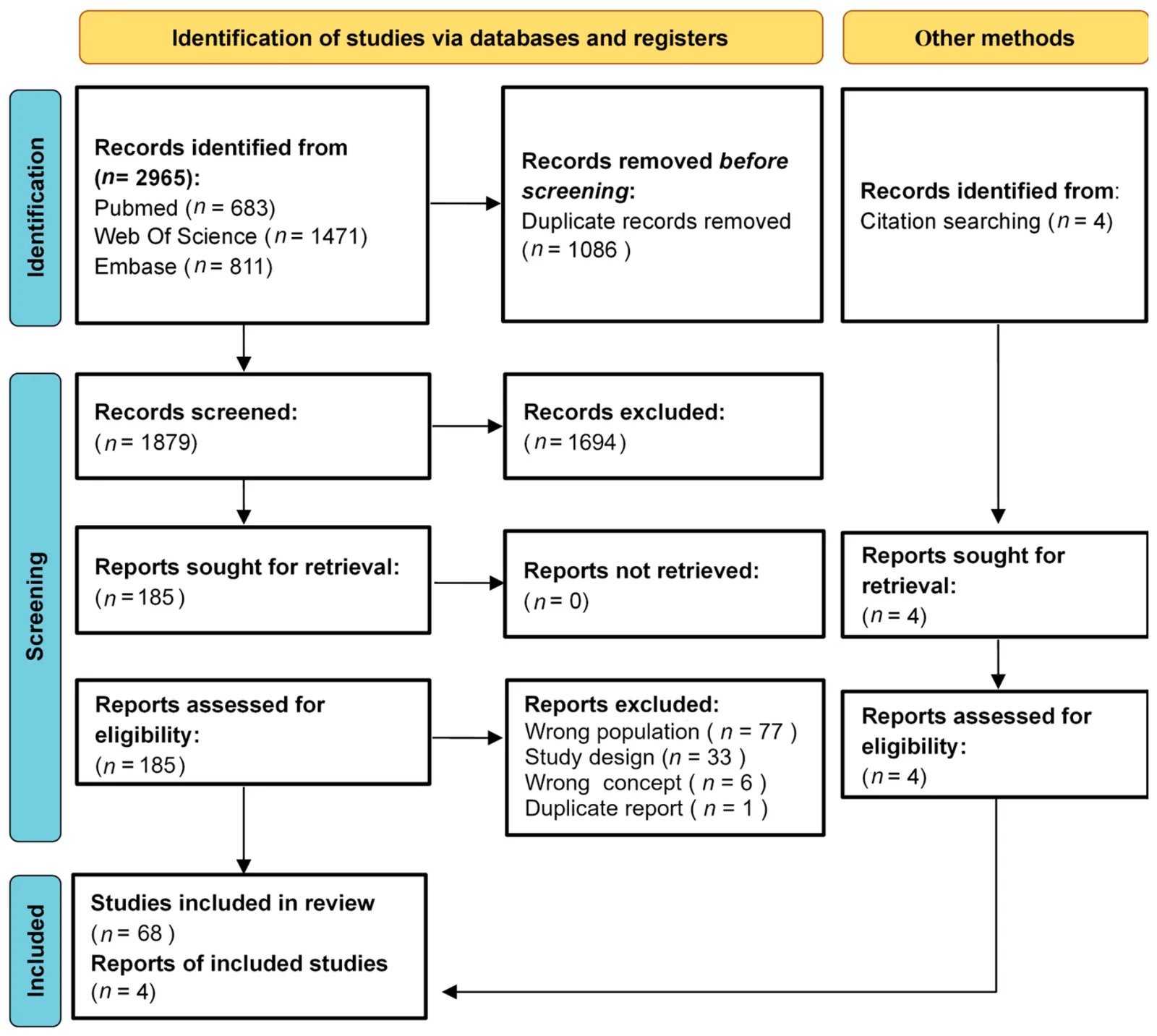
Flow diagram of the search, screening and eligibility processes of the studies included in this review. Adapted from PRISMA 2020 statement [14].

The database search initially retrieved 2965 records, which were processed using the SR-Accelerator tool [13], resulting in the removal of 1086 duplicates. Subsequently, 1879 records were screened by title and abstract, leaving 185 for full-text eligibility assessment. In the final stage, after four additional records identified through manual searches, 72 studies were included, as described in the flow diagram (Figure 1).

**Figure 2:**
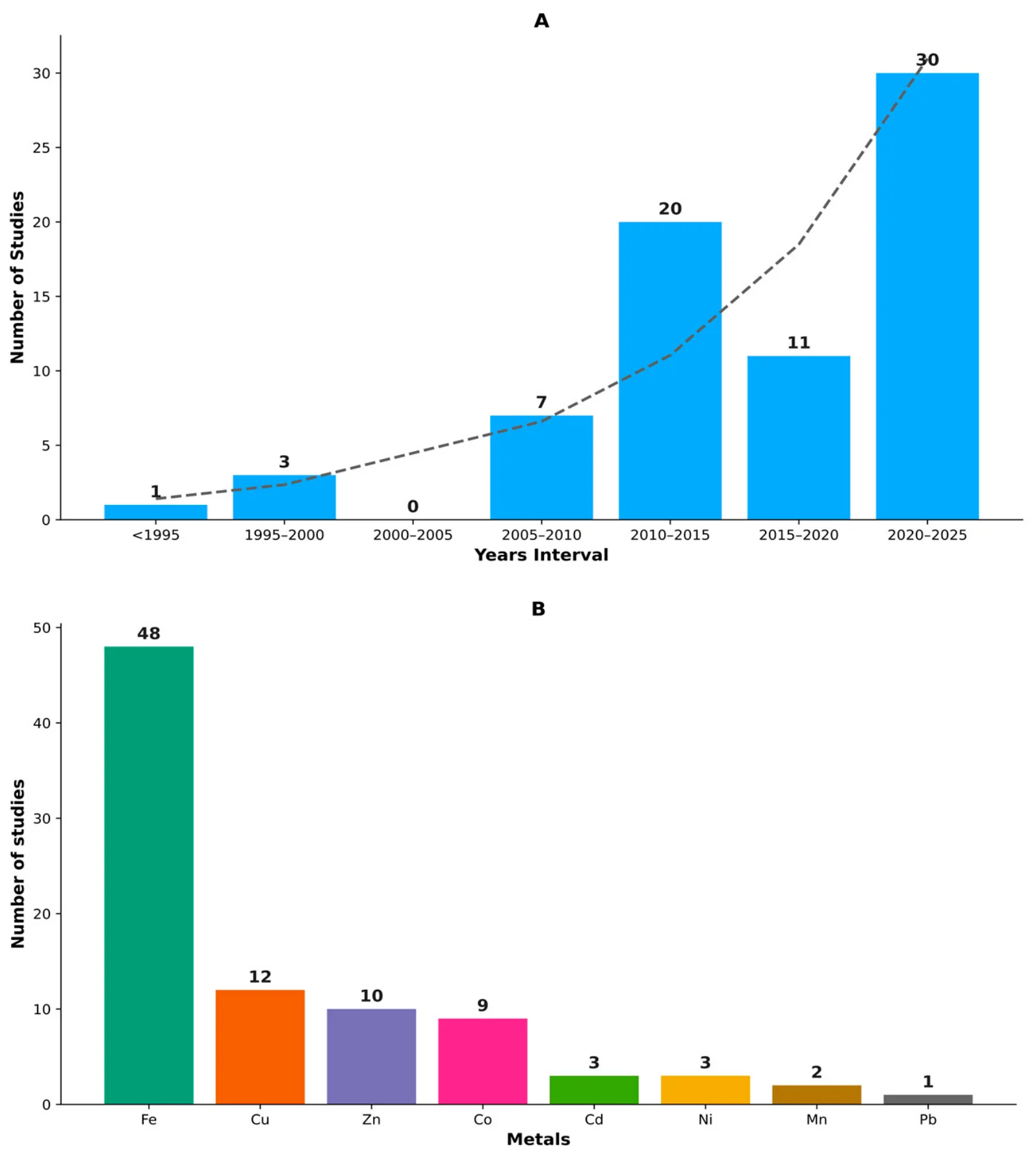
(**A**) Frequency of included studies in 5 year intervals with an exponential trend line. (**B**) Number of studies by transition metals.

The number of studies addressing the transport of transition metals across the Mtb membrane has increased significantly in recent years, with 41.67% of the records (*n* = 30) published in the current decade (Figure 2). Iron is the most frequently investigated metal (*n* = 48), followed by copper (*n* = 12), zinc (*n* = 10) and cobalt (*n* = 9) (Figure 2), reflecting the strong research focus on iron-related transport systems [10,15].

The complete genome sequencing of the virulent reference strain H37Rv of Mtb was first published in 1998, at which time approximately 40% of the proteins had their functions precisely annotated. Of the 3924 encoded proteins, about 360 were identified as membrane proteins, with 31 involved in cation transport [16]. Data deposited by the Sanger Institute in the NCBI-Datasets database (Genome assembly ASM19595v2) report a total of 4111 genes, with 4018 encoded proteins, 716 of which are grouped within the functional class cell wall and cell processes, where membrane transporters are located [17,18]. A total of 53 different proteins were identified from the included studies, all of which are already registered in databases such as UniProt [19], BioCyc [20] or RCSB PDB [21]. These records were compiled into a table cross-referencing literature data with structural, functional, and localization information from these databases, thereby establishing a mapping for each transported metal (available in Supplementary Material, Table S2).

### 2.1. Iron Transport

Iron is essential for the survival and virulence of Mtb, but its availability within the host is limited. To overcome this restriction, Mtb employs specialized uptake mechanisms, primarily through siderophore- and heme-mediated pathways. These pathways represent attractive therapeutic targets as they allow the limitation of bacterial access to this metal [15].

#### 2.1.1. Siderophore Pathway

**Figure 3:**
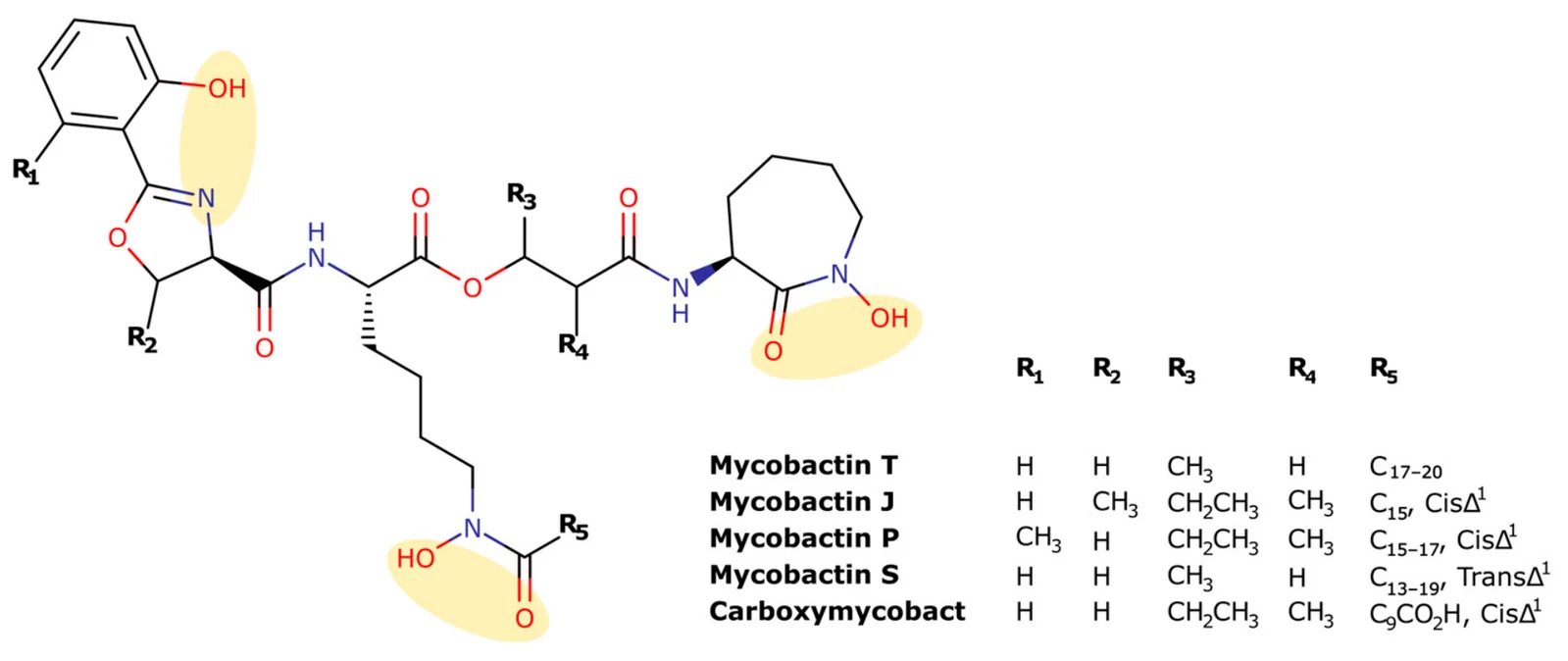
Structural representation of siderophores from the mycobactin and carboxymycobactin families. The groups involved in iron chelation are highlighted in yellow. Source: PubChem [22].

Siderophores are small molecules produced by Mtb that can chelate iron, thereby assisting in the sequestration of this metal from the host cell. Two families of siderophores have been identified in Mtb: mycobactin (Myc) and carboxymycobactin (cMyc) (Figure 3).

Myc contains a hydrophobic hydrocarbon chain at R5 and can associate with both the inner and outer membranes of Mtb. In contrast, cMyc has a hydrophilic carboxyl group at R5, which confers greater mobility within the phagolysosomal environment and the host cell cytoplasm [23,24]. The siderophore biosynthesis complex (SBC) is located on the cytoplasmic side of the inner membrane and is associated with the MmpL5/S5 complex. It is composed of two clusters: the first (*mbt-1*) consists of 10 proteins (MbtA to MbtJ), and the second (*mbt-2*) consists of 4 proteins (MbtK to MbtN). The expression of both clusters is negatively regulated by the transcription factor IdeR under conditions of high iron availability and positively regulated by HupB under low-iron conditions [10,15].

**Figure 4:**
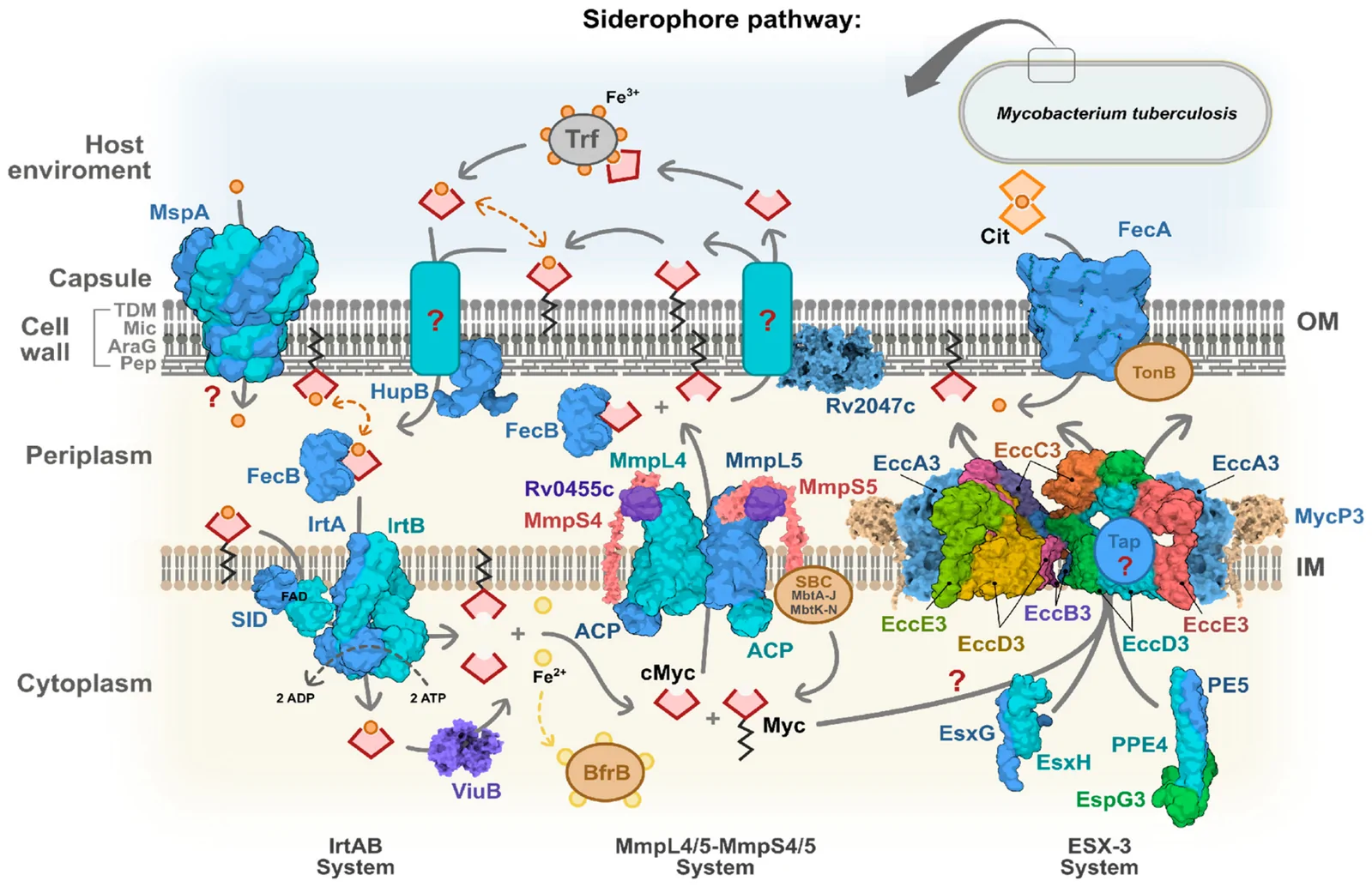
Siderophore-dependent iron transport. The image represents the outer (OM) and inner (IM) membranes, human transferrin (Trf), bacterioferrin B (BfrB), the siderophore interaction domain (SID), the siderophore biosynthesis complex (SBC), the siderophores mycobactin (Myc), carboxymycobactin (cMyc) and citrate (Cit), the proteins involved in transport (MmpS4/MmpS5 (mycobacterial membrane protein small 4 and 5), MmpL4/MmpL5, ACP, Rv0455c, FecB, Rv2047c, HupB, IrtAB, ViuB, EccA3-E3, EsxG/H, PPE4/PE5/EspG3, MycP3, FecA, TonB), in addition to the cell wall components with peptoglycan (Pep), arabinogalactan (AraG), Mycoloyl residue (Mic) and trehalose dimycolate (TDM). Question marks (?) indicate steps or components that, according to the included studies, have not yet been established: the outer membrane transporter(s) that mediate the secretion and reuptake of Myc and cMyc; the pathway by which siderophore-bound iron crosses the cell wall via a porin; the substrate translocated by the ESX-3 system; and the role of the Tap protein (Rv1258c). These gaps in knowledge are discussed in Section 2.1.1. Crystallographic representations of proteins were used; however, the scale ratio between them was not maintained. Created in BioRender.com and UCSF ChimeraX 1.3 [25].

The siderophore-mediated iron uptake pathway can be further divided into three main systems according to the principal transmembrane protein complex involved: IrtAB (iron-regulated transporter AB), MmpL4/MmpL5 (mycobacterial membrane protein large 4 and 5), and the ESX-3 type VII secretion system (Figure 4).

##### MmpL4/S4-MmpL5/S5 System

Proteins of the MmpL system belong to the resistance-nodulation-cell division (RND) superfamily of transporter proteins and are associated with other proteins such as acyl carrier protein (ACP), Rv2047c, and Rv0455c. They transport complex lipids and contribute to drug efflux and resistance [26,27]. The MmpL4 and MmpL5 transporters form functionally redundant complexes with their associated proteins, MmpS4 and MmpS5. Deletion of a single gene increased susceptibility to bedaquiline by only two-fold, whereas the simultaneous absence of both export systems rendered Mtb four to eight times more susceptible to bedaquiline and clofazimine, and two times more susceptible to rifabutin. The periplasmic protein Rv0455c, recently identified as essential for siderophore secretion, was also found to be required for resistance to these drugs, reinforcing the hypothesis that disruption of this complex may compromise Mtb viability. Furthermore, Alamar Blue microplate assays demonstrated that the MmpS5–MmpL5 system is more efficient in restoring the efflux of active compounds such as bedaquiline in double mutants, whereas the MmpS4–MmpL4 system requires accessory proteins to achieve the same function. A mutant lacking *rv0455c* exhibits an almost total absence of siderophores, as does the *mmpS4*/*mmpS5* double mutant. Given that blocking this platform both intoxicates the bacillus—due to the accumulation of iron-free siderophores—and impairs drug efflux, the authors propose it as a target for dual-function drugs, which would act independently while simultaneously enhancing the efficacy of existing agents [28].

In the context of iron homeostasis, this system mediates the transport of Myc and cMyc into the periplasm of Mtb. Mutant strains *ΔmmpS4*/*S5*, exhibited a drastic 100,000-fold reduction in viable cells within 14 days, even under iron-rich conditions. Studies in BALB/c mice with these same mutants revealed a 10,000-fold reduction in pulmonary bacterial load, with no signs of severe infection, indicating that these systems are essential for Mtb viability and virulence [29,30].

Molecular dynamics simulations of 50 ns using the Gromos43a1 force field for MmpL5 and MmpS5 revealed that MmpL5 has two main binding sites for Myc and cMyc, one cytoplasmic and the other periplasmic. The simulations also demonstrated that MmpS5 not only stabilizes interactions at the periplasmic site but also facilitates the release of Myc into the periplasm, promoting conformational changes in MmpL5 that enhance the efficiency of iron transport. Network analysis indicates interactions with proteins involved in siderophore biosynthesis and the type VII secretion system (ESX-3) further reinforcing its role in iron acquisition [31].

Similarly, mutants with deletions in *Rv2047c* and *Rv0455c* exhibit reduced siderophore secretion, increased sensitivity to exogenous siderophores, and a drastic reduction in bacterial load in the lungs and spleen of female mice [32,33]. Considering the localization of the Rv2047c protein in the peptidoglycan layer of the Mtb cell wall and its role in mycocerosate synthesis, it is believed that this protein may play a direct or indirect role in the export of Myc and cMyc across the outer membrane—an aspect regarded as a remaining gap in the evidence on siderophores [19,32,33].

Disruption of this system leads to intracellular accumulation of desferrated siderophores, a phenomenon known as “siderophore poisoning”, which severely compromises bacterial survival. The toxicity of the accumulated siderophores is comparable to that of antibiotics such as isoniazid and rifampicin [29,30].

##### IrtAB System

The heterodimeric iron-regulated transporter complex, composed of the IrtA and IrtB subunits, belongs to the ATP-binding cassette (ABC) transporter superfamily and functions as a siderophore importer in Mtb [34]. Cryo-EM structures revealed that IrtAB adopts an inward-facing conformation, with two nucleotide-binding domains (NBDs), while six transmembrane helices (TMHs) in each subunit form a collapsed cavity that opens laterally to the lipid bilayer but not to the extracellular space, forming an internal cavity suitable for transport ferric siderophores such as Fe^3+^-Myc and Fe^3+^-cMyc (Figure 4). Mutant studies confirmed that the IrtAB system is essential for iron uptake under low-iron conditions [35], as strains lacking IrtAB exhibit impaired growth and reduced replication in macrophages and in C57BL/6 mice infected via [35,36,37,38,39].

A protein–protein interaction (PPI) network analysis further identified IrtA as a central node connected to proteins involved in mycobactin and polyketide biosynthesis, both essential for iron uptake under low-iron conditions [40]. The results further indicated that IrtA is functionally associated with proteins such as MbtE and Hsp65, which participate in siderophore biosynthesis and heat shock response, respectively. Comparative analysis of PPI networks in virulent (H37Rv) and attenuated (H37Ra) strains revealed greater interconnectivity between iron transporters and stress-resistance proteins in the virulent strains, suggesting that these interactions are critical for Mtb pathogenicity [40].

Structural analysis of IrtAB revealed that mycobactin binds between the transmembrane helices TMH2 of IrtA and TMH5 of IrtB, near the periplasmic entry site. Mutants at residues Gln249 and Ala256 of IrtB, located within this cleft, demonstrated that alterations in these residues result in the loss of mycobactin import capacity, confirming the importance of this structure for efficient siderophore transport. The same study identified that TMHs of IrtA at the base of the cleft contain three histidine residues (His356, His402, and His407) that coordinate metal ions (Ni^2+^, Zn^2+^, or Co^2+^) during the transport process, potentially stabilizing the substrate-binding cavity and coupling ATP hydrolysis [41].

Inductively coupled plasma mass spectrometry (ICP-MS) experiments confirmed the selective binding of Zn^2+^ ions, which are required for transporter functionality. Substitution of the three histidine residues with alanine resulted in a complete loss of Myc import capacity, although ATP hydrolysis remained active, indicating that zinc coordination is crucial for the energetic coupling of transport [34]. These results suggest that the lateral cleft of IrtAB functions as a credit card mechanism, allowing the polar head of Myc to traverse the membrane while its lipophilic tail remains anchored in the bilayer, representing a unique adaptive strategy for iron acquisition in mycobacteria [34].

The N-terminal portion of IrtA, known as the siderophore interaction domain (SID), is located on the inner face of the membrane and contains a conserved flavin adenine dinucleotide (FAD)-binding motif. It plays an essential role in iron uptake by acting as a Fe^3+^ reductase, facilitating its release from the ferric–siderophore complex into the cytoplasm. SID mutants, in which the conserved residues Arg55, Gln234, and Arg241 were substituted with glutamate, lost the reductase activity of this domain. Reconstitution experiments with fluorimetric analysis in proteoliposomes demonstrated that IrtAB requires ATP hydrolysis to efficiently import Fe^3+^-Myc and Fe^3+^-cMyc [38]. The ATPase activity of IrtAB was stimulated more than 38-fold in the presence of Fe^3+^-Myc and 10-fold in the presence of Fe^3+^-cMyc, indicating a strong substrate preference for Myc [38]. The IrtA subunit exhibited higher ATPase activity than IrtB. In contrast, compounds such as ethidium and Hoechst 33342, which are typical substrates of multidrug ABC transporters, did not stimulate IrtAB ATPase, suggesting that the system is highly specific for siderophores. The reduction of Fe^3+^-Myc by the SID of *M. thermoresistibile* exhibited an apparent Michaelis constant of 185 μM, while surface plasmon resonance indicated a K_D greater than 200 μM for the binding of Fe^3+^-cMyc to the SID of *M. smegmatis*; both affinities are low and, in vivo, are compensated for by the high local concentration of membrane-bound Myc. Structural data indicate that SID is not essential for cMyc transport but is critical for Fe^3+^-Myc reduction, facilitating iron release into the cytoplasm [38].

The *Rv2895c* gene, a homolog of ViuB from Vibrio cholerae, previously proposed as essential for iron acquisition in Mtb, was shown to be dispensable for iron uptake under normal conditions. *Rv2895c* deletion mutants were able to grow as efficiently as the wild-type strain (H37Rv) in iron-limited media (~2 μM FeCl_3_), without showing significant alterations in iron uptake. Experiments with THP-1 cells differentiated into macrophages and infected with ST212 and H37Rv also revealed no differences in replication between the mutants and the wild type, indicating that *Rv2895c* is not required to overcome iron limitation in host cells [42].

FecB and FecB2 are considered substrate-binding proteins (SBPs) in Mtb. Both have resolved crystal structures and differ substantially in shape, charge, and interacting residues. Only FecB has the ability to bind both heme and cMyc, in either their Fe^3+^-bound or unbound forms [43]. Mutation of the Glu339 residue in FecB abolished binding to Fe^3+^-cMyc, indicating its importance for this specific interaction. FecB interacts with components of the siderophore export (MmpL4/5 and MmpS4/5) and import (IrtAB) systems, as well as with a component of the ESX-3 system (EccE3); these interactions were confirmed by co-immunoprecipitation, establishing its role in the siderophore-dependent pathway. The FecB protein has a deep, negatively charged binding pocket capable of accommodating bulky siderophores such as Fe^3+^-cMyc [43]. The IrtAB system plays a central role in siderophore-mediated iron uptake and contributes to the survival and virulence of Mtb, making it a potential therapeutic target.

##### ESX-3 System

The type VII secretion system ESX-3 is present in all Mycobacterium species and represents one of the five ESX systems found in Mtb. The transmembrane complex is composed of five subunits: EccA3, EccB3, EccC3, EccD3, and EccE3. It is essential for manganese, zinc, and iron homeostasis mediated by Myc. Expression of its locus is tightly regulated by the repressors IdeR (Iron-dependent Repressor), Zur (Zinc uptake repressor), and MntR (Manganese transport Regulator) [44]. Its expression is induced in response to iron deprivation, suggesting a direct role in iron acquisition.

Experiments with ESX-3–deficient mutants (*Δesx*-3 strain to *fxbA*^−^) demonstrated severe growth defects under iron-limiting conditions, even though the mutants are still capable of synthesizing Myc and cMyc [45]. Comparative growth analyses of the wild-type BCG strain and the BCG-tet-*esx-3* mutant strain of M. bovis, in the presence or absence of anhydrotetracycline, showed a greater than 10-fold reduction in growth for the mutant strain in J774 macrophages and SCID mouse models, indicating that impaired use of Myc compromises survival in iron-poor environments. Mutants with deletions in the eccC3ms, esxGHms, espG3ms, and eccD3ms genes exhibited severe growth defects under low-iron conditions, indicating that these components are critical for the export of EsxG and EsxH proteins, which are required for Myc utilization. The accessory component of ESX-3, MycP3, is necessary for EsxG and EsxH export but is not absolutely required for mycobactin-mediated iron acquisition, showing a more modest effect upon deletion of the mycP3ms gene. Supernatant peptide quantification in mutant and wild-type strains demonstrated that low levels of EsxG and EsxH export are sufficient to permit growth under iron-limiting conditions. Restoration of the ESX-3 system recovered growth capacity, confirming its critical role in siderophore-mediated iron acquisition [45,46].

Using a conditional *esx-3* mutant (TB79) regulated by anhydrotetracycline (ATc), Serafini et al. demonstrated that *esx-3* repression results in severely impaired growth in standard media (Middlebrook 7H9 and 7H10), even under high concentrations of iron and zinc. Microarray and RT-PCR analyses showed that *esx-3* repression led to the overexpression of genes normally induced during iron and zinc deprivation, including *mbtB*, *irtA*, and *rv1460*, as well as genes from the Zur (Zinc uptake regulator) regulon, such as *rv2059* and *rv2060*, indicating that the absence of ESX-3 triggers a cellular response typical of metal deficiency. Repression of ESX-3 drastically reduced the incorporation of Fe^3+^-cMyc, while streptonigrin sensitivity assays showed that *esx-3*–deficient cells have lower intracellular iron levels, confirming that the ESX-3 system is required for efficient iron import in Mtb. ESX-3 repression resulted in restored growth upon supplementation with 450 μM iron or 36 μM zinc, confirming its role in the acquisition of these essential elements. Complementation with supernatant from wild-type cultures also rescued the growth of the mutant, suggesting that ESX-3 is responsible for the secretion of soluble factors that facilitate iron and zinc uptake, possibly functioning as a surface-permeabilization system for these metals [47].

*Δesx-3* mutants revealed intracellular accumulation of cMyc and Myc in relative abundances ranging from ~80-fold to 600-fold, suggesting that the secretion of proteins such as EsxG, EsxH, PE5, and PPE4 is necessary for efficient siderophore export. This was confirmed by genetic complementation assays restoring the expression of these genes, which recovered the capacity for iron uptake and growth under iron-limited conditions [48]. In the same investigation, *ΔesxH* and *ΔesxG* strains were associated with attenuated Mtb virulence in BALB/c SCID mice, increasing the time to lethality from 22 days in the wild-type strain (H37Rv) to 65 days and 12 months, respectively, whereas *Δpe5–ppe4* and *ΔmbtB* mutants showed only modest prolongation to 27 days. These results suggest that EsxG and EsxH have additional functions independent of iron metabolism, contributing to Mtb virulence. EsxG and EsxH are required for the export of members of the PE and PPE protein families, including PE15 and PPE20, which are important for modulating host immune responses [48].

The crystallographic structure of the PE5–PPE4–EspG3 heterotrimer from the ESX-3 secretion system of Mtb revealed that EspG3 interacts exclusively with the N-terminal domain of PPE4, shielding a critical hydrophobic region at the top of the PE5–PPE4 dimer, which is required for efficient transport of these substrates. The PPE4–EspG3 interface is stabilized by a network of hydrophobic interactions, hydrogen bonds, and two salt bridges centered on Glu140 of PPE4. Substitution of residues Phe128 and Phe129 in the HH motif of PPE4 with charged amino acids disrupted the interaction with EspG3, confirming the importance of this interface for complex formation. In contrast, mutations in residues forming hydrogen bonds (Asn127, Asn132, and Glu140) were not sufficient to abolish the interaction, indicating that the hydrophobicity of the HH motif is the main determinant of PPE4–EspG3 interaction specificity [49].

The PE5–PPE4–EspG3 heterotrimers of ESX-3 adopt a markedly different structural orientation, with distinct interaction angles and deeper interfaces compared to PE–PPE–EspG5 complexes. This structural difference appears to be crucial for the specificity of ESX systems, allowing each EspG to recognize only its cognate substrates, without cross-recognition between the different secretion systems of Mtb [49].

The iron-regulated HupB protein, located in the cell wall of Mtb, is essential for siderophore-mediated uptake of cMyc and Myc. The wild-type (WT) strain of Mtb was able to efficiently import ^55^Fe bound to cMyc, whereas the *ΔhupB* mutant strain almost completely failed to do so, even under iron-deficient conditions. Complementation of the mutant strain with the *hupB* gene restored this capacity. Additional studies using liposomes containing purified HupB confirmed that the protein promotes the transfer of iron from cMyc to Myc. Assays with rabbit anti-HupB antibodies significantly inhibited iron uptake, corroborating the specific role of HupB as an Fe^3+^-cMyc receptor at the bacterial surface. Moreover, spectroscopic and surface plasmon resonance (SPR) studies revealed that HupB binds both ferric siderophores and free iron. Surface plasmon resonance yielded dissociation constants in the nanomolar range for Fe^3+^-Myc (K_D = 0.346 nM), free iron (1.29 nM), and Fe^3+^-cMyc (2.8 nM)—presented as single values, without replicates or error estimates. Given that HupB’s affinity for free iron is intermediate between that of the two siderophore complexes—a finding consistent with the previously described liposome assay—the authors proposed a sequential transfer of iron: from cMyc to HupB, and subsequently to Myc. Molecular docking models of HupB with point mutations indicated that residues in the C-terminal region of HupB are involved in binding to cMyc, whereas Myc primarily interacts with the N-terminal portion through hydrophobic interactions. The authors propose a model in which HupB acts as an essential mediator of iron transfer between the two siderophores, serving as the key link for efficient iron transport across the complex lipid barrier of the mycobacterial cell wall [50].

The multidrug efflux pump Tap, belonging to the major facilitator superfamily (MFS) of transporters, is directly involved in oxidative stress regulation and iron metabolism mediated by the ESX-3 secretion system in Mtb. Deletion of the *Rv1258c* gene encoding Tap resulted in a 30–50% reduction in the relative expression of ESX-3 system genes (*eccA3*, *eccC3*, *eccD3*, *eccE3*, and *mycP3*), except for *eccB3*, and caused extracellular iron accumulation with intracellular depletion, suggesting impaired iron uptake. Protein–protein interaction predictions using AlphaFold2 and yeast two-hybrid assays in the NMY51 strain with recombinant constructs pPR3-N-*Rv0286* (PPE4), pPR3-N-*Rv0290* (EccD3), and pPR3-N-*Rv0283* (EccB3) as targets demonstrated that Tap strongly interacts with PPE4 and EccD3, suggesting a regulatory role on the transmembrane channel for iron uptake. This represents a novel and still poorly understood role of the efflux pump in controlling iron metabolism via ESX-3, potentially influencing bacterial iron homeostasis directly. In parallel, the absence of Rv1258c led to an increased NAD^+^/NADH ratio, indicating induction of oxidative stress. Additionally, genes from the MmpL and MFS families, such as *mmpL6*, *mmpL11*, and *kgtP*, were downregulated in the absence of Rv1258c, revealing that this efflux pump broadly affects stress response systems and solute transport [51].

FecA is a TonB-dependent transporter (TBDT) involved in ferric siderophore uptake in Mtb. Shankar et al. demonstrated through molecular docking and 500 ns molecular dynamics simulations that Mtb FecA shares the conserved barrel-and-plug architecture typical of TBDTs, with ferric citrate initially binding to an external surface site before progressively migrating through the β-barrel–formed tunnel into the periplasmic space. RMSD and RMSF results revealed that ligand-bound FecA exhibits greater flexibility and conformational dynamics compared to the apo form, particularly in β-strands 14–17 and in extracellular loops L6 and L7. These changes facilitate ligand passage through initially narrow tunnel regions, suggesting an induced fit model as the mechanism of transport. FecA functions as a component of an iron uptake system in Mtb that remains poorly characterized, involving the coordinated action of FecA in the outer membrane, periplasmic substrate-binding proteins (SBPs), ABC transporters in the inner membrane, and the TonB–ExbB–ExbD complex for energy transduction [52]. Table 1 summarizes the 25 proteins associated with the siderophore pathway—10 related to influx and 15 to efflux—distributed across the outer membrane, periplasm, cell wall, and inner membrane, including their gene names, structural records (PDB/AlphaFold), and identifiers from UniProt and Mycobrowser.

#### 2.1.2. Heme Pathway

This iron acquisition pathway in Mtb is based on the direct utilization of free heme or hemoglobin (Hb) as a source of this metal. The main proteins involved in this pathway include the dipeptide permease components DppA–DppD, MmpL3, MmpL11, PPE36, PPE37, PPE62, PE22, Rv0203, the heme-degrading enzyme MhuD, and FecB2.

The PE22 and PPE36 proteins, located on the Mtb cell surface, and the transmembrane protein PPE62, located in the outer membrane, have been identified as essential for heme and Hb uptake in Mtb. These proteins facilitate heme transfer to DppA, which then delivers it to the DppBCD transporter for translocation across the inner membrane. This uptake pathway is essential for the use of heme as an iron source by Mtb, particularly in iron-poor environments such as macrophage phagolysosomes. DppA is a periplasmic substrate-binding protein (SBP), a lipoprotein, that binds heme and delivers it to the DppBCD permease; translocation across the inner membrane is carried out by the subunits of DppBCD, rather than by DppA itself. The critical heme-binding site involves residue Arg179. Mutation of this residue abolished DppA’s ability to bind heme, resulting in the inability of the Δdpp mutant to utilize this iron source. Albumin may provide an alternative route for heme uptake, independent of the Dpp and PPE systems, by binding heme directly and facilitating its internalization. Mtb can exploit multiple pathways to acquire iron from heme, adapting to different host microenvironments [58].

PPE37 is an integral membrane protein required for the acquisition of heme iron; it has not yet been determined whether it mediates the translocation itself or acts at an earlier stage in the pathway. Available evidence places its action after the initial capture of heme at the cell surface. In the vaccine strain M. bovis BCG, deletion of nine residues (ARAWHALAA) located at positions 31–39 of the N-terminal region may explain the poor growth of this strain in media containing heme as the sole iron source, even at high concentrations (up to 50 μM). These residues are present in Mtb, which exhibited robust growth even at very low heme concentrations (0.078 μM). Genetic complementation of BCG with *ppe37* from Mtb fully restored this growth capacity, reducing the effective heme concentration required for growth by more than 100-fold. Similarly, deletion of *ppe37* in Mtb generated a heme-deficient phenotype nearly identical to that observed in BCG, confirming the indispensable role of this protein in the uptake system. Furthermore, more than 60% of Mtb strains with complete genomes harbor mutated variants of *ppe37*, with deletions or mutations that compromise the protein’s function in heme uptake [59].

The MmpL3/MmpL11 system, belonging to the Resistance-Nodulation Cell Division (RND) superfamily and located in the inner membrane of Mtb, participates in heme transport into the cytoplasm, with heme previously captured and delivered by the periplasmic protein Rv0203, which acts as a hemophore. A double mutant, *MtbΔmbtBΔRv0203*, showed reduced growth of up to 83% in the presence of heme and 61% in the presence of hemoglobin compared with the *MtbΔmbtB* mutant. Rv0203 displayed a heme-binding affinity (Kd ≈ 5.4 μM) similar to that of bacterial hemophores. Deletion of the *mmpL11* gene also resulted in an 87% reduction in growth in 7H9 medium supplemented with heme, confirming its role in this transport pathway. Complementation with plasmids carrying the deleted genes restored both growth and heme uptake, indicating the specific functionality of these proteins. The crystallographic structure of Rv0203 reveals a unique fold and a heme-binding site dependent on residues Tyr59, His63, and His89. The extracellular domains (E1) of MmpL3 and MmpL11 also demonstrated heme-binding capacity, whereas the cytoplasmic domains did not. Analysis of heme association and dissociation rates showed that MmpL3-E1 has higher affinity than MmpL11-E1. After translocation into the cytoplasm, heme is degraded by the protein MhuD, releasing free Fe^2+^ [60,61,62].

The MhuP or FecB2 protein (Rv0265c) is another SBP, orphan of transmembrane (TMD) and nucleotide-binding (NBD) domains, responsible for heme uptake and delivery to the Dpp system for cytoplasmic import of heme in Mtb. Its crystallographic structure, determined in two apo forms, revealed a typical cluster A SBP arrangement, with two globular domains (NTD and CTD) connected by an α-helix. Spectroscopic analyses (differential absorption and fluorescence) confirmed that FecB2 binds specifically to heme, but not to hydroxamate-type siderophores such as ferrichrome, nor to the glycyl-glutamate peptide. The dissociation constant of the FecB2–heme complex (Kd ≈ 389 μM, determined by fluorescence quenching—a value consistent with the ≈300 μM previously obtained via SPR) is approximately 260 times higher than the value of ≈1.5 μM reported for the DppA–heme complex; this indicates a much weaker interaction and supports the hypothesis that FecB2 acts as a transient heme carrier, releasing the cofactor to DppA for subsequent translocation via the DppBCD system. Molecular docking and in silico mutagenesis analyses identified critical residues involved in heme coordination, including Tyr33, Trp52, Gln176, Arg192, and Arg215 [63].

Mutation of Tyr33 (Y33A) reduced heme binding energy by 38%, demonstrating its functional importance. Protein–protein docking studies indicated that FecB2 interacts with DppA at the heme-binding site, and that transfer likely occurs through the Ala143–Arg151 loop region of DppA [63]. FecB2 also interacts with FecB and with proteins of the Opp system (OppA and OppD). FecB2 contains a shallow, neutral binding pocket suitable for heme binding, suggesting a specific role in the heme-based iron acquisition pathway [43].

The type VII secretion system ESX-4 is essential for the efficient utilization of heme as an iron source in Mtb. Deletion of the *eccC4* gene, which encodes an ATPase essential for ESX-4 function, delayed the onset of exponential growth from 12 to 30 days in media containing free heme or heme–albumin, an effect reversed by complementation with the corresponding gene. Use of an intracellular heme biosensor showed that the *ΔeccC4* mutant accumulates less heme in the cytoplasm than the wild-type strain, suggesting that ESX-4 facilitates heme entry, likely through the export of auxiliary proteins [64].

Proteomic and genetic analyses have substantially refined our understanding of heme acquisition in *Mycobacterium tuberculosis*, revealing a coordinated surface-associated and secretory network. Loss of ESX-4 significantly reduces the export of Rv0125 and Rv1085c—both required for efficient heme uptake—indicating an indirect role for this secretion system in heme utilization [64]. Rv0125 (a putative serine protease) and Rv1085c (a putative hemolysin) likely facilitate heme release from hemoglobin, whereas PPE family proteins act at the bacterial surface in heme capture. Notably, PPE64 binds heme and forms high-conductance channels (~11 nS) in artificial lipid bilayers, exceeding that of canonical porins such as MspA (mycobacterial porin A), representing one of the first demonstrations of pore-forming activity within the PPE family [64]. Importantly, PPE64 export is ESX-4–independent, suggesting that multiple trafficking pathways contribute to the localization of heme-interacting proteins. Complementary TnSeq analysis identified 165 genes with distinct requirements for iron acquisition from heme, hemoglobin, mycobactin, and carboxymycobactin, demonstrating that ESX-3—previously associated primarily with siderophore-mediated uptake—is also essential for heme utilization, thereby challenging the notion that siderophore and heme pathways function independently [32].

**Figure 5:**
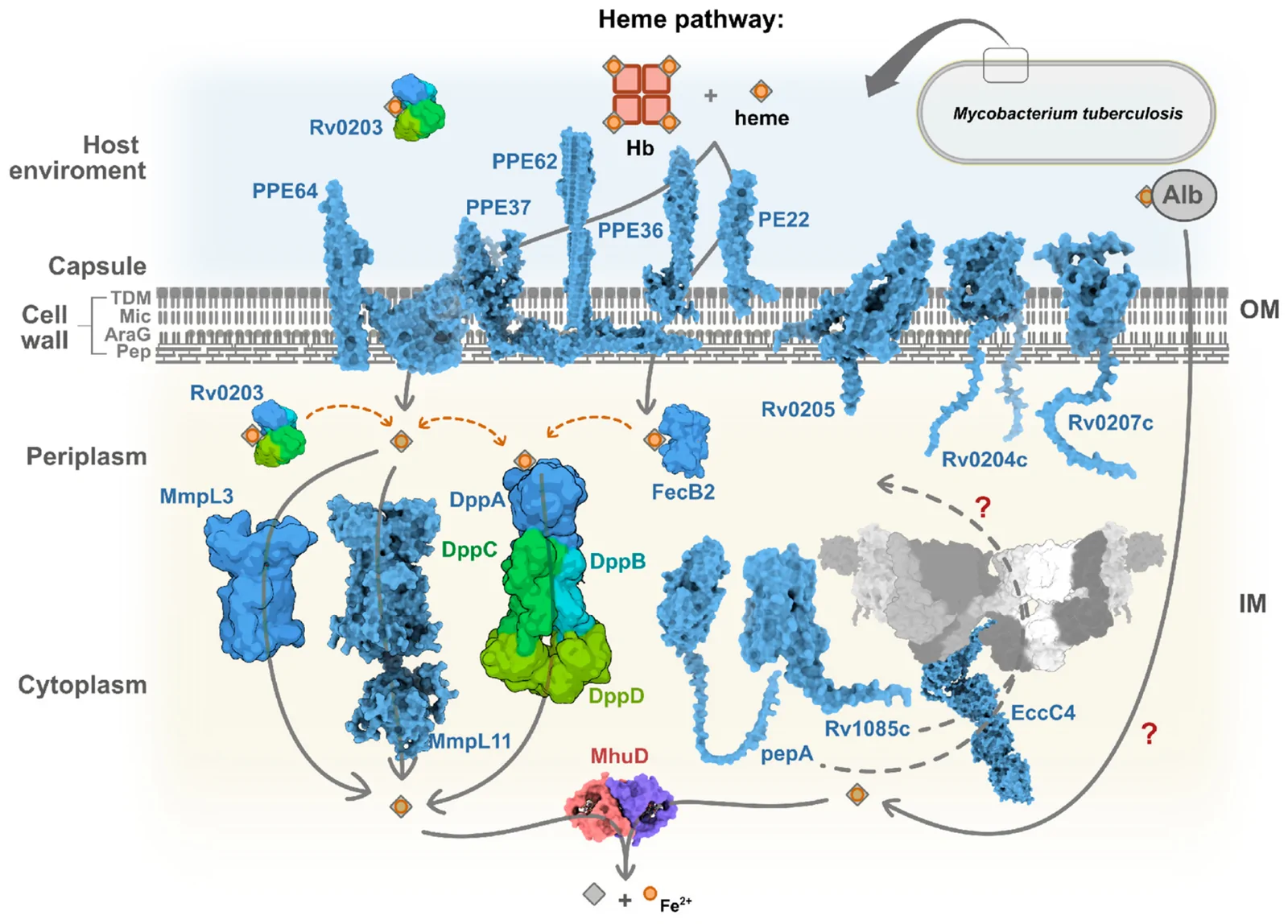
Heme-dependent iron transport. The image depicts the outer membrane (OM) and inner membrane (IM), cell wall components including peptidoglycan (Pep), arabinogalactan (AraG), mycoloyl residue (Mic), and trehalose dimycolate (TDM), as well as human hemoglobin (Hb) and the heme porphyrin group. Shown in their crystallographic forms are inner membrane proteins (MmpL3, MmpL11, DppA–D, Rv1085c, EccC4), periplasmic proteins (FecB2 and Rv0203), and outer membrane proteins (PE22, PPE36, PPE62, PPE37, PPE64, Rv0205, Rv0204c, and Rv0207c). The ESX-4 protein complex is represented in gray, with only the component referenced in this study (EccC4) highlighted. Question marks (?) indicate steps or components that, according to the included studies, are not yet established: the mechanism by which the ESX-4 system contributes to heme utilization—which appears to be indirect and mediated by the export of Rv0125 and Rv1085c—and the albumin-dependent pathway for heme delivery to the cytoplasm. These gaps in the evidence are discussed in Section 2.1.2. Protein representations are not drawn to scale. Created in BioRender.com and UCSF ChimeraX 1.3 [25].

The integration of heme acquisition with broader iron homeostasis networks. The MmpL5/MmpS5 efflux system is critical for siderophore secretion, while Rv2047c is required for growth in the presence of mycobactin, reinforcing the interconnectedness of siderophore and heme pathways [32]. Transcriptional regulators such as Zur and SmtB are also necessary for efficient heme utilization, indicating that adaptation to iron-rich niches involves coordinated regulatory control. Genes associated with porphyrin biosynthesis, lipid metabolism, and ESX-3–mediated secretion further contribute to heme-dependent growth [32]. These findings indicate that *M. tuberculosis* employs multiple mechanisms to utilize heme as an iron source under host-imposed restriction, functioning alongside siderophore systems [58,61]. Since this pathway is structurally and mechanistically distinct from human heme metabolism, it represents for a potential therapeutic target, including strategies that harness the heme uptake machinery as a vector for targeted drug delivery [4]. The heme-dependent transport pathway is presented in Figure 5, and Table 2 summarizes the 20 proteins associated with the heme pathway, all of which are involved in heme acquisition. No heme exporters were identified in our search, consistent with Mitra et al., who state that potential heme efflux in Mtb “has not been experimentally examined yet” [58]. Internalized heme is catabolized by MhuD, releasing Fe^2+^, and the efflux of this iron has been attributed to the P-type ATPases CtpD and CtpJ, which are listed in Table 3.

#### 2.1.3. Alternative Iron Acquisition Pathways in Mycobacteria

In addition to the classical siderophore-mediated pathway, *M. tuberculosis* employs several alternative mechanisms for iron acquisition. The glycolytic enzyme GAPDH (*Rv1436*), located on the bacterial surface, it binds to holotransferrin with a dissociation constant of 160 ± 24 nM, as determined by microscale thermophoresis, and contributes to receptor-mediated iron uptake—independently of the ESX-3 system—under intracellular and iron-limiting conditions [65]. The *mtaA* gene product, CtpC (*Rv3270*), a P-type ATPase homologous to copper and cadmium transporters, is transcriptionally co-regulated with the adjacent Irp10 (*Rv3269*) in response to iron limitation, suggesting that they may form a two-component metal transport system [66,67,68]. The Mramp transporter, a mycobacterial homolog of the natural resistance-associated macrophage protein (Nramp), functions as a pH-dependent divalent cation transporter (Fe^2+^, Zn^2+^, Mn^2+^, Cu^2+^) with maximum activity at pH 5.5–6.5, compatible with the intraphagosomal environment, and is expressed in both axenic culture and THP-1 macrophages, playing a fundamental role in the intracellular acquisition of metals during infection [69]. Finally, the P1B4-type ATPases, MtCtpD and MtCtpJ, contribute to Fe^2+^ and Co^2+^ homeostasis: MtCtpD binds to Fe^2+^ with a K_D of 120 ± 40 nM—an affinity approximately 20 times higher than that of MtCtpJ (2410 ± 270 nM)—and is essential for iron detoxification and virulence; conversely, it is suggested that MtCtpJ controls steady-state Fe^2+^ levels [70].

A distinct iron-uptake pathway operates in *M. smegmatis* and has no equivalent in *M. tuberculosis.* Four iron-regulated envelope proteins are induced under iron-limiting conditions in this species; the 29 kDa protein, for instance, is absent in spheroplasts [71]. This protein binds to ferri-exochelin, as demonstrated through direct binding, affinity chromatography, and ion-exchange separation [72]; its molecular identity, however, remains unknown. Another iron-regulated 21 kDa protein—designated IrpA (MSMEG_3761, annotated as a subunit of a Clp protease)—was subsequently characterized as a ferri-exochelin receptor and is absent in *M. tuberculosis* [73]. From a functional perspective, deletion of the *fxuABC* exochelin-uptake operon reduced biofilm biomass to 9.5% of the control level in the standard biofilm medium, which requires a 2 mM iron supplement, without affecting planktonic growth [74]. Table 3 summarizes the nine proteins associated with alternative iron-acquisition pathways, comprising four influx components and three efflux components, in addition to IrpA, for which the direction of transport remains undetermined; the entry for MspA is derived from *M. smegmatis*.

**Table 3:** Proteins involved in iron transport through other pathways. GAPDH: Glyceraldehyde-3-phosphate dehydrogenase, Msp: Mycobacterial small porin, Mramp: Mycobacterial resistance-associated macrophage protein.

Protein Name	Databank Information					Functional Evidence	Reference
	Gene	PDB/Alpha Fold (AF)	Uniprot ID	Mycobrowser (Locus)	Location/Function		
GAPDH	gap	8BR4/AF-P9WN83-F1	P9WN83	*Rv1436*	Outer membrane Influx	Binding/delivery (surface receptor for holotransferrin)	[65]
Manganese-exporting P-type ATPase, Metal-transporting ATPase Mta72 (MtaA) or Cation-transporting P-type ATPase CtpC	*ctpC* or MtaA	AF-P9WPT5-F1	P9WPT5	*Rv3270*	Inner membrane Efflux	Not determined for iron (identified as an iron-regulated ATPase; see Table 4 for the Zn^2+^/Mn^2+^ evidence)	[66]
10 kDa iron-regulated protein Irp10, the same locus later designated PacL1 (see Table 4)	irp10/pacL1	AF-L7N4H1-F1	L7N4H1	*Rv3269*	Cell wall and Inner membrane	Not determined (iron-regulated; co-transcribed with ctpC/mtaA)	[66]
Divalent metal cation transporter MntH (Mramp)	*mntH*	AF-P9WIZ5-F1	P9WIZ5	*Rv0924c*	Inner membrane Influx	Transport (influx) (^65^Zn^2+^/^55^Fe^2+^ uptake in Xenopus oocytes; see Table 4 for the Mn^2+^ evidence)	[69]
Putative ferri-exochelin receptor (29 kDa)	Not Found	Not Found	Not Found	Not Found	Not Found	Binding/delivery (binding demonstrated by three methods)	[71,72]
Iron-regulated protein IrpA (putative Clp protease subunit)	*irpA*	AF-A0QYR7-F1	A0QYR7	*Msmeg_3761*	Cell wall Influx	Binding/delivery (ferri-exochelin receptor; identified by MALDI-TOF-MS/MS)	[73]
Porin MspA	*mspA*	1UUN/2V9U/AF-A0QR29-F1	A0QR29	*Msmeg_0965*	Outer membrane Influx	Transport (influx) for Fe^3+^-citrate (iron uptake reduced ~40% in the triple porin mutant ML16; *M. smegmatis*). For Cu and other divalent metals see Table 4	[75]
Probable cobalt/nickel-exporting P-type ATPase	*ctpD*	AF-P9WPT3-F1	P9WPT3	*Rv1469*	Inner membrane Efflux	Transport inferred (efflux) (metal-stimulated ATPase and metal content in mutants; flux not measured; *M. tuberculosis*—see Table 4 for the *M. smegmatis* evidence)	[70]
Probable cation-transporting P-type ATPase J	*ctpJ*	AF-P9WPT7-F1	P9WPT7	*Rv3743c*	Inner membrane Efflux	Transport inferred (efflux) (metal-stimulated ATPase and metal content in mutants; flux not measured; *M. tuberculosis*—see Table 4 for the *M. smegmatis* evidence)	[70]

Note: The columns under “Databank Information” reflect annotations obtained from UniProt, BioCyc, Mycobrowser, and RCSB PDB. The “Functional Evidence” column summarizes the evidence reported in the studies included in this review: Transport (direct transmembrane transport demonstrated), Transport inferred (metal accumulation or depletion in mutants, or metal-stimulated ATPase activity, consistent with a transport function but without measurement of transmembrane flux), Binding/delivery (substrate binding or delivery demonstrated; transport not demonstrated), Accessory (required for the pathway, but not a transporter itself), and Not determined (association reported without demonstration of binding or transport). Any discrepancies between the two sets are discussed in the corresponding section of the text. CtpC (*Rv3270*), *Rv3269*, CtpD (*Rv1469*), CtpJ (*Rv3743c*), MntH (*Rv0924c*) and MspA also appear in Table 4. Entries are listed per pathway and the functional evidence is classified per metal, so the same protein may carry different classifications in the two tables; the 79 entries therefore correspond to 53 distinct proteins.

### 2.2. Transport of Other Essential Transition Metals

During human macrophage infection by Mtb, there is a rapid and sustained increase in the levels of certain transition metals within host cells, particularly in the phagosomal vacuoles that harbor the bacillus [76]. This accumulation is part of a host immune strategy to intoxicate the pathogen. Twelve candidate P-type ATPases have been identified in the Mtb genome, of which seven (CtpA, CtpB, CtpC, CtpD, CtpG, CtpJ, and CtpV) constitute the transition metals transport group, responsible for the active transport of ions such as Cu^+^, Cu^2+^, Zn^2+^, Co^2+^, Ni^2+^, Ag^+^, Hg^2+^, Cd^2+^, and Pb^2+^. In general, CtpA, CtpB, and CtpV show specificity for Cu^+^, CtpC functions as a Zn^2+^ pump, CtpG is associated with Cd^2+^, while CtpD and CtpJ are linked to Co^2+^ and Ni^2+^ transport. CtpA, CtpC, CtpG, CtpV, and CtpF are induced during macrophage phagocytosis, highlighting their relevance for bacterial survival and persistence. Conserved structural motifs identified in these transporters reinforce ion specificity and suggest mechanisms of resistance to metal toxicity during infection. Expression of genes such as *ctpF* and *ctpC* is induced by different antimicrobial compounds and stress conditions, including hypoxia and the presence of reactive nitrogen species, indicating an important role for these ATPases in intrinsic drug resistance and in Mtb adaptation to the hostile host environment [77].

Heterologous expression of the *ctpA* gene in Msmeg showed that recombinant cells exhibited increased tolerance to toxic levels of Cu^2+^ (up to 4 mM) compared with the control, while resistance to other metals such as Co^2+^, Mn^2+^, Ni^2+^, and Zn^2+^ was not observed [78]. Enzymatic activity assays with plasma membrane vesicles extracted from these cells revealed that ATPase activity was specifically stimulated by the presence of Cu^+^, but not by Cu^2+^ or other metals, suggesting that Cu^2+^ is reduced to Cu^+^ in the intracellular environment and that CtpA functions in Cu^+^ efflux. Bioinformatics and structural modeling studies showed that residues responsible for Cu^+^ coordination and transport in known ATPases are conserved in CtpA, reinforcing its functional role as a copper efflux pump [78].

More recent studies, however, demonstrated a weaker role for CtpA in direct copper detoxification and a greater importance in tolerance to redox stress [79]. Gene expression assays showed that *ctpA* is strongly induced at low copper concentrations as well as under oxidative (H_2_O_2_) and nitrosative (sodium nitroprusside) stress, but not at high copper concentrations. *ctpA*-deficient mutants did not show significant differences in tolerance to high copper doses nor did they accumulate more intracellular copper compared with the wild-type strain, suggesting that CtpA is not involved in classical copper efflux for detoxification [79]. On the other hand, these mutants displayed impaired growth and greater sensitivity to oxidative and nitrosative stress. The study further demonstrated that the absence of CtpA reduces the oxidase activity of organic substrates dependent on multicopper oxidase (MmcO), a periplasmic protein that requires copper as a cofactor. CtpA is important for providing copper to MmcO, facilitating its metallation and activity, which is critical for the neutralization of reactive oxygen and nitrogen species during infection. The increased expression of *mmcO* in *ctpA* mutants under redox stress reinforces the role of CtpA in maintaining cuproenzyme activity and, consequently, in the antioxidant defense of Mtb [79].

The CtpB protein specifically transports copper ions in the reduced form (Cu^+^) and is essential for optimal Mtb growth under copper-limiting conditions. Topological and motif analyses confirmed the presence of typical domains of copper-transporting ATPases, including residues essential for cation coordination. Deletion of the *ctpB* gene impaired growth only under copper-limiting conditions, but not in copper-rich medium, suggesting a specific role in metal acquisition when scarce, rather than in detoxification. Gene expression was strongly induced under conditions mimicking the intraphagosomal environment, such as hypoxia and redox stress, but not by copper excess, and was regulated by the sigma factor SigC. However, there is an apparent contradiction between experimental results under toxic copper concentrations. Shey-Njila et al. [80] reported that heterologous overexpression of ctpB impaired growth in medium containing 0.1 mM copper, interpreting this effect as the result of an influx activity, which would increase toxicity under excess conditions. In contrast, León-Torres et al. [81] observed that ATPase activity was stimulated and heterologous expression of *ctpB* conferred greater tolerance and growth compared with the control at concentrations of 2–2.5 mM, suggesting a role in Cu efflux.

In the study by Shey-Njila et al. [80], using a murine model (DBA/2 mice), the ctpB mutant strain exhibited hypervirulence, leading to more rapid mortality compared with the wild-type strain, despite no apparent differences in bacterial load or pulmonary pathology. This finding suggests that during infection, copper uptake deficiency allows the mutant to partially evade host-mediated intoxication, thereby favoring its survival and replication. Phylogenetically, CtpB is highly conserved among pathogenic intracellular mycobacteria, underscoring its evolutionary importance in circumventing host nutritional immunity mechanisms [81].

The *zitA* gene, which encodes the zinc tolerance protein A (ZitA) of the cation diffusion facilitator (CDF) family, is the main determinant of zinc resistance in Msmeg. A zinc-sensitive mutant, isolated by transposon mutagenesis, showed an insertion in the *zitA* gene and exhibited tenfold greater sensitivity to the metal compared with the wild-type strain, without alterations in resistance to other metals. Expression of *zitA* is specifically induced by zinc, as demonstrated by *lacZ* fusion assays, and its deletion drastically reduced bacterial growth in the presence of zinc. Complementation of the mutant with the *zitA* gene restored resistance, confirming its role in zinc homeostasis. Comparative analysis showed that Msmeg carries only one functional copy of CDF, whereas other environmental and opportunistic mycobacteria possess two or more, and obligate pathogenic species such as *M. tuberculosis* harbor only one. Cross-complementation assays with orthologs from other mycobacteria revealed varying degrees of resistance restoration, depending on the species of origin of the gene [82].

Following phagocytosis, Mtb strongly induces expression of the efflux pump CtpC, as well as CtpG and CtpV. Transcriptomic assays, RT-qPCR, and microscopy analyses confirmed that both bacterial genes and host defense genes (such as metallothioneins and the ZnT1 transporter) are activated in this context. Zinc accumulates around and within the bacteria, with most retained at the surface or in the cell wall, but it can also accumulate in the cytoplasm under stress conditions. The relevance of this mechanism was demonstrated by experiments with Mtb mutants deficient in the *ctpC* gene, which exhibited intracellular zinc accumulation, structural damage, and impaired growth within human macrophages. Restoration of the gene rescued zinc resistance and bacterial viability. Although no significant attenuation of the mutant was observed in infected BALB/c mice, likely due to compensation by other ATPases, the importance of CtpC and other transition metals efflux pumps for Mtb survival in zinc-rich hostile environments is clear [76].

Mtb resistance to zinc intoxication also depends on an efflux platform formed by the interaction of CtpC with the chaperone-like protein PacL1 (P-type ATPase-associated chaperone-like protein 1). PacL1, encoded in the same operon as CtpC, contains a transmembrane domain, a cytoplasmic region with glutamine/alanine repeats, and a C-terminal metal-binding motif (MBM). Deletion of *pacL1* or of the *pacL1*–*ctpC* operon renders the bacteria highly sensitive to excess zinc, while genetic complementation restores resistance. Co-expression of PacL1 and CtpC, but not either protein alone, is sufficient to protect against intracellular zinc accumulation in mutant strains. PacL1 functions as a zinc chaperone, with its metal-binding motif being essential only under high zinc concentrations, whereas the cytoplasmic region (particularly the Glu/Ala repeats) is crucial for the stability and localization of CtpC in the membrane. Fluorescence microscopy revealed that PacL1 and CtpC cluster into dynamic foci in the plasma membrane, forming high-molecular-weight complexes independent of flotillins, suggesting the existence of specific efflux microdomains. Functional assays showed that the absence or mutation of the PacL1 Glu/Ala repeats reduces CtpC membrane levels and increases zinc sensitivity [67].

CtpC is also a high-affinity, low-turnover manganese (Mn^2+^) transporter. Biochemical analyses with the purified protein showed that CtpC ATPase activity is strongly stimulated by Mn^2+^, in contrast to other metals. *ctpC*-deficient mutants exhibited cytosolic accumulation of Mn^2+^, increased sensitivity to oxidative stress, and a significant reduction in the activity of secreted superoxide dismutase (SodA). Genetic complementation restored these phenotypes, confirming the direct role of CtpC in Mn^2+^ homeostasis and in the functional assembly of secreted metalloproteins. Infection assays in C57BL/6 mice demonstrated that the absence of *ctpC* compromises bacillary persistence during the adaptive phase of the immune response, leading to attenuated virulence. Interestingly, although *ctpC* is induced by Zn^2+^ excess and mutants display hypersensitivity to this metal, the protein cannot restore zinc tolerance in *E. coli* strains deficient in Zn^2+^-ATPase, further reinforcing its specificity for manganese. CtpC plays a dual role in protecting against oxidative stress and in the metallation of extracellular proteins, particularly SodA, and is essential for Mtb virulence in the hostile environments imposed by the host [83].

CtpC is also known for its role in multidrug resistance in Mtb. Overexpression of *ctpC* in *E. coli* and Msmeg resulted in a significant increase in resistance to several antibiotics, including fluoroquinolones (norfloxacin, ofloxacin, sparfloxacin), β-lactams (ampicillin, oxacillin), and isoniazid, as well as reduced intracellular accumulation of these compounds, as evidenced by antibiotic and dye accumulation assays. This efflux activity was confirmed by the use of reserpine, an efflux pump inhibitor, which restored bacterial susceptibility to the drugs. The resistance effect was even more pronounced in the presence of zinc, which particularly increased tolerance to levofloxacin, suggesting a synergistic role between metal transport and antibiotic efflux. *ctpC* expression enhanced biofilm formation in both *E. coli* and Msmeg, a phenomenon also reversed by the efflux inhibitor. Molecular docking studies suggested that CtpC may directly interact with different antibiotics at specific sites within the cytoplasmic domain, facilitating their efflux together with metal cations [68].

The proteins CtpD and CtpJ are fundamental for cobalt (Co^2+^) and nickel (Ni^2+^) transport. Msmeg mutants deficient in *ctpD* exhibited hypersensitivity and intracellular accumulation of these metals, but not of zinc or copper, indicating physiological specificity. Interestingly, *ΔctpD* mutants in Mtb did not accumulate Co^2+^, suggesting that the primary role of CtpD in this microorganism is not cytoplasmic homeostasis, but rather the metallation of redox-active proteins such as thioredoxins—TrxA (*Rv1470*) and TrxB (*Rv1471*)—whose ability to bind Co^2+^ and Ni^2+^ was confirmed in vitro [84].

Gene expression analyses showed that *ctpD* is induced by Co^2+^ and by superoxide stress (paraquat), as well as by other redox stressors such as nitroprusside and cyanide, and is co-expressed with oxidative defense genes, reinforcing its role in adaptation to oxidative stress within the hostile phagosomal environment. CtpJ, whose expression is also induced by Co^2+^, displays a high Co^2+^ transport rate and regulates cytoplasmic cobalt levels, as evidenced by cobalt accumulation in *ΔctpJ* Mtb mutants. The ATPase is activated by Co^2+^ and Ni^2+^, and to a lesser extent by Zn^2+^, and contains a transmembrane binding site capable of coordinating a single metal ion. Experiments with double mutants (*ΔctpD ΔzitA*) demonstrated that CtpD does not contribute to zinc efflux, reinforcing its preferential role in cobalt and nickel homeostasis. Functionally, experiments in female C57BL/6J mice showed that only *ctpD* deletion significantly reduced bacterial fitness in the lungs, whereas ctpJ deletion did not affect virulence [84]. Thus, although both transporters are Co^2+^/Ni^2+^ ATPases, CtpJ is essential for controlling cobalt levels, while CtpD is crucial for the assembly of metalloproteins involved in redox stress tolerance and, consequently, for Mtb virulence [85].

Porins, specifically MspA and its paralogs (MspB and MspC), are involved in the transport of various metals, including Cu^2+^, Fe^3+^, Ni^2+^, Co^2+^, Mn^2+^, Zn^2+^, Pb^2+^, and Cd^2+^, across the outer membrane of mycobacteria. Patch-clamp techniques in Msmeg nanopores demonstrated that variants of the MspA protein containing histidine, aspartate, or cysteine residues at strategic positions within the channel lead to individual metal ion binding events. The results showed that histidine residues interact strongly with ions such as Zn^2+^, Co^2+^, Ni^2+^, and Pb^2+^, while cysteine residues exhibited higher affinity for metals such as Cd^2+^ and Pb^2+^. It was further demonstrated that the duration of binding events can be precisely modulated by the pH of the medium, increasing the stability of the metal–amino acid complex under alkaline conditions [86].

These porins are also essential for copper and iron uptake in Msmeg, particularly at low concentrations. Porin-deficient mutants exhibited impaired growth in copper-trace medium, suggesting a defect in ion uptake [87]. These mutants also showed increased resistance to high copper concentrations, indicating that porins act as important channels for the entry of this potentially toxic metal. Heterologous expression of the MspA porin in Mtb increased copper sensitivity. At 25 μM copper, the MspA-expressing strain failed to grow after 24 days, and even at lower concentrations (2.5 μM), growth was reduced by 40%, underscoring the role of the outer membrane as a protective barrier against copper excess. In alamarBlue assays, the polyamine spermine, a known porin inhibitor, rendered Msmeg less sensitive to ampicillin (fixed at 80 μg/mL) in a concentration-dependent manner; at concentrations of 25 and 50 μM, it also reduced the bacterium’s susceptibility to copper. In Mtb mc^2^6230, combinations of copper (ranging from trace amounts to 20 μM) and spermine (0–300 μM) yielded the most pronounced protective effect at copper concentrations of 15 and 17.5 μM, while showing no effect at concentrations of 12.5 μM or lower—a finding that suggests copper enters the cell through channels in the outer membrane [87].

Using a triple porin-deficient mutant (ML16), iron uptake from ferric citrate was reduced by approximately 40% compared with the wild-type strain. This was accompanied by increased expression of IdeR-regulated genes, even under conditions with 150 μM iron, suggesting that the cell perceived the environment as iron-deficient. Reintroduction of the MspA porin restored iron uptake levels and gene expression to the wild-type pattern [75].

Multiple ZnuABC-type (zinc uptake ABC) transporters in Msmeg have shown that both the classical ZnuABC transporter and an actinobacteria-specific variant (ZnuABC2) are regulated by the global zinc sensor Zur and are essential for metal homeostasis. In Msmeg, deletion of a single transporter (znuABC or znuABC2) did not cause significant growth defects but increased the expression of zinc-deficiency marker genes, indicating cytosolic limitation. Simultaneous deletion of both resulted in severe growth defects, which were rescued only by complementation with homologous transporters or by zinc supplementation. Uptake assays with FluoZin-3 acetoxymethyl (FluoZin-3AM) confirmed that both transporters are capable of efficiently importing zinc. Structural and phylogenetic analyses revealed that ZnuABC2 represents a new class of transporters restricted to actinobacteria, mainly genera such as Nocardia, Streptomyces, and fast-growing mycobacteria [88].

ArfA (Rv0899) has been characterized as an outer membrane protein composed of a monomer with two separate domains (B and C) and an N-terminal transmembrane domain responsible for its anchoring to the membrane. ArfA does not function as an oligomeric channel-forming porin but rather as a membrane-anchored protein, with its B and C domains extending outward from the lipid envelope [89]. The domains of this protein do not interact with peptidoglycan or capsule polysaccharides, suggesting that its primary function is not related to structural binding to the cell wall. ArfA binds to Zn^2+^ with a dissociation constant of 0.1–0.2 mM, as determined by NMR chemical shift perturbation and isothermal titration calorimetry. The authors note that this affinity is too low for the direct import of Zn^2+^ across the outer membrane; instead, they propose that ArfA may assist in recruiting Zn^2+^ to the cell surface as part of a hypothetical acquisition complex. This role has not been experimentally demonstrated; therefore, in this context, ArfA is not considered a zinc transporter [89].

A copper-resistant Msmeg strain (mc^2^155-Cu) exhibited fivefold greater resistance to copper sulfate and twofold greater resistance to isoniazid (INH) compared with the parental strain. Quantitative proteome analysis revealed that, among the 345 differentially expressed proteins, the P-type copper-transporting ATPase was the only transporter upregulated, while all other ABC transporters were downregulated. Increased expression of this ATPase was confirmed at both the protein and mRNA levels, suggesting its role in copper resistance. The P-type copper-translocating ATPase of Msmeg shows high sequence similarity to the *ctpV* and *ctpA* genes of *M. tuberculosis.* In Mtb, treatment with excess copper ions induced upregulation of *ctpV* and other transporters, including permease and sulfate transporter. The resistant strain displayed slower growth, associated with a global reduction in metabolic enzymes and lower intracellular levels of NAD, FAD, mycothiol, and glutamine, indicating metabolic adaptation to copper stress. The mc^2^155-Cu strain also demonstrated increased resistance to isoniazid due to downregulation of the catalase-peroxidase KatG, a protein essential for INH activation [90].

The NicT transporter, belonging to the NiCoT (nickel/cobalt transporter) family, functions not only as a nickel importer but also as an antibiotic efflux pump, facilitating cross-resistance. Expression of NicT in *E. coli* and Msmeg increased nickel uptake by 20.5% and raised the MIC of several antibiotics (norfloxacin, sparfloxacin, ofloxacin, gentamicin, nalidixic acid, and isoniazid) by 2- to 5-fold, conferring greater tolerance to the microorganism. Intracellular accumulation assays showed reduced levels of these antibiotics in cells expressing NicT, indicating efflux activity. Resistance was further enhanced in the presence of sub-inhibitory concentrations of Ni^2+^, suggesting that the ionic gradient generated by metal transport may energetically drive active antibiotic efflux. Site-directed mutagenesis revealed that substitutions in conserved residues of the metal-binding domain (H77, D82, H83, and D227) abolished or reduced both nickel uptake and antibiotic resistance. Molecular docking results supported the idea that NicT possesses a broad binding site capable of interacting with antibiotics through a network of hydrogen bonds and hydrophobic interactions [91].

Copper concentration studies show that Mtb tolerates up to 50 μM without significant impairment but undergoes bactericidal effects at 500 μM, indicating the presence of homeostatic mechanisms. Transcriptomic analyses (microarrays and qRT-PCR) identified a set of 30 Cu-responsive genes, half of which were induced only at toxic concentrations. Among the genes induced at both concentration levels, the cso operon (including *ctpV*), which encodes a P-type ATPase specific for copper export, and the regulator *CsoR* were highlighted. The induction of these genes is highly specific to copper and does not occur to a relevant extent with other metals such as zinc or nickel. *ctpV* shows high similarity to known copper transporters in other organisms. Exposure to toxic cooper levels also induces oxidative stress response genes such as *katG* (catalase-peroxidase) and *furA*, as well as enzymes associated with detoxification and redox homeostasis, including thioredoxin and cysteine synthase. Novel putative transporters and regulatory proteins involved in copper response were also identified, including regulators of the MerR and ArsR families. qRT-PCR confirmed the expression of the *Rv0846c* gene encoding a multicopper oxidase, potentially involved in the conversion of Cu(I) to Cu(II) and in metal storage/export. Thus, beyond CtpV-mediated efflux, the Mtb copper response encompasses multiple layers of both specific and nonspecific defenses, underscoring the role of metal regulation in pathogen virulence and survival during infection [92].

The CtpV protein functions as a copper exporter and is essential for Mtb resistance to copper toxicity. Deletion of the *ctpV* gene resulted in increased copper sensitivity, with mutant bacteria reaching a non-culturable state more rapidly under toxic conditions (500 μM CuCl_2_), whereas complementation restored resistance. Gene expression analyses showed that *ctpV* is induced by high copper levels and that its absence leads to intracellular accumulation of the metal, as evidenced by increased expression of the copper sensor *csoR* and induction of the *cso* operon in Msmeg. Transcriptomic analysis revealed that the absence of CtpV triggers a stress response involving 98 genes associated with oxidative damage, protein denaturation, and metal imbalance, reflecting enhanced cellular stress due to the inability to export copper [93].

In BALB/c mouse and guinea pig models, deletion of *ctpV* did not impair bacterial growth in the lungs but significantly reduced tissue damage and increased survival time of infected animals, despite bacterial loads similar to the wild-type strain. The immune response was also attenuated, with lower IFN-γ expression in the lungs of mice infected with the *ΔctpV* mutant. CtpV-mediated copper response contributes to Mtb pathogenicity primarily by modulating host tissue damage and the inflammatory response [93].

The MntR protein acts as a central regulator of manganese (Mn^2+^) homeostasis in Mtb by mediating Mn^2+^-dependent transcriptional repression, thereby controlling the expression of the manganese transporters MntH (an Nramp-type transporter) and the ABC system MntABCD or OppABCD. *mntR* mutants exhibited hypersensitivity and intracellular accumulation of Mn^2+^ under metal-excess conditions but showed no growth defects under manganese limitation. Deletion of both transport systems, MntH and MntABCD, abolished Mtb growth under manganese-limiting conditions, demonstrating that uptake of this metal is essential for intracellular replication. During macrophage infection, inactivation of MntH and MntABCD impaired Mtb growth, indicating that manganese is a limiting nutrient within the phagosome and that active import of the metal is critical for pathogen survival in this environment. Due to its essentiality for replication and resistance to oxidative stress, the manganese acquisition system constitutes a promising target for anti-tuberculosis strategies based on the nutritional restriction of transition metals [94].

Mtb expresses a specific copper acquisition system mediated by diisonitrile lipopeptides (chalkophores), whose biosynthesis depends on the *nrp* gene cluster (including *nrp*, *fadD10*, and *ppe1*) [95]. Expression of these genes is strongly induced by copper limitation or chelation but not by zinc deficiency or copper excess. Strains deficient in any of these genes exhibit impaired growth and high sensitivity to copper deprivation, but not to zinc, and this phenotype is rescued by genetic complementation or supplementation with a synthetic diisonitrile lipopeptide. Growth assays in 7H9 medium treated with copper chelators (tetrathiomolybdate, TTM, and bathocuproinedisulfonic acid, BCS) showed that mutant strains displayed growth defects, which were restored exclusively by copper supplementation, but not by Zn, Mn, or Fe [95]. Diisonitrile lipopeptides function as chalkophores, ensuring bacterial survival under copper-limiting conditions. Copper limitation is a physiologically relevant stress for Mtb during infection, as deletion of the *nrp* cluster results in attenuated bacterial growth in both zebrafish (Danio rerio) and mouse (C57BL/6) models. The role of the chalkophore system resembles that of siderophores for iron, but it is specific for copper [95].

Copper resistance is essential for Mtb virulence, and the outer membrane channel MctB (mycobacterial copper transport protein B) plays a central role in this process. Mtb mutants deficient in MctB accumulated 100-fold more intracellular copper and were far more sensitive to copper toxicity than the wild-type strain. Similarly, Msmeg mutants lacking the homolog Ms3747 also exhibited increased copper susceptibility, confirming the functional conservation of this system. MctB is crucial for maintaining low intracellular Cu levels and ensuring bacterial growth in the presence of the metal. Deletion of *mctB* did not affect resistance to other metals or alter sensitivity to oxidative or nitrosative stress, indicating specificity for copper. Expression of *mctB* fully restored the wild-type phenotype, whereas partial complementation was attributed to reduced gene expression levels. In guinea pigs infected by aerosol with the *ΔmctB* mutant, bacterial loads were reduced by more than 1000-fold in the lungs and 100-fold in lymph nodes, accompanied by pulmonary granulomas containing copper concentrations 8-fold higher than those in animals infected with the wild-type strain. In mice, the persistence defect of the mutant was exacerbated when the diet was supplemented with copper, indicating that the immune system exploits copper as an antimicrobial defense mechanism. Notably, Mtb is particularly sensitive to copper compared with other bacteria such as *Escherichia coli*, being killed in vitro by metal concentrations lower than those found in the phagosomes of activated macrophages [96].

Compounds of the 4-hydroxyquinoline (4HQ) class and a barbituric acid derivative exhibited bactericidal activity against Mtb in both replicating and non-replicating states. Resistance to these compounds arose through mutations in the *corA* gene, which encodes a Mg^2+^/Co^2+^ transport channel, highlighting the relevance of cobalt in transporter function. Genetic studies and molecular dynamics simulations showed that such mutations maintain the channel in an open conformation, favoring compensatory ion influx and reducing the bactericidal impact of 4HQs [97]. The authors concluded that CorA is not the target of these compounds, but mitigates their bactericidal effect through this compensatory influx. Extracellular cobalt supplementation enhanced bacterial sensitivity to these compounds, in contrast to magnesium, whose increase provided a protective effect only under replicating conditions. CorA (cobalt resistance A) contributes to the homeostasis of divalent metals, including cobalt, which is essential for bacterial survival under drug-induced stress [97]. Magnesium is considered here only as a co-substrate of this channel and not as a metal of nutritional interest in its own right.

**Figure 6:**
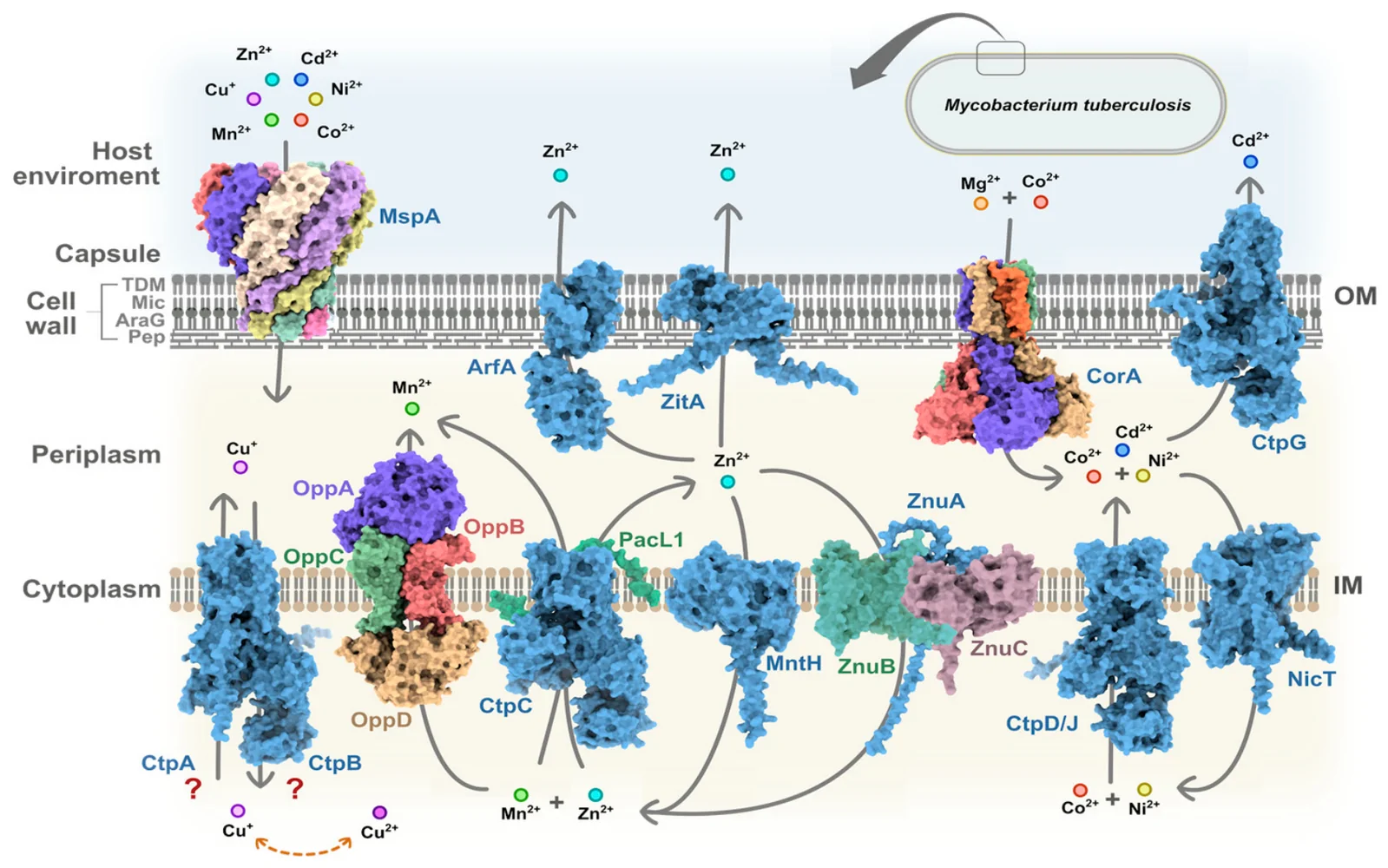
Transport of other transition metals. The image shows the outer (OM) and inner (IM) membranes, as well as the cell wall components peptoglycan (Pep), arabinogalactan (AraG), mycoloyl residue (Mic), and trehalose dimycolate (TDM). The main metals are represented in different colors: manganese (green), cobalt (red), copper (violet), zinc (light blue), cadmium (dark blue), and nickel (yellow). The inner and outer membrane proteins (MspA, ArfA, ZitA, CorA, CtpA, CtpB, CtpC, CtpD, CtpJ, CtpG, OppA-D, MntH, ZnuA-C, NicT) are represented in their crystallographic forms. Some proteins may have more than one location (OM and IM) and are represented here in only one of them. The question marks (?) indicate a stage that the included studies report as not yet established: the direction of copper transport by CtpA and CtpB—a matter for which available data are contradictory, and for which the disruption of these proteins resulted in neither impaired growth nor copper accumulation. This gap in the evidence is discussed in Section 2.2. The representation of the proteins does not maintain the size scale between them. Created in BioRender.com and UCSF ChimeraX 1.3 [25].

Metal transport systems in Mtb and Msmeg, such as P-type ATPases, play crucial roles in the homeostasis of transition metals, confer resistance when these reach toxic concentrations in the host environment, provide protection against oxidative stress, and contribute to bacterial virulence during infection [80,81,82,83,85]. Transporters such as Rv3270, MctB, and NicT, along with porins and efflux platforms containing chaperones, have also been identified as central elements in adaptation to the hostile host environment and in cross-resistance to metals and antibiotics [67,68,87,91,96]. These transport systems represent promising targets for antituberculosis drug development, as their inhibition may compromise bacterial survival and enhance host immune responses. These systems are represented in Figure 6, and Table 4 summarizes the 25 entries related to the transport of other transition metals (Zn, Cu, Mn, Co, Ni, Cd, Pb, and Mg), comprising 15 influx and 9 efflux components, in addition to the bidirectional protein CtpB. P-type ATPases predominate among the efflux systems, and seven entries (ZnuA, ZnuA2, ZnuB, ZnuC, ZnuC2, ZitA, and MspA) originate from *M. smegmatis* rather than *M. tuberculosis*.

**Table 4:** Proteins involved in the transport of other transition metals. ATP: Adenosine triphosphate, ABC: ATP-binding cassette, MspA: Mycobacterial small porin A, Mramp: Mycobacterial resistance-associated macrophage protein, CorA: Cobalt Resistance A, PacL: P-type ATPase-associated chaperone-like protein, ZitA: Zinc transporter A, ZnuA: Zinc uptake protein A, ArfA: Rv0899, also designated OmpATb, *nrp*: Non-ribosomal peptide synthetase, OmpATb: Outer membrane protein A of *Mycobacterium tuberculosis*, Opp: Oligopeptide permease, Mnt: Manganese transport.

Protein Name	Databank Information					Metals	Functional Evidence	Reference
	Gene	PDB/Alpha Fold (AF)	Uniprot ID	Mycobrowser (Locus)	Location/Function			
Copper-exporting P-type ATPase	*ctpA*	AF-P9WPU1-F1	P9WPU1	*Rv0092*	Cell wall Efflux	Zn, Cu, Co, Cd, Pb	Accessory (Cu^+^-stimulated ATPase; no Cu accumulation in Δ*ctpA*)	[77,78,79]
Cation-transporting P-type ATPase B	*ctpB*	AF-P9WPT9-F1	P9WPT9	*Rv0103c*	Inner membrane Influx/Efflux	Zn, Cu, Co, Cd, Pb	Accessory (Cu^+^-stimulated ATPase; growth defect only under Cu limitation)	[77,80,81]
Manganese-exporting P-type ATPase, Metal-transporting ATPase Mta72 (MtaA) ou Cation-transporting P-type ATPase CtpC	*ctpC* or *mtaA*	AF-P9WPT5-F1	P9WPT5	*Rv3270*	Inner membrane Efflux	Zn, Mn	Transport inferred (efflux) (Zn^2+^ by ICP-MS and Mn^2+^ by atomic absorption in mutants; ATPase in micelles; flux not measured)	[67,68,76,77,83]
Probable cobalt/nickel-exporting P-type ATPase	*ctpD*	AF-P9WPT3-F1 (AF-A0R3A7-F1)	P9WPT3 (A0R3A7)	*Rv1469*	Inner membrane Efflux	Zn, Cu, Co, Ni, Cd, Pb	Transport inferred (efflux) (Co^2+^/Ni^2+^ content in mutants; *M. smegmatis;* flux not measured)	[77,84,85]
Probable cation-transporting ATPase G	*ctpG*	AF-P9WPS7-F1	P9WPS7	*Rv1992c*	Inner membrane Efflux	Zn, Co, Cd, Pb	Transport inferred (efflux) (metal-stimulated ATPase in membrane vesicles and metal tolerance; flux not measured)	[76,77,98]
Probable cation-transporting P-type ATPase J	*ctpJ*	AF-P9WPT7-F1	P9WPT7	*Rv3743c*	Inner membrane Efflux	Co, Ni	Transport inferred (efflux) (Co^2+^ content and sensitivity in mutants; Fe^2+^-ATPase activity; flux not measured)	[70,84]
Magnesium transport protein CorA	*corA*	AF-O50455-F1	O50455	*Rv1239c*	Inner membrane Influx	Mg, Co	Accessory (*corA* mutations counteract Mg^2+^ depletion; transport not assayed)	[97]
Nickel transporter (NicT)	*nicT*	AF-I6YEJ7-F1	I6YEJ7	*Rv2856*	Inner membrane Influx	Ni	Transport (influx) (Ni^2+^ uptake by ICP-MS; heterologous expression in *E.coli*)	[91]
Probable copper-exporting P-type ATPase V	*ctpV*	AF-P9WPS3-F1	P9WPS3	*Rv0969*	Cell wall and Inner membrane Efflux	Cu	Transport inferred (efflux) (csoR induction indicating higher intracellular copper; flux not measured)	[92,93]
Porin MspA	*mspA*	1UUN/2V9U/AF-A0QR29-F1	A0QR29	not found	Outer membrane Influx	Zn, Cu, Co, Mn, Ni, Cd, Pb	Accessory for Cu and other divalent metals (porin-dependent Cu susceptibility; metal flux not measured; *M. smegmatis*). For Fe^3+^ see Table 3	[86,87]
Divalent metal cation transporter MntH (Mramp)	*mntH*	AF-P9WIZ5-F1	P9WIZ5	*Rv0924c*	Inner membrane Influx	Zn, Mn	Transport (influx) (^65^Zn^2+^/^55^Fe^2+^ uptake in Xenopus oocytes; Mn^2+^ content by ICP-MS)	[69,94]
Conserved protein	*pacL1*	AF-P96874-F1	P96874	*Rv3269*	Cell wall and Inner membrane Efflux	Zn	Accessory (chaperone-like partner of CtpC; required for Zn^2+^ resistance)	[67]
Cation ABC transporter, periplasmic cation-binding protein, putative	*znuA*	AF-A0R533-F1	A0R533	*Msmeg_6047*	Inner membrane Influx	Zn	Binding/delivery (periplasmic SBP; M.smegmatis)	[88]
Solute-binding lipoprotein	*znuA2*	AF-A0R536-F1	A0R536	*Msmeg_6050*	Inner membrane Influx	Zn	Binding/delivery (periplasmic SBP; M.smegmatis)	[88]
ABC Transition metals transporter, Inner membrane subunit	*znuB*	AF-A0R531-F1	A0R531	*Msmeg_6045*	Inner membrane Influx	Zn	Transport (influx) (permease subunit; FluoZin-3 uptake assay; M.smegmatis)	[88]
Cation ABC transporter, ATP-binding protein, putative	*znuC*	AF-A0R532-F1	A0R532	*Msmeg_6046*	Inner membrane Influx	Zn	Transport (influx) (ATPase subunit; FluoZin-3 uptake assay; *M. smegmatis*)	[88]
ABC transport protein, ATP-binding subunit	*znuC2*	AF-A0R537-F1	A0R537	*Msmeg_6052*	Inner membrane Influx	Zn	Transport (influx) (ATPase subunit; FluoZin-3 uptake assay; *M. smegmatis*)	[88]
Membrane protein ZitA	*zitA*	AF-A0QQG9-F1	A0QQG9	*Msmeg_0750*	not found Efflux	Zn	Accessory (*zitA* disruption causes Zn^2+^ sensitivity; *M. smegmatis*; efflux not demonstrated)	[82,88]
Peptidoglycan-binding protein ArfA	*arfA*, ompATb	2LBT/2LCA/2KSM/2L26/2KGS/2KGW/AF-P9WIU5-F1	P9WIU5	*Rv0899*	Outer membrane, Cell wall, periplasm Not Determined	Zn	Not determined (structural study; channel formation not supported)	[89]
Isonitrile lipopeptide synthase	*nrp*	4U5Q	Q10896	*Rv0101*	Cell wall and Inner membrane Influx	Cu	Accessory (chalkophore biosynthesis required for growth under Cu limitation)	[95]
Copper transporter MctB	MctB	AF-P9WJ83-F1	P9WJ83	*Rv1698*	Outer membrane Efflux	Cu	Transport inferred (efflux) (100-fold copper accumulation in ΔmctB; flux not measured)	[96,99]
Oligopeptide-binding protein OppA or MntA	*oppA*	8J5Q,8J5R,8J5S, 8J5T,U/ AF-P9WGU5-F1	P9WGU5	*Rv1280c*	Inner membrane, periplasm Influx	Mn	Binding/delivery (periplasmic SBP of the Mn^2+^ importer)	[94]
Oligopeptide-binding protein OppB or MntB	*oppB*	8J5Q,8J5R,8J5S,8J5T	P9WFZ7	*Rv1283c*	Inner membrane, periplasm Influx	Mn	Transport inferred (influx) (permease subunit; Mn^2+^ content by ICP-MS; flux not measured)	[94]
Oligopeptide-binding protein OppC or MntC	*oppC*	8J5Q,8J5R,8J5S,8J5T	P9WFZ9	*Rv1282c*	Inner membrane, periplasm Influx	Mn	Transport inferred (influx) (permease subunit; Mn^2+^ content by ICP-MS; flux not measured)	[94]
Oligopeptide-binding protein OppD or MntD	*oppD*	8J5Q,8J5R,8J5S,8J5T	P9WQJ5	*Rv1281c*	Inner membrane, periplasm Influx	Mn	Transport inferred (influx) (ATPase subunit; Mn^2+^ content by ICP-MS; flux not measured)	[94]

Note: The columns under “Databank Information” reflect annotations obtained from UniProt, BioCyc, Mycobrowser, and RCSB PDB. The “Functional Evidence” column summarizes the evidence reported in the studies included in this review: Transport (direct transmembrane transport demonstrated), Transport inferred (metal accumulation or depletion in mutants, or metal-stimulated ATPase activity, consistent with a transport function but without measurement of transmembrane flux), Binding/delivery (substrate binding or delivery demonstrated; transport not demonstrated), Accessory (required for the pathway, but not a transporter itself), and Not determined (association reported without demonstration of binding or transport). The “Metals” column lists the metals reported as being transported or bound in these same studies. Metals are classified into essential transition metals (Fe, Zn, Mn, Co, Ni, Cu), non-essential toxic metals (Cd, Pb), and non-transition metal ions—included because they share a transport pathway with an essential metal (Mg). Any discrepancies between the two sets are discussed in the corresponding section of the text.

### 2.3. Comparison of Candidate Targets

The four tables map 79 protein entries, but the number of candidates supported by the available evidence as therapeutic targets is considerably smaller. Impaired growth or attenuated virulence in a knockout mutant is a necessary but not sufficient criterion. The candidates also differ widely in the conditions under which they were studied and in the extent of their validation, and for this reason we compare them rather than rank them. Table 5 sets them side by side in terms of the functional validation obtained in *M. tuberculosis* itself rather than in a surrogate model, the evidence available from macrophage and animal infection models, the existence of pathways able to compensate for inhibition of the protein, and the key limitations of each. Inhibitor evidence was considered as a fourth axis, but no inhibitor of any of the systems listed is reported in the included studies, so it does not differentiate between them.

These criteria favor points of convergence for pathways that would otherwise be parallel. TnSeq analysis of *M. tuberculosis* cultured in different iron sources revealed that the ESX-3 secretion system is required not only for siderophore-mediated uptake but also for heme utilization. Furthermore, a conditional mutant demonstrated that this system is essential for growth, with the defect being reversed by the addition of iron, zinc, or conditioned supernatant. A system required by both pathways is more difficult to bypass than any individual transporter. The IrtAB complex is supported by the most direct functional evidence in this review, as ATP-dependent Fe3+-mycobactin transport has been reconstituted in proteoliposomes, and the structure of the transporter has been determined via cryo-electron microscopy in various conformational states, which also provides a structural basis for ligand design. Another example is the siderophore export platform formed by MmpL4/MmpL5 and MmpS4/MmpS5, in conjunction with the periplasmic protein Rv0455c. Blocking this secretion not only deprives the bacillus of iron but also leads to the intracellular accumulation of iron-free siderophores. This accumulation exhibits a toxicity comparable to that of isoniazid or rifampicin, rendering the inhibition a self-amplifying process rather than a mere reduction in availability.

These same criteria disqualify candidates that might otherwise appear promising if evaluated solely based on their knockout phenotype. Copper management is distributed among CtpA, CtpB, CtpV, and MctB; disrupting *ctpA* or *ctpB* results in neither growth impairment nor copper accumulation, suggesting that inhibiting a single component would likely be insufficient. Manganese acquisition relies on both MntH and MntABCD, which together serve as the primary importers. The siderophore and heme pathways partially compensate for one another, which diminishes the expected impact of inhibiting a single importer within either pathway and reinforces the case for targeting steps shared between them.

Finally, no inhibitor of any of the systems discussed here is reported in the studies included in this review. The 4-hydroxyquinolines and the barbituric acid derivative that select resistance mutations in *corA* are not CorA inhibitors: the authors of that study concluded that CorA is not their target, but mitigates their bactericidal effect by allowing a compensatory influx of Mg^2+^. Reserpine and spermine were used as an efflux pump inhibitor and a porin inhibitor, respectively; they served as experimental tools rather than as lead compounds. SQ109, the only MmpL3 inhibitor to have reached Phase II clinical trials, functions by blocking the transport of trehalose monomycolate, a mechanism unrelated to metal transport, and therefore does not qualify as chemical matter for the systems discussed here. Candidates characterized solely in *M. smegmatis*, namely ZnuABC, ZnuABC2, ZitA, and MspA, cannot be prioritized until their relevance in *M. tuberculosis* is established; likewise, entries classified as Not determined, such as Rv0899, cannot be considered candidates based on current evidence. The complete absence of reported inhibitors for metal transport systems, when contrasted with the extensive body of genetic and structural evidence compiled here, represents the primary evidence gap identified by this review.

Taken together, these comparisons indicate where the evidence is strongest and what each candidate would require before it could be considered for drug development. The most completely supported systems are those that combine functional validation in *M. tuberculosis* with a phenotype in infection models: ESX-3, whose mutants are attenuated in macrophages and prolong the survival of infected mice; IrtAB, the only entry for which transport has been reconstituted in proteoliposomes and for which cryo-electron microscopy structures are available; and the siderophore export platform, whose disruption lowers the lung burden by four orders of magnitude and whose blockade is self-amplifying through the accumulation of desferrated siderophores. Their principal limitations differ in kind: ESX-3 is essential, so its validation depends on conditional approaches and the species it translocates remains undefined; IrtAB can be bypassed through the heme and transferrin routes; and MmpL4 and MmpL5 are functionally redundant with each other. At the other end of the comparison, copper handling is distributed across four partially redundant components, deletion of *ctpV* does not reduce bacterial burden, and no attenuation has been detected for the *ctpC* mutant in vivo, which limits the value of these proteins as single targets. For the entries characterized only in *M. smegmatis*, and for those whose evidence establishes neither binding nor transport, the first requirement is not inhibitor discovery but validation in *M. tuberculosis* itself. Across all of them the decisive gap is the same: no inhibitor of any of these systems has been reported, so none has yet been tested as a druggable target.

## 3. Materials and Methods

This scoping review was conducted following the JBI Manual for Evidence Synthesis [100] and reported in accordance with the PRISMA-ScR checklist [101] (Supplementary Material, Table S3). The protocol was registered on the Open Science Framework (OSF) platform and is available at https://doi.org/10.17605/OSF.IO/7AC4T (accessed on 5 may 2025).

Eligibility criteria were defined using the Population, Concept, and Context (PCC) framework to address the following question: which biomacromolecules are involved in the transport of heavy metals across the plasma membrane of *M. tuberculosis* and could serve as targets for new therapies? Primary studies on the transport of heavy metals across the plasma membrane of *M. tuberculosis* or *M. smegmatis* were considered eligible; reviews, conference abstracts, editorials, books, and studies in non-Roman scripts were excluded. No date limits were set, and only articles in English were included.

The PubMed, Web of Science, and Embase databases were searched for records published up to March 2025, combining terms related to species, membrane transporters, and heavy metals using the Boolean operators AND and OR; the full search strategy is provided in Supplementary Material Table S4. The reference lists of the selected articles were manually examined. Records were deduplicated using SR-Accelerator and screened in Rayyan by two independent reviewers—first by title and abstract, and subsequently through full-text reading. Disagreements were resolved by consensus or with the involvement of a third reviewer. And the reasons for exclusion listed in the Supplementary Material, Table S5. Data extraction was performed independently by the same two reviewers, using a form adapted from the JBI Data Extraction Tool. Information from databases—UniProt, BioCyc, MycoBrowser, RCSB PDB, and AlphaFold DB—was compiled separately in June 2025, with priority given to the reference strain ATCC 25618/H37Rv. Additional methodological details are provided in Supplementary Material.

## 4. Conclusions

Understanding of transition metal transport and homeostasis in *Mycobacterium tuberculosis* has advanced considerably in recent years. Specialized systems dedicated to the acquisition, efflux, and storage of metals, including iron, copper, zinc, manganese, cobalt, and nickel, are essential for virulence, resistance to host-imposed stress, and metabolic adaptation. Iron homeostasis is the most extensively characterized. Nevertheless, important knowledge gaps persist, particularly regarding siderophore transport across the outer membrane and the precise mechanistic role of the ESX-3 secretion system. HupB, Rv2047c, EsxG, and EsxH may contribute to resolving some of these uncertainties.

Several proteins emerge as candidate pharmacological targets, including IrtAB, the MmpS4/MmpS5-MmpL4/MmpL5 export platform and the periplasmic protein Rv0455c, whose mutants show marked attenuation of Mtb virulence, together with the substrate-binding proteins of the siderophore and heme pathways. For most of the remaining systems, including the copper proteins MctB, CtpV, CtpA and CtpB and the manganese importers MntH and MntABCD, a transport function is inferred from metal accumulation in mutants or from metal-stimulated ATPase activity rather than demonstrated by measurement of transmembrane flux. The ZnuABC zinc importers were characterized in Msmeg, and their relevance to Mtb remains to be confirmed. P-type ATPases are nonetheless relevant, as they supply metals to metalloenzymes that protect the bacterium against oxidative damage, thereby contributing to intracellular survival.

Impaired growth or attenuated virulence in a knockout mutant does not, in itself, qualify a protein as a therapeutic target. The candidates also differ considerably in the conditions under which they were studied and in the extent of their validation, and are therefore compared rather than ranked. Table 5 sets them side by side in terms of the functional validation obtained in *M. tuberculosis*, the evidence available from macrophage and animal infection models, the existence of alternative pathways able to compensate for inhibition, and the key limitations of each. The most completely supported systems are those that combine functional validation with a phenotype in infection models: the ESX-3 secretion system, required for both siderophore-mediated and heme-mediated iron utilization; the IrtAB complex, for which transport has been reconstituted in proteoliposomes and high-resolution structures are available; and the MmpL4/MmpL5-MmpS4/MmpS5 export platform together with Rv0455c, whose blockade leads to the intracellular accumulation of desferrated siderophores. Copper handling is distributed across four partially redundant components and is correspondingly weaker as a single target, while systems characterized solely in *M. smegmatis* require validation in *M. tuberculosis* before they can be considered. Across all of them, no inhibitor of any of these systems is reported in the included studies, which is the principal evidence gap identified by this review.

Despite these advances, uncertainties remain regarding the localization and function of several transporters. Discrepancies among protein databases and experimental analyses, now recorded explicitly in the Functional Evidence column of Table 1, Table 2, Table 3 and Table 4, frequently assign divergent cellular localizations, inner membrane, periplasm, outer membrane, or cell wall, to the same protein. Given the close relationship between localization and function, such inconsistencies complicate the integration of these systems into a coherent model of metal influx and efflux networks. A notable example is CtpB, for which heterologous expression studies under comparable conditions have yielded apparently contradictory effects on bacterial growth. Moreover, the roles of many metal transporters in chronic, latent, or extrapulmonary tuberculosis remain poorly defined. Addressing these gaps will require integrated in vivo functional studies, high-resolution structural analyses, and comprehensive proteomic investigations. Such efforts are essential to fully elucidate metal transport pathways and to harness these systems as innovative therapeutic targets against tuberculosis.

## Figures and Tables

**Table 1:** Proteins involved in iron transport through the siderophore pathway. ATP—Adenosine triphosphate, MmpL—Mycobacterial membrane protein large, PPE—Proline Proline Glutamate, PE—Proline Glutamate, ESAT—Early Secreted Antigenic Target, ESX—Type VII Secretion System.

Protein Name	Databank Information					Functional Evidence	Reference
	Gene	PDB ID/Alpha Fold (AF)	Uniprot ID	Mycobrowser (Locus)	Location/Function		
Mycobactin import ATP-binding/permease protein IrtA	*irtA*	7WIU/7WIV/7WIX/7WIW/9G2K, L, M, V, Y/9G36/9G37/9FXC/9FW3/9G2P, S, T, X, Z/9GL3/AF-P9WQJ9-F1	P9WQJ9	*Rv1348*	Inner membrane Influx	Transport (influx) (ATP-dependent import reconstituted in proteoliposomes)	[34,35,36,37,38,40,41,53,54]
Siderophore interaction domain (SID)	RMCT 1990	6TEJ/6TEK	A0A100XEC2	Not Found	leaflet of the Inner membrane Influx	Accessory (FAD-dependent siderophore reductase domain)	[38]
Mycobactin import ATP-binding/permease protein IrtB	*irtB*	7WIU/7WIV/7WIX/7WIW 9G2K, L, M, V, Y/9G36/9G37/9FXC/9FW3/9G2P, S, T, X, Z/9GL3/AF-P9WQJ9-F1	P9WQJ7	*Rv1349*	Inner membrane Influx	Transport (influx) (ATP-dependent import reconstituted in proteoliposomes)	[35,36,37,38,41,53,54]
Uncharacterized protein Rv2895c	viuB	AF-P9WL31-F1	P9WL31	*Rv2895c*	Inner membrane Influx	Binding/delivery (in vitro; dispensable in vivo)	[36,37,42]
Probable FEIII-dicitrate-binding periplasmic lipoprotein FecB	fecB	8EBG, 7UQ0/AF-O53291-F1	O53291	*Rv3044*	Periplasm Influx	Binding/delivery (periplasmic SBP)	[43]
Siderophore exporter MmpL4	*mmpL4*	9GI0/9GI2/9GI3/9B43/AF-P9WJV3-F1	P9WJV3	*Rv0450c*	Inner membrane Efflux	Transport (efflux) (^14^C-labelled siderophores quantified in cells and culture filtrate)	[26,28,30,55]
Siderophore exporter MmpL5	*mmpL5*	9RFU/9RGB/9B46/9DP6/AF_0AFP9WJV1-F1	P9WJV1	*Rv0676c*	Inner membrane Efflux	Transport (efflux) (^14^C-labelled siderophores quantified in cells and culture filtrate)	[26,28,30,31,32]
Siderophore export accessory protein MmpS4	*mmpS4*	2LW3/AF-P9WJS7-F1	P9WJS9	*Rv0451c*	Inner membrane Efflux	Accessory (periplasmic adaptor of MmpL4)	[26,28,29,30]
Siderophore export accessory protein MmpS5	*mmpS5*	9RFU/9RGB/AF-P9WJS7-F1	P9WJS7	*Rv0677c*	Inner membrane Efflux	Accessory (periplasmic adaptor of MmpL5)	[26,28,29,30,31]
Periplasmic siderophore secretion protein Rv0455c. Conserved protein	rv0455c	8DRG, 8DQY, 8DRI/AF-O53740-F1	O53740	*Rv0455c*	Periplasm Efflux	Accessory (required for siderophore secretion)	[28,33]
Uncharacterized protein Rv2047c	Not Found	AF-P9WIH5-F1	P9WIH5	*Rv2047c*	Periplasm Cell Wall Efflux	Not determined	[32]
DNA-binding protein HupB	*hupB*	4DKY/4PT4/AF-P9WMK7-F1	P9WMK7	*Rv2986c*	Cell wall, periplasm Inner membrane Influx	Binding/delivery (surface receptor for Fe^3+^-cMyc)	[50]
Multidrug efflux pump Tap	tap	AF-P9WJX9-F1	P9WJX9	*Rv1258c*	Inner membrane Efflux	Not determined	[51]
Putative TonB-dependent transporter (FecA homolog)	fecA	Not found	SGD04621.1 (NCBI)	Not found	Outer membrane Influx	Not determined (Strain 2926STDY5723586)	[52]
ESX-3 secretion system protein EccA3	eccA3	7NAZ/AF-P9WPI3-F1	P9WPI3	*Rv0282*	Inner membrane Influx	Accessory (ESX-3 component)	[47,48,56,57]
ESX-3 secretion system ATPase EccB3	*eccB3*	AF-P9WNR3-F1	P9WNR3	*Rv0283*	Inner membrane Efflux	Accessory (ESX-3 component)	[47]
ESX-3 secretion system protein EccC3	*eccC3*	6J17/AF-P9WNA9-F1	P9WNA9	*Rv0284*	Inner membrane Efflux	Accessory (ESX-3 component)	[47]
ESX-3 secretion system protein EccD3	*eccD3*	AF-P9WNQ3-F1	P9WNQ3	*Rv0290*	Inner membrane Efflux	Accessory (ESX-3 component)	[46]
ESX-3 secretion system protein EccE3	*eccE3*	AF-P9WJE5-F1	P9WJE5	*Rv0292*	Inner membrane Efflux	Accessory (ESX-3 component)	[46]
ESX-3 secretion-associated protein EspG3	espG3	5XKL/AF-P9WJC7-F1	P9WJC7	*Rv0289*	Inner membrane Efflux	Accessory (ESX-3 chaperone)	[46]
ESAT-6-like protein EsxG	esxG	2KG7/AF-O53692-F1	O53692	*Rv0287*	Inner membrane Efflux	Accessory (secreted ESX-3 substrate)	[48]
ESAT-6-like protein EsxH	esxH	2KG7/AF-P9WNK3-F1	P9WNK3	*Rv0288*	Inner membrane Efflux	Accessory (secreted ESX-3 substrate)	[48]
Mycosin-3	*mycP3*	4KG7/AF-O53695-F1	O53695	*Rv0291*	Inner membrane Efflux	Accessory (ESX-3 protease)	[46]
PE family immunomodulator PE5	PE5	6UUJ, 6VHR/AF-L7N695-F1	L7N695	*Rv0285*	Inner membrane Influx	Accessory (ESX-3 substrate)	[48,49]
PPE family protein PPE4	PPE4	6UUJ, 6VHR/AF-P9WI43-F1	P9WI43	*Rv0286*	Inner membrane Influx	Accessory (ESX-3 substrate)	[32,48,49]

Note: The columns under “Databank Information” reflect annotations obtained from UniProt, BioCyc, Mycobrowser, and RCSB PDB. The “Functional Evidence” column summarizes the evidence reported in the studies included in this review: Transport (direct transmembrane transport demonstrated), Transport inferred (metal accumulation or depletion in mutants, or metal-stimulated ATPase activity, consistent with a transport function but without measurement of transmembrane flux), Binding/delivery (substrate binding or delivery demonstrated; transport not demonstrated), Accessory (required for the pathway, but not a transporter itself), and Not determined (association reported without demonstration of binding or transport). Any discrepancies between the two sets are discussed in the corresponding section of the text.

**Table 2:** Proteins involved in iron transport through the heme pathway. PPE: Proline Proline Glutamate, PE: Proline Glutamate, NYN: Asparagina Tirosina Asparagina, MmpL: Mycobacterial membrane protein large, ABC: ATP-binding cassette, ESX: TypeVII Secretion System.

Protein Name	Databank Information					Functional Evidence	Reference
	Gene	PDB ID/Alpha Fold (AF)	Uniprot ID	Mycobrowser (Locus)	Location/Function		
PE family protein PE22	*PE22*	AF-L7N6B5-F1, AF-A0A045IN32-F1	L7N6B5	*Rv2107*	Outer membrane Influx	Binding/delivery (surface heme-binding, with PPE36)	[58]
Uncharacterized PPE family protein PPE36	*PPE36*	AF-P9WI01-F1	P9WI01	*Rv2108*	Outer membrane Influx	Binding/delivery (surface heme-binding)	[58,64]
Uncharacterized PPE family protein PPE37	*PPE37*	AF-Q79FH3-F1	Q79FH3	*Rv2123*	Inner membrane Influx	Accessory (essential for heme-iron acquisition; mechanism not determined)	[59]
Uncharacterized PPE family protein PPE62	*PPE62*	AF-P9WHX7-F1	P9WHX7	*Rv3533c*	Outer membrane Influx	Binding/delivery (surface heme-binding)	[58]
PPE family protein PPE64	*PPE64*	AF-Q6MWW0-F1	Q6MWW0	*Rv3558*	Inner membrane Influx	Binding/delivery (binds heme; forms channels in lipid bilayer)	[64]
Probable periplasmic dipeptide-binding lipoprotein DppA	*dppA*	6E3D, 6E4D, 7JLS, 8WD9, 8WDA, 8WDB, 8XFC/AF-I6×811-F1	I6X811	*Rv3666c*	Periplasm Influx	Binding/delivery (periplasmic SBP)	[58]
Probable dipeptide-transport integral membrane protein ABC transporter DppB	*dppB*	8WD9, 8WDA, 8WDB, 8XFC/AF-I6YGV9-F1	I6YGV9	*Rv3665c*	Inner membrane Influx	Transport inferred (influx) (permease subunit; growth and utilization phenotypes; flux not measured)	[58]
Probable dipeptide-transport integral membrane protein ABC transporter DppC	*dppC*	8WD9, 8WDA, 8WDB, 8XFC/AF-L0TEV4-F1	L0TEV4	*Rv3664c*	Inner membrane Influx	Transport inferred (influx) (permease subunit; growth and utilization phenotypes; flux not measured)	[58]
Probable dipeptide-transport ATP-binding protein ABC transporter DppD	*dppD*	8WD9, 8WDA, 8WDB, 8XFC/AF-I6Y482-F1	I6Y482	*Rv3663c*	Inner membrane Influx	Transport inferred (influx) (ATPase subunit; growth and utilization phenotypes; flux not measured)	[58]
Trehalose monomycolate exporter MmpL3	*mmpL3*	7NVH/AF-P9WJV5-F1	P9WJV5	*Rv0206c*	Inner membrane Influx	Binding/delivery (heme binding by extracellular domain)	[60,61]
Heme uptake protein MmpL11	*mmpL11*	AF-P9WJT9-F1	P9WJT9	*Rv0202c*	Inner membrane Influx	Accessory (required for heme uptake; transport not demonstrated)	[60,61]
Heme-binding protein	Rv0203	3MAY/AF-I6X8R5-F1	I6X8R5	*Rv0203*	Periplasm Influx	Binding/delivery (secreted heme carrier)	[60,61]
Probable conserved transmembrane protein	Not Found	AF-I6Y748-F1	I6Y748	*Rv0204c*	Inner membrane Influx	Not determined	[61]
Putative transport protein Rv0205	Not Found	AF-P9WFM5-F1	P9WFM5	*Rv0205*	Inner membrane Influx	Not determined	[61]
NYN domain-containing protein	Not Found	AF-P96389-F1	P96389	*Rv0207c*	Inner membrane Influx	Not determined	[61]
Heme oxygenase (mycobilin-producing)	MhuD	3HX9, 4NL5, 5UQ4, 6DS7, 6DS8, 6PLE/AF-P9WKH3-F1	P9WKH3	*Rv3592*	Cell wall, Inner membrane Influx	Accessory (cytoplasmic heme oxygenase)	[58]
Probable periplasmic iron-transport lipoprotein (MhuP)	*fecB2*	7FHP, 7FHM, 4PM4/AF-L7N6B2-F1	L7N6B2	*Rv0265c*	Periplasm Influx	Binding/delivery (periplasmic heme-binding SBP)	[43,63]
ESX-4 secretion system protein EccC4	*eccC4*	AF-P9WNA7-F1	P9WNA7	*Rv3447c*	Inner membrane Influx	Accessory (ESX-4 component)	[64]
Probable serine protease PepA (Serine proteinase) (MTB32A)	pepA	AF-O07175-F1	O07175	*Rv0125*	Inner membrane Influx	Accessory (secreted; required for efficient heme utilization)	[64]
UPF0073 membrane protein Rv1085c	Not Found	AF-P9WFN7-F1	P9WFN7	*Rv1085c*	Inner membrane Influx	Accessory (secreted; required for efficient heme utilization)	[64]

Note: The columns under “Databank Information” reflect annotations obtained from UniProt, BioCyc, Mycobrowser, and RCSB PDB. The “Functional Evidence” column summarizes the evidence reported in the studies included in this review: Transport (direct transmembrane transport demonstrated), Transport inferred (metal accumulation or depletion in mutants, or metal-stimulated ATPase activity, consistent with a transport function but without measurement of transmembrane flux), Binding/delivery (substrate binding or delivery demonstrated; transport not demonstrated), Accessory (required for the pathway; transmembrane transport not demonstrated), and Not determined (association reported without demonstration of binding or transport). Any discrepancies between the two sets are discussed in the corresponding section of the text.

**Table 5:** Comparison of candidate targets among the transport systems mapped in this review, in terms of functional validation, evidence from infection models, alternative pathways and key limitations. ICP-MS: inductively coupled plasma mass spectrometry; TnSeq: transposon mutagenesis coupled with deep sequencing;.

Target	Functional Validation	Evidence in Infection Models	Alternative Pathways	Key Limitations	Reference
ESX-3 secretion system (EccB3, EccC3, EccD3, EsxG, EsxH, MycP3, PE5-PPE4)	Accessory. Essential for growth in a conditional mutant, with the defect reversed by iron, by zinc or by conditioned supernatant; TnSeq showed the system is required for both siderophore- and heme-mediated iron utilization.	Δesx-3 and ΔesxH highly attenuated in human macrophages; median survival of infected mice 65 days for ΔesxH versus 22 days for the wild type, largely reversed by complementation. Δesx-3 more severely attenuated (>12 months).	None identified. The system is shared by the siderophore and the heme routes.	Essentiality requires conditional approaches; the translocated species is not defined; ESX-3 is a secretion machine, not a transporter, required for but not directly mediating mycobactin-dependent iron uptake.	[32,45,46,47,48,49,57]
IrtAB (IrtA, IrtB and the siderophore interaction domain)	Transport (influx). ATP-dependent import of Fe3+-mycobactin reconstituted in proteoliposomes; cryo-EM structures resolved in several conformational states.	irtAB mutant attenuated in human macrophages and in mouse lungs.	Heme-mediated uptake; GAPDH-mediated acquisition of transferrin-bound iron.	Iron can also be obtained through the heme route (independent of IrtAB), while transferrin utilization requires siderophores and therefore depends on IrtAB; no inhibitor has been reported	[34,35,36,37,38,41,54]
Siderophore export platform (MmpL4/MmpL5 with MmpS4/MmpS5) and Rv0455c	Transport inferred (efflux) for MmpL4/MmpL5, based on reduced siderophore secretion in deletion mutants (^14^C-TLC) and structural evidence of a mycobactin binding site. Accessory for MmpS4/MmpS5; essential for Rv0455c. The system is essential for siderophore secretion and for virulence in mice.	The ΔmmpS4/ΔmmpS5 double mutant showed a >24,000-fold lower bacterial burden in mouse lungs and no visible lung pathology.	The MmpL4 and MmpL5 complexes are functionally redundant with each other, but the pair is not.	MmpL4 and MmpL5 are functionally redundant with each other, so both would have to be blocked; no inhibitor reported.	[26,28,29,30,33]
Dpp heme uptake system (DppA, DppB, DppC, DppD)	Binding/delivery for DppA (heme binding abolished by the Arg179 mutation); Transport inferred (influx) for DppB, DppC and DppD, based on growth and utilization phenotypes; transmembrane flux was not measured.	Impaired heme utilization in THP-1 macrophages; mouse infection not reported for the dpp mutant.	Siderophore and albumin pathway mediated uptake.	Transport mediated by the permease and ATPase subunits is inferred rather than measured, and the albumin pathway compensates for the loss of this mechanism.	[58,63]
Manganese importers MntH and MntABCD (OppA-D)	Transport (influx) for MntH (^65^Zn^2+^/^55^Fe^2+^ uptake in Xenopus oocytes); Transport inferred for MntABCD (manganese content by ICP-MS). The two systems were identified as the main Mn^2+^ importers of Mtb.	Inactivation of MntABCD partially attenuated Mtb in macrophages; mntR mutants, which constitutively express import functions, were unaffected in macrophages and in mice.	The two systems can replace each other, but with unequal contributions.	The two systems substitute for each other, and constitutive expression of import functions produced no phenotype in macrophages or in mice.	[69,94]
CtpC with the chaperone-like protein PacL1	Transport inferred (efflux). Zn^2+^ accumulation in the ctpC mutant by ICP-MS, Mn^2+^ accumulation by atomic absorption, and metal-stimulated ATPase activity of the protein reconstituted in micelles; transmembrane flux was not measured.	No attenuation of the ctpC null mutant was detected in immunocompetent or immunodeficient animals at high inoculum.	Other zinc efflux routes (other P-type ATPases, PacL2/PacL3–CtpG/CtpV).	The assignment of the substrate between Zn^2+^ and Mn^2+^ is a subject of controversy; transmembrane flux has not been measured, and in vivo attenuation was detected only in the mutant strain within the C57BL/6 background.	[67,68,76,83]
CorA	Accessory. Resistance mutations in corA were selected by 4-hydroxyquinolines and a barbituric acid derivative; the authors concluded that CorA is not the target of these compounds but mitigates their bactericidal effect through a compensatory influx of Mg^2+^. No transport assay was performed.	Not assessed; the compounds were evaluated only in vitro.	Other divalent cation routes.	The compounds that select corA mutations do not act on CorA; no transport assay and no infection model are available.	[97]
NicT	Transport (influx). Nickel uptake measured by ICP-MS in *E. coli* expressing NicT, with residue substitutions abolishing uptake; antibiotic efflux shown in mycobacteria.	Not assessed.	Other nickel routes not characterized.	Transport was demonstrated only in a heterologous host, and no infection model has been reported.	[91]
Copper systems (MctB, CtpV, CtpA, CtpB)	Transport (efflux) is inferred for MctB (with a 100-fold greater copper accumulation in the mutant) and for CtpV (as indicated by higher intracellular copper levels, evidenced by the induction of CsoR); an accessory function is suggested for CtpA, as its deletion resulted in neither growth impairment nor copper accumulation. For CtpB, a growth defect was observed under low-copper conditions and within adipocytes. Transmembrane flux was not measured for any of these proteins.	mctB mutant showed 1000-fold and 100-fold lower burden in lungs and lymph nodes of guinea pigs; the ctpV mutant reached wild-type levels in mice and guinea pigs, with reduced lung damage and longer survival.	Four partially redundant components, in addition to cytoplasmic buffering by the metallothionein MymT.	Four partially redundant components plus cytoplasmic buffering by MymT; deletion of ctpV does not reduce bacterial burden.	[78,79,80,92,93,96]
ZnuABC, ZnuABC2, ZitA and MspA	Transport (influx) for MspA and the ZnuABC subunits, based on uptake assays, or Binding/delivery; demonstrated in *M. smegmatis* only.	Not assessed; the evidence was obtained in *M. smegmatis.*	Not assessable in *M. tuberculosis.*	Characterized only in *M. smegmatis*; relevance to *M. tuberculosis* is not established.	[82,86,87,88]
Rv1258c (Tap) and Rv0899 (ArfA/OmpATb)	Not determined for Rv1258c (association reported without demonstration of binding or transport; *M. tuberculosis*); Binding/delivery for Rv0899 (Zn^2+^ binding shown in vitro; no transport assay; M. tuberculosis protein expressed in *E. coli*).	Not assessed.	Not applicable.	Rv1258c—Neither binding nor transport has been demonstrated, Rv0899-binding shown, transport not assessed.	[51,68,89]

Note: The “Functional validation” column reproduces the controlled vocabulary applied in Table 1, Table 2, Table 3 and Table 4 (Transport, Transport inferred, Binding/delivery, Accessory and Not determined) and states the experimental basis of each assignment, identifying the organism whenever the evidence was not obtained in *M. tuberculosis*. The “Evidence in infection models” column reports the macrophage and animal infection data available in the included studies. Inhibitor evidence was also considered as a comparison axis, but no inhibitor of any of the systems listed is reported in those studies, so this axis does not differentiate between the targets and no column for it is included. The “Key limitations” column states the main constraint on each target as reported in the included studies. The targets are compared and not ranked, because they differ considerably in experimental conditions and in the extent of validation.

## Data Availability

No new data were created or analyzed in this study.

## Supplementary Materials

The following supporting information can be downloaded at https://www.mdpi.com/article/10.3390/molecules31213992/s1. This supplementary material provides: Table S1—Main therapeutic targets and approved or investigational antituberculosis agents; Table S2—Data extraction table; Scoping review methodology; Table S3—Preferred Reporting Items for Systematic reviews and Meta-Analyses extension for Scoping Reviews (PRISMA-ScR) Checklist; Table S4—Databases, terms and operators used in the search strategy; Table S5—Excluded studies, after full-text reading, and justifications. References [102,103,104,105] are cited only in the Supplementary Material.
