# Supplementary material for: The Essential Transition Metals Transport Systems in Mycobacterium tuberculosis: A Scoping Review of Emerging Targets for Antitubercular Drug Discovery
Source: Molecules. 2026 Nov 5;31(21):3992. doi: 10.3390/molecules31213992 (PMC13764973; doi:10.3390/molecules31213992)
Supplement: Supplementary file 1 [file molecules-31-03992-s001.zip › molecules-4605752-supplementary.pdf]

Supplementary Material—Molecules

The Essential Transition Metals Transport Systems in *Mycobacterium tuberculosis*: A Scoping Review of Emerging Targets for Antitubercular Drug Discovery

Moisés Maia Neto, Laura Helena Schroeder Borges, Gustavo Torres Corrêa, Cristiane Cunha Frota, Raul Edison Luna Lazo and Roberto Pontarolo *

* Correspondence: pontarolo@ufpr.br
Pharmaceutical Sciences Postgraduate Program, Department of Pharmacy, Federal University of Paraná, Campus III, Av. Pref. Lothário Meissner, 632, Jardim Botânico, Curitiba 80210-170, PR, Brazil.

Targets and Antituberculosis agents

Table S1. Main therapeutic targets and approved or investigational antituberculosis agentes.

Targets (UniProt-preferred name) (Uniprot ID)	Genes (locus, H37Rv)	Function	Antituberculosis Agents
**First-line antitubercular agentes:**			
Enoyl-[acyl-carrier-protein] reductase [NADH] P9WGR1	inhA (Rv1484)	Fatty-acid elongation (FAS-II); essential for mycolic-acid biosynthesis	Isoniazid and Ethionamide
Catalase-peroxidase P9WIE5	katG (Rv1908c)	Activates isoniazid to isonicotinic-acyl-NADH; oxidative-stress defense.	Isoniazid
3-oxoacyl-[acyl-carrier-protein] synthase 1 (KasA) P9WQD9	kasA (Rv2245)	Chain elongation in FAS-II during mycolic-acid biosynthesis.	Isoniazid
Alkyl hydroperoxide reductase C (AhpC) P9WQB7	ahpC (Rv2428)	Detoxifies organic hydroperoxides; compensatory mechanism in katG mutants.	Isoniazid (indirect/compensatory)
Type II NADH:quinone oxidoreductase Ndh (NDH-2) P95160	ndh (Rv1854c)	Respiratory chain; oxidizes NADH to NAD^+; impacts INH activation via redox.	Isoniazid, Clofazimine (redox/ETC effects)
FAD-containing monooxygenase EthA P9WNF9	ethA (Rv3854c)	Activates ethionamide/prothionamide to NAD adducts that inhibit InhA.	Ethionamide, Prothionamide
HTH-type transcriptional regulator EthR P9WMC1	ethR (Rv3855)	Represses ethA; EthR inhibitors boost ETH/PTH activation.	Ethionamide, Prothionamide (synergy via EthR inhibitors)
RNA polymerase β-subunit P9WGY9	rpoB (Rv0667)	Transcription of DNA into RNA.	Rifampicin, Rifapentine
Nicotinamidase/pyrazinamidase I6XD65	pncA (Rv2043c)	Converts pyrazinamide (PZA) to active pyrazinoic acid.	Pyrazinamide
Small ribosomal subunit protein bS1 P9WH43	rpsA (Rv1630)	Binds pyrazinoic acid (POA), the active form of the prodrug pyrazinamide (PZA);	Pyrazinamide
Arabinosyltransferases (EmbABC complex) P9WNL9 P9WNL7 P9WNL5	embA (Rv3794), embB (Rv3795), embC (Rv3793)	Arabinogalactan biosynthesis in cell wall.	Ethambutol
**Second-line antitubercular agentes:**			

16S/23S rRNA (cytidine-2′-O)-methyltransferase TlyA; Small ribosomal subunit protein uS12; Ribosomal RNA small subunit methyltransferase G<br>P9WJ63<br>P9WH63<br>P9WGW9	tlyA (Rv1694), rpsL (Rv0682), rsmG (Rv3919c)	Protein synthesis (aminoglycoside binding); gidB methylates 16S.	Aminoglycosides (Streptomycin, Amikacin, Kanamycin)
DNA gyrase subunit AB<br>P9WG47<br>P9WG45	gyrA (Rv0006), gyrB (Rv0005)	DNA replication/supercoiling.	Fluoroquinolones (Levofloxacin, Moxifloxacin)
Large ribosomal subunit protein uL3/L4<br>P9WH87<br>P9WH85	rplC (Rv0701), rplD (Rv0702)	Protein synthesis (peptidyl-transferase center).	Linezolid
L,D-Transpeptidases<br>O53638<br>I6Y9J2	ldtA (Rv0116c)/ldtB (Rv2518c)	3→3 peptidoglycan cross-linking.	Carbapenems (Meropenem)
Alanine racemase/D-alanine--D-alanine ligase<br>P9WQA9<br>P9WP31	alr (Rv3423c), ddlA (Rv2981c)	Peptidoglycan precursor synthesis.	Cycloserine/Terizidone
ATP synthase subunit c<br>P9WPS1	atpE (Rv1305)	Energy metabolism (oxidative phosphorylation).	Bedaquiline
Deazaflavin-dependent nitroreductase/F420 pathway enzymes/Phosphoenolpyruvate transferase<br>P9WP15<br>P9WNE1<br>P9WP81/P9WP79	ddn (Rv3547), fgd1 (Rv0407), fbiA/B/C (Rv3261/Rv3262/Rv1173)	Prodrug activation; respiratory poisoning & mycolic-acid effects; NO release.	Delamanid, Pretomanid
**Other agents:** repurposing or new agents in clinical trials			
Trehalose monomycolate transporter (MmpL3)	mmpL3 (Rv0206c)	Mycolate/TMM transport across inner membrane.	SQ109, Rimonabant
WecA (UDP-GlcNAc transferase)	wecA (Rv1302)	Initiates arabinogalactan biosynthesis (Lipid I).	CPZEN-45
Decaprenylphosphoryl-β-D-ribose 2′-epimerase (DprE1)	dprE1 (Rv3790)	Arabinogalactan/lipoarabinomannan precursor synthesis.	BTZ043, PBTZ169 (Macozinone), TBA-7371, OPC-167832
Cytochrome bc1 (QcrB)/aa3 oxidase	qcrB (Rv2196), cta complex	Electron transport chain; energy metabolism.	Telacebec (Q203), TB47, Lansoprazole sulfide
Cytochrome bd oxidase	cydA/cydB	Terminal oxidase under hypoxia/stress; energy metabolism.	Aurachin D (investigational)
ATP synthase, c-subunit	atpE (Rv1305)	Energy metabolism (oxidative phosphorylation).	TBAJ-587, TBAJ-876, squaramide derivatives (investigational)
DNA topoisomerase I	topA (Rv3646c)	DNA relaxation during replication/transcription.	Imipramine, Norclomipramine (repurposing; investigational)
NAD-dependent DNA ligase (LigA)	ligA (Rv3014c)	DNA replication/repair sealing of nicks.	Pyridochromanone derivatives; (reports with Chloroquine analogs)
Thymidine kinase/Thymidylate kinase	tdk (Rv2697c)/tmk (Rv1712)	Nucleotide metabolism; DNA synthesis.	Nucleoside analogs (e.g., AZT)
3-oxoacyl-[acyl-carrier-protein] synthase 1 (KasA)<br>P9WQD9	kasA (Rv2245)	Chain elongation in FAS-II during mycolic-acid biosynthesis.	Thiolactomycin , JSF-3285

Large ribosomal subunit protein uL3/L4 P9WH87 P9WH85	rplC (Rv0701), rplD (Rv0702)	Protein synthesis (peptidyl-transferase center).	Sutezolid, Delpazolid, Contezolid, Tedizolid TBI-223
**Other agents under investigation:** BVL-GSK098, BTZ-043, GSK-286 (GSK 2556286), GSK-3036656, macozinone, OPC-167832, TBI-166, sanfetrinem, BM212, SPR720 (fobrepodacin) and sudapyridine (WX-081)			

Note: The data presented in this table were adapted from references [1], [7], [19], and [102] described in the main manuscript.

Scoping review methodology

Protocol and Registration:

This scoping review was conducted based on the JBI Handbook for Evidence Synthesis [100] and reported according to the PRISMA-ScR checklist [101] (Table S3). The protocol was registered with the Open Science Framework (OSF) platform and is available at https://osf.io/, under DOI: 10.17605/OSF.IO/7AC4T.

Information sources:

This review was conducted using the PubMed, Web of Science, and Embase databases. Records were considered up to March 2025. To obtain information on protein nomenclature, genomics, crystallographic structure, computational and structural proteomics databases were used. Searches were conducted in June 2025 and included the following databases: UniProt [3], MycoBrowser [103], BioCyc [20], AlphaFold [104], and RCSB PDB [21]. Record selection prioritized, when available, the wild-type and virulent reference strain ATCC 25618/H37Rv.

Search strategy:

A detailed search strategy was defined, which included keywords associated with Species/Pathology (Mycobacterium tuberculosis and Mycobacterium smegmatis), Membrane Transporter, and Heavy Metals, using Boolean operators AND and OR. The detailed search strategy is described in Table S4. The reference lists of the selected articles were also manually checked to ensure the inclusion of all relevant studies. This search strategy included articles in English, with no time limits.

Eligibility Criteria:

Eligibility criteria were defined based on the acronym Population, Concept, and Context (PCC) [5], used to formulate the guiding question: "Which biomacromolecules are involved in the transport of heavy metals across the plasma membrane of M. tuberculosis and could be used as promising targets for the development of new therapies?" This review included primary studies addressing the transport of heavy metals across the plasma membrane of M. tuberculosis or M. smegmatis (Msmeg). Reviews, abstracts, editorials, books, and studies written in non-Roman characters were excluded.

Study selection:

The selection process was divided into two phases, the first dedicated to searches in the aforementioned databases. Subsequently, the retrieved lists were downloaded and entered into the SR-Accelerator platform to exclude duplicates [13]. The resulting list was then incorporated into the free version of the Rayyan web app [105], where two independent reviewers applied the eligibility criteria by reading titles and abstracts. The second stage consisted of analyzing the full text of articles that met the inclusion criteria to determine their eligibility for the review. Any discrepancies were resolved by a third reviewer during the screening and eligibility stages. Articles that did not meet the

eligibility criteria were excluded, and the reasons for these exclusions are duly recorded in Table S3.

Data Extraction:

The data from the included studies were extracted by the two reviewers into a Microsoft Excel 2019 spreadsheet and subsequently transferred to a Microsoft Office Word 2019 table (Supplementary Material, Table S2), adapted from the JBI Data Extraction Tool to better represent the type of data presented in these studies [100]. The main transporter biomacromolecules, the transporter family to which they belong, the transported metals, the type of transport performed, structural information, and experimental information were recorded. Due to the heterogeneous nature of the studies, it was necessary to classify them according to their design and objectives, whether they focused on the identification and structural characterization of transporters or genomic studies focused on the function of biomacromolecules. Following the completion of the data mapping, any discrepancies were resolved through consensus meetings and, if necessary, with the participation of a third reviewer. Finally, the data were organized into tables, diagrams and graphs and subsequently analyzed and discussed.

Data collection from databases and literature references. For each biomacromolecule included in this synthesis, two independent sets of information were gathered and are presented side-by-side in Tables 1 through 4 of the main text.

Data from databases were compiled in June 2025 using UniProt, BioCyc, MycoBrowser, the RCSB PDB, and the AlphaFold DB, prioritizing the virulent wild-type reference strain ATCC 25618/H37Rv whenever available. Structural records were obtained from the RCSB PDB and the AlphaFold DB; accession numbers from UniProt; and gene and locus designations from BioCyc and MycoBrowser. Two fields were not transcribed verbatim but were instead inferred by the reviewers based on the content of these records. The direction of transport (influx or efflux) was inferred based on the molecular function assigned in UniProt—most commonly "translocase," but also "porin," "chaperone," "oxidoreductase," "hydrolase," or "DNA-binding"—in conjunction with the transport reaction described in the corresponding record; in cases where the record did not allow for such an assignment, the field was classified as "undetermined." Subcellular localization was inferred from BioCyc, retaining the evidence qualifiers used by that source, which allows for the distinction between localizations supported by experimental data and those derived from computational predictions.

Information from literature references was collected independently—drawn from the studies included in this review rather than from databases. For each protein, the functional role was classified based on the most robust evidence reported in these studies, using four categories: "Transport" (when direct transmembrane translocation of the metal was demonstrated); "Binding/delivery" (when binding to the substrate or its transfer to a transport system was demonstrated, but not translocation); "Accessory" (when the protein is required for the pathway but does not perform metal transport, such as components of secretion systems, reductases, and chaperones); and "Undetermined" (when an association with the pathway was reported, but neither binding nor transport was demonstrated).

The assessment was conducted independently by two reviewers for both datasets; any discrepancies were resolved by consensus or, when necessary, with the assistance of a third reviewer. Presenting the two sets separately allows for the direct observation of agreement or divergence between database annotations and experimental evidence for each entry—a fact that, in itself, constitutes one of the findings of this review.

Table S2. Data extraction table.

Reference	Protein	Gene	Metal Transported	Location	Function	Structural Information	Genomic/Transcriptomic Information	Functional Evidence and Validation (This Review)	Evidence in Infection Models	Alternative Pathways	Key Limitations
Adhikary, 2022 [91]	NicT	Rv2856	Nickel (Ni^{2+})	Cell membrane	Ni^{2+} influx and antibiotic efflux	NicT is a transporter of the NiCoT family, with a dual function: it transports Ni^{2+} ions and acts as an antibiotic efflux pump. Targeted mutagenesis in conserved residues (H77I, D82I, H83L, D227I) resulted in a significant loss of transport activity, demonstrating that these regions are critical for the formation of the binding site and translocation of the ion/metabolite. Structural modeling reinforced the role of these residues in the functional architecture of the transporter.	Overexpression of NicT in E. coli and M. smegmatis increased nickel uptake and conferred greater tolerance to antibiotics (norfloxacin, sparfloxacin, ofloxacin, gentamicin, nalidixic acid, and isoniazid), especially in the presence of Ni^{2+}. The mutation of conserved residues abolished this phenotype, reducing both nickel uptake and antibiotic tolerance, confirming the functional role of the gene. There was no change in sensitivity to other antibiotics tested, except under subinhibitory concentrations of Ni^{2+}, suggesting a metal-dependent regulatory mechanism.	Transport (influx). Nickel uptake measured by ICP-MS in E. coli expressing NicT, with residue substitutions abolishing uptake; antibiotic efflux shown in mycobacteria.	Not assessed.	Other nickel routes not characterized.	Transport was demonstrated only in a heterologous host, and no infection model has been reported.
Agranoff, 1999 [69]	Mramp	mramp	Iron (Fe^{2+}), Zinc (Zn^{2+}), Manganese (Mn^{2+}), Copper (Cu^{2+})	Membrane (Nramp)	Influx of divalent cations	Mramp is a bacterial homologue of the Nramp family, identified in M. tuberculosis and M. bovis. Heterologous expression of Mramp RNA in Xenopus laevis oocytes increased Fe^{2+} and Zn^{2+} uptake by 20-fold, proving its transport activity. Mramp-mediated	Mramp expression is regulated by iron and copper availability: there is an increase in mramp mRNA in response to increasing concentrations of Fe^{2+} and Cu^{2+} (1-5 μM), parallel to the regulation of a gene encoding a metal-transporting ATPase. The study used semi-quantitative RT-PCR and ratio-	Transport (influx) for MntH ($^{65}Zn^{2+}$/$^{55}Fe^{2+}$ uptake in Xenopus oocytes); Transport inferred for MntABCD (manganese content by ICP-MS). The two systems were identified as the main Mn^{2+} importers of Mtb.	Inactivation of MntABCD partially attenuated Mtb in macrophages; mntR mutants, which constitutively express import functions, were	The two systems substitute for each other.	The two systems substitute for each other, and constitutive expression of import functions produced no phenotype in macrophages or in mice.

						transport is dependent on acidic pH (maximum between 5.5 and 6.5). Activity is competitively inhibited by Mn^{2+} and Cu^{2+}, indicating a broad spectrum of metal recognition.	based PCR to show that environmental changes in metals modulate the expression of mramp, suggesting its importance in the adaptation of the bacterium to the host environment and in metal homeostasis.		unaffected in macrophages and in mice.		
Arnold, 2020 [38]	IrtAB	irtA/irtB	Iron (Fe^{3+}-Mycobactin, Fe^{3+}-Carboxymycobactin)	Inner membrane	Influx (import) of ferric siderophores	IrtAB is an ABC exporter transporter adapted to act as an importer. Structural determinations by X-ray crystallography and cryo-EM revealed a typical ABC exporter fold, but with peculiarities in the transmembrane region that result in a partially collapsed substrate binding cavity. The cytoplasmic domain of interaction with siderophore allows the reduction of imported ferric siderophores, facilitating the release of iron. Enzymatic assays demonstrated IrtAB's preference for mycobactin over carboxymycol, indicating high specificity of the transporter.	Functional assays in membrane vesicles and in vivo mutants showed that the deletion or inactivation of IrtAB compromises bacterial growth capacity under iron limitation, highlighting the critical importance of this system for iron acquisition via siderophores in M. tuberculosis. No detailed transcriptional regulation data have been reported, but the functional role was clear in growth assays.	Transport (influx). ATP-dependent import of Fe3+-mycobactin reconstituted in proteoliposomes; cryo-EM structures resolved in several conformational states.	irtAB mutant attenuated in human macrophages and in mouse lungs.	Heme-mediated uptake; GAPDH-mediated acquisition of transferrin-bound iron.	Iron can also be obtained through the heme and transferrin routes; no inhibitor reported.
Boradia, 2014 [65]	GAPDH	Rv1436	Iron (Fe^{3+}-Human transferrin)	Cell wall surface	Iron influx via internalization of human holo-transferrin	GAPDH, traditionally a cytosolic glycolytic enzyme, has been identified on the surface of M. tuberculosis and M.	Overexpression of GAPDH in M. tuberculosis results in increased transferrin binding and greater iron uptake, both in culture and in infected cells.	Binding/delivery. Surface-exposed GAPDH binds holotransferrin with a K_D of 160 ± 24 nM (microscale thermophoresis);	GAPDH-dependent internalisation of transferrin demonstrated in	Not applicable (target not compared in Table 5).	Not applicable (target not compared in Table 5).

						smegmatis, where it acts as a human transferrin-binding protein. Mutagenesis and overexpression assays have demonstrated that GAPDH interacts directly with transferrin, promoting its internalization into the bacterial cell. The protein acts as an unconventional receptor, capable of sequestering and internalizing holo-transferrin through the mycobacterial cell wall, a mechanism unprecedented for pathogenic prokaryotes.	Functional and microscopy assays confirm that transferrin internalization occurs in a GAPDH-dependent manner, conferring an adaptive advantage in iron acquisition in intracellular environments. No data on transcriptional regulation or deletion mutants have been reported.	transferrin internalisation followed by microscopy. Transmembrane transport not measured.	infected THP-1 macrophages.		
Botella, 2011 [76]	CtpC	ctpC (Rv3270)	Zinc (Zn^{2+})	Plasma membrane	Zn^{2+} efflux (resistance to heavy metal poisoning)	CtpC is a P-type ATPase located in the plasma membrane, responsible for the export of Zn^{2+} ions from the cytoplasm to the extracellular environment. Inactivation of the ctpC gene leads to intracellular accumulation of zinc and increased sensitivity to the metal. Mutagenesis experiments have shown that ΔctpC mutants accumulate Zn^{2+} and lose the ability to export the metal, consistent with a zinc export function;	The study demonstrated by transcriptional analysis that ctpC expression is induced in response to exposure to high levels of zinc during infection in macrophages. Null mutants for ctpC showed a significant decrease in intracellular survival in human macrophages, indicating that CtpC-mediated zinc efflux is crucial for resistance to heavy metal stress imposed by the host immune system.	Transport inferred (efflux). Zn^{2+} accumulation in the ctpC mutant by ICP-MS, Mn^{2+} accumulation by atomic absorption, and metal-stimulated ATPase activity of the protein reconstituted in micelles; transmembrane flux was not measured.	No attenuation of the ctpC null mutant was detected in immunocompetent or immunodeficient animals at high inoculum.	Other zinc efflux routes.	Substrate assignment is disputed between Zn^{2+} and Mn^{2+}, transmembrane flux was not measured, and no attenuation was detected in vivo.

						transmembrane flux was not measured.				
Boudehen, 2022 [67]	CtpC (complex with PacL1)	ctpC (Rv3270)/pacL1 (Rv3269)	Zinc (Zn^{2+})	Plasma membrane (efflux platforms)	Zn^{2+} efflux (zinc toxicity resistance; assembly of efflux platforms)	CtpC is a zinc-exporting P-ATPase, and PacL1 is a chaperone protein with a transmembrane domain, a cytoplasmic region with Gln/Ala repeats, and a metal-binding motif (MBM). PacL1 associates with CtpC, forming high molecular weight complexes and dynamic foci in the membrane. Deletion of the Gln/Ala repeats of PacL1 results in the loss of CtpC and increased sensitivity to zinc. The metal-binding motif of PacL1 is only necessary at high zinc concentrations. The formation of platforms does not depend on flotillin, but depends on the Gln/Ala repeats of PacL1.	The pacL1 and ctpC genes are in the same operon, and homologues are found in other microorganisms. Deletion of pacL1 or ctpC results in high sensitivity to zinc, while PacL1 expression colocalizes and acts redundantly with other PacL orthologs. Functional assays demonstrate that PacL1 is essential for the assembly and functioning of metal export platforms, crucial for bacterial survival under zinc stress.	Transport inferred (efflux). Zn^{2+} accumulation in the ctpC mutant by ICP-MS, Mn^{2+} accumulation by atomic absorption, and metal-stimulated ATPase activity of the protein reconstituted in micelles; transmembrane flux was not measured.	No attenuation of the ctpC null mutant was detected in immunocompetent or immunodeficient animals at high inoculum.	Other zinc efflux routes.	Substrate assignment is disputed between Zn^{2+} and Mn^{2+}, transmembrane flux was not measured, and no attenuation was detected in vivo.
Buglino, 2022 [95]	Enzymes of the nrp cluster (FadD10, PPE1)/diisonitrile lipopeptides	nrp cluster (fadD10, ppe1)	Copper (Cu^{2+}, via diisonitrile chalkophores)	Secreted (extracellular copper capture and import)	Acquisition/import of copper under limitation	Diisonitrile lipopeptides (chalkophores) are synthesized by enzymes encoded by the nrp cluster. FadD10 acts as an adenylate synthase in the biosynthesis of these lipopeptides, which have diisonitrile groups capable of chelating copper with	The expression of the nrp cluster is strongly induced by copper-limiting conditions, but not by excess copper or zinc limitation. Mutants of M. tuberculosis or M. marinum deficient in fadD10, PPE1, or nonribosomal peptide synthase are sensitive to copper limitation, and this phenotype	Accessory. Chalkophore (diisonitrile lipopeptide) biosynthesis by the nrp cluster required for growth under copper limitation; the defect was restored by copper but not by Zn, Mn or Fe. No metal transport assay.	Diisonitrile lipopeptide detected in infected mouse lung; no infection phenotype was measured for the nrp mutant in this study.	Not applicable (target not compared in Table 5).	Not applicable (target not compared in Table 5).

						high affinity. Deletion of genes from the nrp cluster prevents the production of chalkophores, leading to sensitivity to copper deprivation. Reintroduction of the genes or provision of synthetic chalkophores restores resistance to copper limitation.	is reversed by genetic complementation or the addition of synthetic chalkophores, highlighting the relevance of this system for the survival of the bacillus under copper stress in the host.				
Calder, 1998 [66]	Mta72 (P-type ATPase)/Irp10	mtaA (Mta72)/irpA (Irp10)	Probably iron (Fe^{2+}/Fe^{3+}), possibly other metals	Cytoplasmic membrane (Mta72); Irp10 localization associated with response to iron limitation	Iron transport/import under limitation	Mta72 is a P-type ATPase with high homology to other metal-transporting ATPases, containing typical ATP-binding and hydrolysis domains and conserved sites associated with metal transport. Irp10 is a small protein, induced by iron limitation, suggested as a component of the transport system, possibly acting in metal recognition or transfer. The expression of Mta72 and Irp10 is regulated by the iron status in the environment.	The mtaA and irpA genes are transcribed together (in tandem), suggesting coordinated functioning in response to iron limitation. Mta72 is strongly regulated by iron: expressed under low metal availability and repressed by high concentrations. The study suggests that the Mta72/Irp10 system may be a target for interventions that compromise iron acquisition by M. tuberculosis.	Not determined. Iron-regulated envelope proteins identified and the tandem genes irp10/mtaA cloned; no transport assay was performed.	Not assessed in this study.	Not applicable (target not compared in Table 5).	Not applicable (target not compared in Table 5).
Chatterjee, 2024 [68]	Rv3270 (P-type ATPase, CtpC)	Rv3270 (ctpC)	Zinc (Zn^{2+}), Manganese (Mn^{2+})	Plasma membrane	Efflux of Zn^{2+} and Mn^{2+}, promotion of efflux of multiple antibiotics	Rv3270 is a plasma membrane P-type ATPase responsible for the export of Zn^{2+} and Mn^{2+} ions from the cytoplasm, preventing metal intoxication. The study did not assess metal	Overexpression of Rv3270 in M. smegmatis conferred greater resistance to multiple antibiotics (beta-lactams, rifampicin, etc.), correlating with increased biofilm formation. Deletion or	Transport inferred (efflux). Zn^{2+} accumulation in the ctpC mutant by ICP-MS, Mn^{2+} accumulation by atomic absorption, and metal-stimulated ATPase activity of the protein	No attenuation of the ctpC null mutant was detected in immunocompetent or immunodeficien	Other zinc efflux routes.	Substrate assignment is disputed between Zn^{2+} and Mn^{2+}, transmembrane flux was not measured, and no

						transport; it showed that Rv3270 influences antibiotic extrusion through a cotransport mechanism, possibly associated with ATP hydrolysis. Mutants overexpressing Rv3270 showed increased efflux activity, decreased intracellular accumulation of antibiotics, and a multidrug resistance phenotype.	inactivation of Rv3270 led to hypersensitivity to metals and antibiotics. Gene expression assays indicated induction of Rv3270 upon exposure to heavy metals and antibiotics, confirming its importance in cross-resistance.	reconstituted in micelles; transmembrane flux was not measured.	t animals at high inoculum.		attenuation was detected in vivo.
Chen, 2015 [90]	Copper-translocatin g P-type ATPase	Not directly specified (homolog: ctpV)	Copper (Cu^{2+})	Plasma membrane	Cu^{2+} efflux (copper stress resistance)	Copper-translocating P-type ATPase is responsible for actively pumping Cu^{2+} ions out of the cell, protecting the bacteria from copper toxicity. The study showed that copper-resistant strains have strong upregulation of this ATPase, while ABC transporters are reduced. The protein uses energy from ATP hydrolysis to transport copper across the plasma membrane.	Copper-resistant strains showed increased expression of copper ATPase and simultaneously increased resistance to isoniazid (INH). Quantitative proteomic analysis revealed that, in addition to ATPase, other cellular mechanisms were repressed (metabolic enzymes, NAD levels, FAD, etc.), resulting in a lower growth rate. There was a reduction in KatG expression, associated with cross-resistance with INH. This suggests that copper resistance may be coupled with global changes in stress response, also impacting antibiotic resistance.	Not determined. Proteomic comparison of a copper-resistant M. smegmatis strain; a copper-translocating P-type ATPase was the only transporter upregulated. The protein is not individually annotated in Tables 1-4 and the study reports no metal transport assay.	Not assessed in this study.	Not applicable (target not compared in Table 5).	Not applicable (target not compared in Table 5).
Choudhury, 2021 [50]	HupB	hupB	Iron (Fe^{3+}-Carboxymicobacti	Cell wall (cell envelope)	Fe^{3+} receptor and transporter	HupB is a 28 kDa protein located in the cell wall, whose expression is	Knockout mutants for hupB (ΔhupB) showed total deficiency in Fe^{3+}-	Binding/delivery. HupB binds Fe^{3+}-carboxymycobactin, Fe^{3+}-	Not assessed in this study.	Not applicable (target not	Not applicable (target not

			n, Fe^{3+}-Mycobactin, free Fe^{3+})		associated with siderophores	induced under iron limitation. The study shows that HupB binds to Fe^{3+}-carboxymicobactin, Fe^{3+}-mycobactin, and free Fe^{3+}. Binding assays (fluorescence, CD, SPR) and reconstitution in liposomes demonstrated that HupB functions as a ferri-carboxymicobactin receptor, promoting the internalization of iron from the siderophore into the cell. HupB also acts as a transcriptional activator of mycobactin biosynthesis.	carboxymycopactin uptake and impaired growth under iron limitation, highlighting the critical importance of the protein in the survival of M. tuberculosis under iron restriction conditions, including during macrophage infection. HupB is essential for the activation of siderophore biosynthesis and for the internalization of complex iron, comprising a fundamental step in bacterial iron homeostasis.	mycobactin and free Fe^{3+} (fluorescence, circular dichroism, SPR) and transfers iron in reconstituted liposomes; surface localisation confirmed. Transmembrane flux not measured.		compared in Table 5).	compared in Table 5).
De Miranda, 2023 [43]	FecB and FecB2 (periplasmic binding proteins)	fecB and fecB2	Iron (Fe^{3+}-siderophore, Fe^{3+}-heme)	Periplasm	Periplasmic iron transport mediated by siderophore (carboxymicobactin) and heme	FecB and FecB2 are solute binding proteins (SBPs) present in the periplasm of M. tuberculosis. The study showed that FecB has high affinity for carboxymicobactin (a siderophore), while FecB2 preferentially binds to heme and carboxymicobactin. Binding assays and crystallography revealed distinct binding sites for siderophore and heme. FecB2 exhibits dual specificity, suggesting a role in the import of iron derived from both	Deletion of fecB or fecB2 resulted in a phenotype sensitive to iron limitation, with impaired iron uptake and bacterial growth. The study used growth and iron uptake assays, confirming that both proteins are essential for adaptation to iron-limited environments, acting differently in the import of different iron sources. Gene expression of fecB and fecB2 is regulated according to the availability of iron in the environment.	Binding/delivery. Crystal structures of the periplasmic binding proteins FecB and FecB2 and binding assays with heme and Fe^{3+}-carboxymycobactin. Transport not demonstrated.	Not assessed in this study.	Not applicable (target not compared in Table 5).	Not applicable (target not compared in Table 5).

						siderophore and heme, while FecB acts more specifically in siderophore-mediated iron transport.					
Dover, 1996 [72]	Ferri-exochelin receptor (29 kDa protein)	Not specified	Iron (Fe^{3+}-Exochelin)	Cell envelope (outer/periplasmic membrane)	Receptor for Fe^{3+}-exochelin uptake/import	The 29 kDa protein has high affinity and binds directly to the ferri-exochelin complex, as demonstrated by direct binding of $^{55}Fe(III)$-exochelin in envelope assays, affinity chromatography, and co-purification. The complex is highly stable, requiring denaturation with SDS to dissociate it. A 25 kDa protein was also detected, but the 29 kDa protein is the main active receptor.	The study does not characterize gene regulation, but demonstrates that the expression and presence of the 29 kDa receptor is associated with iron deficiency conditions, suggesting induced expression in iron-limited environments to facilitate efficient uptake of this metal via exochelin.	Binding/delivery. Binding of the 29 kDa envelope protein to ferri-exochelin demonstrated by direct binding, affinity chromatography and ion-exchange separation; the molecular identity of the protein was not established.	Not assessed in this study.	Not applicable (target not compared in Table 5).	Not applicable (target not compared in Table 5).
Dragset, 2015 [56]	ESX-3 (Type VII secretion system)	ESX-3 cluster (eccB3)	Iron (Fe^{3+}-Mycobactin)	Internal membrane multiprotein complex	Required for iron acquisition via mycobactin siderophore	ESX-3 is a type VII secretion system essential for mycobacteria in mycobactin-mediated iron acquisition. The system includes several membrane components (EccC3, EccD3, etc.), responsible for protein export and transport machinery assembly. The study shows that mutations in ESX-3 cluster genes confer resistance to the PZP compound ( ), but the target of the compound is	Mutants in ESX-3 genes show impaired growth under iron limitation due to their inability to take up iron via mycobactins. PZP exacerbates this deficiency by chelating intracellular iron, inhibiting bacterial growth. The study reinforces the fundamental role of the ESX-3 system in iron homeostasis and as a potential indirect therapeutic target for mycobacteria.	Accessory. Essential for growth in a conditional mutant, with the defect reversed by iron, by zinc or by conditioned supernatant; TnSeq showed the system is required for both siderophore- and heme-mediated iron utilization.	Δesx-3 and ΔesxH highly attenuated in human macrophages; median survival of infected mice 65 days for ΔesxH versus 22 days for the wild type, largely reversed by complementation.	None identified. The system is shared by the siderophore and the heme routes.	Essentiality requires conditional approaches for validation, and the species translocated by the system is not defined; it is a secretion machine rather than a transporter.

						intracellular iron, not directly transport via ESX-3.					
Dwivedi, 2024 [99]	MctB (Copper transport protein)	mctB	Copper (Cu^{2+})	Plasma membrane	Copper efflux (homeostasis and resistance to copper stress)	The study used ADME-based virtual screening, molecular docking, and molecular dynamics to search for inhibitors of the MctB protein of M. tuberculosis. Four compounds (Anti-MCT1, Anti-MCT2, Anti-MCT3, Anti-MCT4) showed stable interactions, high affinity (binding energies between -9.2 and -9.3 kcal/mol), and adherence to Lipinski and Veber's rules. Stable binding modes between the compounds and the active site of MctB suggest potential for inhibiting transporter function.	The study did not present in vitro or in vivo validation, but suggests that MctB inhibition may compromise copper homeostasis in M. tuberculosis, making it a promising target for the development of new anti-tuberculosis therapies.	Transport inferred (efflux) for MctB (100-fold copper accumulation in the mutant) and for CtpV (csoR induction indicating higher intracellular copper); Accessory for CtpA and CtpB, whose disruption produced neither growth impairment nor copper accumulation. Transmembrane flux was not measured for any of them.	mctB mutant showed 1,000-fold and 100-fold lower burden in lungs and lymph nodes of guinea pigs; the ctpV mutant reached wild-type levels in mice and guinea pigs, with reduced lung damage and longer survival.	Four partially redundant components, in addition to cytoplasmic buffering by the metallothionein MymT.	Four partially redundant components plus cytoplasmic buffering by MymT; deletion of ctpV does not reduce bacterial burden.
Earp, 2025 [26]	MmpL4 and MmpL5 (RND-family transporters); MbtL (acyl carrier protein)	mmpL4 (Rv0450c), mmpL5 (Rv0676c), mbtL (Rv1344)	Iron (Fe^{3+}-mycobactin; siderophore export)	Plasma membrane, with a coiled-coil domain extending into the periplasm	Efflux (export) of mycobactin siderophores and of bedaquiline and clofazimine	Cryo-EM structure of monomeric MmpL4 determined at 3.0 Å. A mycobactin binding site accessible from the cytosol was identified and is also required for bedaquiline efflux. An unusual coiled-coil domain predicted to extend about 130 Å into the periplasm is essential for mycobactin and bedaquiline efflux by both	In the mycobactin toxicity assay with the M. tuberculosis ΔmmpL4/mmpL5 strain, complementation with full-length mmpL4 or mmpL5 restored wild-type growth, whereas the proton-translocation channel variants MmpL4 D272N and MmpL5 D276N were completely inactive. The MmpL4 R791A variant only partially rescued the phenotype. The	Transport (efflux) for MmpL4 and MmpL5, based on ^{14}C-labelled siderophores quantified in cells and culture filtrate; Accessory for MmpS4, MmpS5 and Rv0455c. Essential for siderophore secretion, with a strong virulence defect in mice.	mmpS4/mmpS5 double mutant showed a 10,000-fold lower bacterial burden in mouse lungs, and no infected mouse died within 180 days.	The MmpL4 and MmpL5 complexes are functionally redundant with each other, but the pair is not.	MmpL4 and MmpL5 are functionally redundant with each other, so both would have to be blocked; no inhibitor reported.

						MmpL4 and MmpL5. The mycobacterial acyl carrier protein MbtL forms a complex with MmpL4, indicating that mycobactin synthesis and export are coupled.	ΔmmpL4/mmpL5 strain is more susceptible to bedaquiline, a phenotype reversed by complementation, consistent with reports that MmpL5 overproduction in clinical strains confers bedaquiline and clofazimine resistance.				
Farhana, 2008 [37]	IrtA, IrtB, Rv2895c (siderophore exporter-importer)	irtA (Rv1348), irtB (Rv1349), rv2895c	Iron (Fe^{3+}-Mycobactin, Fe^{3+}-Carboxymycobactin; non-iron/iron-containing siderophores)	Inner membrane	IrtA exports non-iron siderophores; IrtB-Rv2895c imports iron-containing siderophores	IrtA has an N-terminal substrate-binding domain (SBD), selectively binding non-iron-containing siderophores and functioning as an exporter. IrtB, with a permease and ATPase domain, acts in conjunction with Rv2895c (high affinity for iron-containing siderophores) to form the importer of iron-rred siderophores. Reconstitution in liposomes confirmed export by IrtA and import by IrtB-Rv2895c; interaction between IrtB and Rv2895c demonstrated by pull-down.	Knockout of the irtA homolog (msmeg_6554) in M. smegmatis resulted in loss of siderophore export, restored by complementation. The system is iron-regulated and essential for homeostasis and survival of the bacillus under iron limitation.	Transport (influx). ATP-dependent import of Fe3+-mycobactin reconstituted in proteoliposomes; cryo-EM structures resolved in several conformational states.	irtAB mutant attenuated in human macrophages and in mouse lungs.	Heme-mediated uptake; GAPDH-mediated acquisition of transferrin-bound iron.	Iron can also be obtained through the heme and transferrin routes; no inhibitor reported.
Farhana, 2024 [36]	IrtA (Rv1348), IrtB-Rv2895c (Rv1349-Rv2895c), ABC	irtA (Rv1348), irtB (Rv1349), rv2895c	Iron (Fe^{3+}-Mycobactin, Fe^{3+}-Carboxymycobactin; iron-containing/non-iron-containing siderophores)	Inner membrane	IrtA exports non-iron-containing siderophores; IrtB-Rv2895c imports iron-containing siderophores	Biochemical and biophysical characterization of the domains of IrtA and IrtB-Rv2895c: the SBD domain of IrtA functions as an active monomer, while	The article reinforces that the IrtA/IrtB-Rv2895c system is essential for the survival of M. tuberculosis in murine infection models, making it a critical target for therapeutic intervention.	Transport (influx). ATP-dependent import of Fe3+-mycobactin reconstituted in proteoliposomes; cryo-EM structures resolved in several conformational states.	irtAB mutant attenuated in human macrophages and in mouse lungs.	Heme-mediated uptake; GAPDH-mediated acquisition of	Iron can also be obtained through the heme and transferrin routes; no inhibitor reported.

	transporters					Rv2895c exists in equilibrium between monomeric and dimeric forms. ATP binding induces dimerization of the ATPase domains of both, but only that of IrtA is active in isolation. Conformational changes associated with substrate binding and domain stability at different temperatures and pH levels were observed. Evolutionary analysis of the domains showed a relationship between IrtA and archaeal and eukaryotic transporters, while IrtB is specific to mycobacteria.				transferrin-bound iron.	
Goethe, 2021 [88]	ZnuABC and ZnuABC2 (zinc ABC transporters)	znuA, znuB, znuC; znuA2, znuB2, znuC2	Zinc (Zn^{2+})	Plasma membrane (ABC importer)	Zn^{2+} import (zinc homeostasis)	ZnuABC and ZnuABC2 are zinc ABC transporters; ZnuABC2 represents a new class restricted to actinobacteria. Single deletion (ΔznuABC or ΔznuABC2) does not affect growth, but double deletion ( ) causes severe defect under zinc limitation, reversed by complementation or addition of Zn^{2+}. Uptake assays with FluoZin-3AM confirmed active zinc	Double deletion resulted in increased expression of zinc deficiency marker genes. Heterologous complementation with transporters from other mycobacteria restored growth. The genes are induced under zinc limitation.	Transport (influx) for MspA and the ZnuABC subunits, based on uptake assays, or Binding/delivery; demonstrated in M. smegmatis only.	Not assessed; the evidence was obtained in M. smegmatis.	Not assessable in M. tuberculosis.	Characterized only in M. smegmatis; relevance to M. tuberculosis is not established.

						import. Zur regulates both systems by binding to promoters.					
Gonda, 2025 [34]	IrtAB (ABC transporter)	irtA, irtB	Iron (Fe^{3+}-Mycobactin, Fe^{3+}-Carboxymycobactin); Zinc (Zn^{2+}, cofactor)	Inner membrane	Import of iron-containing siderophores (mycobactin, carboxymycobactin)	Cryo-EM and DEER studies show that IrtAB alternates between inward-facing and outward-occluded conformations. There is a crevice facing the membrane, which serves as a binding site for mycobactin. Mutations in this crevice abolish import. Below the binding site is a triple histidine motif that coordinates a zinc ion. Mutation of these histidines decouples ATP hydrolysis from transport, showing that the presence of Zn^{2+} is essential for coupling between ATPase and transport function.	Mutations affecting the crevice or histidine motif result in loss of import function but maintenance of ATPase activity, highlighting the importance of the coupled mechanism. The transporter is essential for iron uptake via siderophores and is a promising molecular target for inhibition.	Transport (influx). ATP-dependent import of Fe3+-mycobactin reconstituted in proteoliposomes; cryo-EM structures resolved in several conformational states.	irtAB mutant attenuated in human macrophages and in mouse lungs.	Heme-mediated uptake; GAPDH-mediated acquisition of transferrin-bound iron.	Iron can also be obtained through the heme and transferrin routes; no inhibitor reported.
Grover, 2006 [82]	ZitA (cation diffusion facilitator, CDF)	zitA (MSMEG_0750)	Zinc (Zn^{2+})	Plasma membrane	Zn^{2+} efflux (zinc toxicity resistance)	ZitA is an integral membrane protein of the CDF family, responsible for the export of Zn^{2+} ions out of the cell. Transposon insertion mutation in the zitA gene resulted in a Zn^{2+}-hypersensitive phenotype, rescued by gene complementation. Expression of zitA is strongly induced by the presence of zinc. In silico	zitA mutants showed increased sensitivity to zinc but not to other metals, confirming specificity. Expression of zitA was highly induced in response to extracellular zinc but not to other ions. CDF genes such as zitA are relevant for survival in environments with high zinc concentrations.	Transport (influx) for MspA and the ZnuABC subunits, based on uptake assays, or Binding/delivery; demonstrated in M. smegmatis only.	Not assessed; the evidence was obtained in M. smegmatis.	Not assessable in M. tuberculosis.	Characterized only in M. smegmatis; relevance to M. tuberculosis is not established.

						analyses show that environmental and opportunistic species of Mycobacterium have more CDF genes than obligate pathogens.				
Hall, 1987 [71]	IREP III (Iron-regulated envelope protein, 29 kDa)	Not specified	Iron (Fe^{3+}-Exochelina)	Cell envelope (wall/membrane)	Potential receptor/facilitator of Fe^{3+}-exochelate uptake under iron limitation	Four iron-regulated envelope proteins were identified under iron limitation, including the 29 kDa protein (IREP III). Antibodies against IREP III inhibited 70% of exochelin-mediated iron uptake in iron-deficient cells, suggesting direct or indirect involvement in the process, although not as an exclusive receptor.	IREP III expression is induced under iron limitation. No genetic studies have been performed, but the protein is only expressed when iron is restricted, indicating iron-dependent regulation.	Binding/delivery. Four iron-regulated envelope proteins induced under iron limitation in M. smegmatis; the 29 kDa protein is absent from spheroplasts. No transport assay.	Not assessed in this study.	Not applicable (target not compared in Table 5).	Not applicable (target not compared in Table 5).
Ingale, 2021 [40]	IrtA (ABC transporter)	irtA	Iron (Fe^{3+}, via siderophores)	Internal membrane	Fe^{3+} import via siderophores	The 3D structure of IrtA was predicted by computer modeling, validated, and used for in silico screening of inhibitors. Thiosemicarbazones were identified as potential ligands, with analogue 1 showing better affinity and stability in molecular docking.	The article does not present in vivo functional validation, but highlights that IrtA is essential for iron uptake from the host and survival of the bacillus. Inhibition of IrtA is suggested as a promising strategy for the development of new anti-tuberculosis drugs.	Transport (influx). ATP-dependent import of Fe3+-mycobactin reconstituted in proteoliposomes; cryo-EM structures resolved in several conformational states.	irtAB mutant attenuated in human macrophages and in mouse lungs.	Heme-mediated uptake; GAPDH-mediated acquisition of transferrin-bound iron.	Iron can also be obtained through the heme and transferrin routes; no inhibitor reported.
Jones, 2010 [75]	Msp (porin family)	mspA, mspC, mspD	Iron (Fe^{3+}, soluble Fe^{2+}, non-siderophore)	Outer membrane	Passive diffusion of Fe^{3+}/soluble Fe^{2+} (low affinity)	Msp porins form outer membrane channels allowing diffusion of hydrophilic solutes, including ionic iron. The triple mutant	Deletion of the three porins resulted in increased expression of iron-regulated genes and increased siderophore production. Msp porins are essential for low-affinity iron	Transport (influx) for MspA and the ZnuABC subunits, based on uptake assays, or Binding/delivery;	Not assessed; the evidence was obtained in M. smegmatis.	Not assessable in M. tuberculosis.	Characterized only in M. smegmatis; relevance to M. tuberculosis is not established.

						ΔmspAΔmspCΔmspD showed a significant reduction in Fe^{3+} uptake, increased expression of iron deficiency genes, and increased siderophore production. Exochelin transport was not affected, indicating the presence of specific receptors for siderophores independent of porins.	acquisition systems in M. smegmatis.	demonstrated in M. smegmatis only.			
Jones, 2014 [30]	MmpL4/MmpS4, MmpL5/MmpS5 (transporter complexes)	mmpL4, mmpS4, mmpL5, mmpS5	Iron (Fe^{3+}-Mycobactin, Fe^{3+}-Carboxymycobactin)	Inner membrane (siderophore export/recycling)	Export and recycling of siderophores; essential for iron uptake and reuse and virulence	MmpL4/MmpS4 and MmpL5/MmpS5 form complexes involved in siderophore export. Export mutants accumulate siderophores intracellularly without recycling them, even with internalization. Experiments with radiolabeled carboxymicobactin confirmed active siderophore recycling.	Export mutants show intracellular accumulation of deferrated siderophore and bacterial death in the presence of low concentrations of siderophores. The system is essential for virulence in murine models and is a target of therapeutic interest.	Transport (efflux) for MmpL4 and MmpL5, based on ^{14}C-labelled siderophores quantified in cells and culture filtrate; Accessory for MmpS4, MmpS5 and Rv0455c. Essential for siderophore secretion, with a strong virulence defect in mice.	mmpS4/mmpS5 double mutant showed a 10,000-fold lower bacterial burden in mouse lungs, and no infected mouse died within 180 days.	The MmpL4 and MmpL5 complexes are functionally redundant with each other, but the pair is not.	MmpL4 and MmpL5 are functionally redundant with each other, so both would have to be blocked; no inhibitor reported.
Kumar, 2020 [73]	IrpA (21 kDa iron-regulated protein, MSMEG_3761, Clp protease subunit)	irpA (MSMEG_3761)	Iron (Fe^{3+}-Exochelate)	Cell wall/outer membrane	Receptor for ferri-exochelina (preferred for iron uptake in M. smegmatis)	IrpA is a 21 kDa iron-regulated protein identified as a subunit of Clp protease (MSMEG_3671), expressed under iron limitation. It acts as a receptor for ferri-exochelin, promoting efficient siderophore uptake in iron-deficient conditions. Exochelin is the	IrpA expression and exochelin production are induced under iron limitation and repressed by abundant iron. M. tuberculosis does not have this system, relying solely on carboxymicobactin/mycobactin for iron uptake.	Binding/delivery. The 21 kDa iron-regulated protein IrpA (MSMEG_3761) identified as a ferri-exochelin receptor by MALDI-TOF-MS/MS; the system is absent from M. tuberculosis. Transport not demonstrated.	Not assessed in this study.	Not applicable (target not compared in Table 5).	Not applicable (target not compared in Table 5).

						first siderophore produced and internalized via IrpA.					
León-Torres, 2015 [78]	CtpA (P-type ATPase)	ctpA	Copper (Cu^{1+})	Plasma membrane	Copper efflux/detoxification (copper homeostasis and tolerance)	CtpA is a P-type ATPase whose activity is stimulated by Cu^{1+} (not by Cu^{2+} or other metals). The apparent Km for Cu^{1+} was 0.047 μM. The study suggests that Cu^{2+} is reduced to Cu^{1+} prior to export. Optimal activity at 37 °C and pH 7.9. Heterologous expression of ctpA increases bacterial tolerance to copper.	Expression of ctpA in M. smegmatis conferred greater tolerance to copper, but not to other metals. CtpA is important for detoxification and intracellular copper homeostasis in mycobacteria.	Transport inferred (efflux) for MctB (100-fold copper accumulation in the mutant) and for CtpV (csoR induction indicating higher intracellular copper); Accessory for CtpA and CtpB, whose disruption produced neither growth impairment nor copper accumulation. Transmembrane flux was not measured for any of them.	mctB mutant showed 1,000-fold and 100-fold lower burden in lungs and lymph nodes of guinea pigs; the ctpV mutant reached wild-type levels in mice and guinea pigs, with reduced lung damage and longer survival.	Four partially redundant components, in addition to cytoplasmic buffering by the metallothionein MymT.	Four partially redundant components plus cytoplasmic buffering by MymT; deletion of ctpV does not reduce bacterial burden.
León-Torres, 2020 [81]	CtpB (P-type ATPase)	ctpB	Copper (Cu^{1+})	Plasma membrane	Transport of Cu^{1+} out of the cell; non-classical role in detoxification, homeostasis under physiological stress	CtpB expressed in M. smegmatis increases copper tolerance and has high-affinity ATPase activity (Km = 0.19 μM) and low turnover rate (Vmax = 2.29 nmol/mg·min) for Cu^{1+}. Expression is induced by hypoxia and oxidative/nitrosative stress, not by excess copper. Deletion of ctpB does not alter growth/metal accumulation under high doses of copper, indicating a role different from classical detoxification.	Transcription of ctpB increases under conditions that simulate the intraphagosomal environment (hypoxia, nitrosative/oxidative stress), not by excess copper. Function linked to physiological stress response and maintenance of copper homeostasis.	Transport inferred (efflux) for MctB (100-fold copper accumulation in the mutant) and for CtpV (csoR induction indicating higher intracellular copper); Accessory for CtpA and CtpB, whose disruption produced neither growth impairment nor copper accumulation. Transmembrane flux was not measured for any of them.	mctB mutant showed 1,000-fold and 100-fold lower burden in lungs and lymph nodes of guinea pigs; the ctpV mutant reached wild-type levels in mice and guinea pigs, with reduced lung damage and longer survival.	Four partially redundant components, in addition to cytoplasmic buffering by the metallothionein MymT.	Four partially redundant components plus cytoplasmic buffering by MymT; deletion of ctpV does not reduce bacterial burden.

Li, 2012 [89]	Rv0899 (OmpATb)	Rv0899	Zinc (Zn^{2+})	Outer membrane	Porin; channel formation and Zn^{2+} acquisition; adaptation to acidic pH	Structure resolved by NMR and EPR revealed that Rv0899 is monomeric, with two separate domains and anchored to the membrane. NMR and calorimetric titration assays showed Zn^{2+} binding to the protein. Deletion of Rv0899 impairs uptake of hydrophilic solutes and adaptation to acidic pH.	Expression of Rv0899 is associated with acid stress. Deletion compromises Zn^{2+} uptake and adaptation to hostile environments, suggesting a fundamental role in metal homeostasis.	Not determined. Association with the pathway reported without demonstration of binding or transport.	Not assessed.	Not applicable.	Neither binding nor transport has been demonstrated.
López, 2018 [98]	CtpG (P1B-type ATPase)	ctpG	Cadmium (Cd^{2+}, primary); Copper (Cu^{2+}, secondary)	Plasma membrane	Efflux/detoxification of Cd^{2+} (main) and Cu^{2+} (secondary); heavy metal tolerance	CtpG is activated in vitro by several divalent cations, but only increases tolerance to Cd^{2+} and Cu^{2+} in whole cells. Michaelis-Menten kinetics show transport efficiency (Vmax/Km) 7.4 times higher for Cd^{2+} than for Cu^{2+}. It confers tolerance to high levels of Cd^{2+} and Cu^{2+} in recombinant M. smegmatis.	CtpG confers bacterial tolerance to cadmium and copper, but not to other metals tested. The gene is potentially induced during infection, suggesting a relevant role in the response to heavy metal stress.	Transport inferred (efflux). Metal-stimulated ATPase activity in membrane vesicles and metal tolerance assays indicating preferential Cd^{2+} transport; transmembrane flux not measured. M. smegmatis.	Not assessed in this study.	Not applicable (target not compared in Table 5).	Not applicable (target not compared in Table 5).
Lopez-R, 2024 [79]	CtpA (P-type ATPase)	ctpA	Copper (Cu^{2+}, mainly Cu^{1+})	Plasma membrane	Delivery of copper as a cofactor to extracellular proteins (e.g., MmcO); response to redox stress	CtpA is not involved in direct copper detoxification at high doses, but provides copper to multicopper oxidase (MmcO) under oxidative/nitrosative stress. The absence of CtpA does not lead to intracellular copper accumulation, even under excess metal conditions. Organic substrate oxidation activity	The ctpA gene is positively regulated by low copper concentrations and in response to oxidative (H_2O_2) and nitrosative (sodium nitroprusside) stress. ΔctpA mutants show impaired growth under oxidative and nitrosative stress, but not under excess copper. The results reinforce the role of CtpA in defense against redox stress and	Transport inferred (efflux) for MctB (100-fold copper accumulation in the mutant) and for CtpV (csoR induction indicating higher intracellular copper); Accessory for CtpA and CtpB, whose disruption produced neither growth impairment nor copper accumulation. Transmembrane flux was	mctB mutant showed 1,000-fold and 100-fold lower burden in lungs and lymph nodes of guinea pigs; the ctpV mutant reached wild-type levels in mice and guinea pigs,	Four partially redundant components, in addition to cytoplasmic buffering by the metallothionein MymT.	Four partially redundant components plus cytoplasmic buffering by MymT; deletion of ctpV does not reduce bacterial burden.

						is reduced in ΔctpA mutants, indicating reduced MmcO activity.	maintenance of copper homeostasis in M. tuberculosis.	not measured for any of them.	with reduced lung damage and longer survival.		
Maharjan, 2024 [55]	MmpL4, MmpL5 (RND transporter s)	mmpL4, mmpL5	Iron (Fe^{3+}-Mycobactin, Fe^{3+}-Carboxymycocytin; siderophore export)	Inner membrane	Export of mycobactin and carboxymycoactin siderophores (iron acquisition)	Cryo-EM structures of MmpL4 (2.95 Å) and MmpL5 (3.00 Å) from M. smegmatis show typical architecture of RND transporters, with transmembrane and periplasmic domains. Proposed pathway for siderophore translocation; mutations in critical residues compromise export.	Knockout of mmpL4/mmpL5 impairs siderophore export and leads to iron acquisition deficiency. Transporters essential for survival and replication in iron-poor environments; promising targets for new drugs.	Transport (efflux) for MmpL4 and MmpL5, based on ^{14}C-labelled siderophores quantified in cells and culture filtrate; Accessory for MmpS4, MmpS5 and Rv0455c. Essential for siderophore secretion, with a strong virulence defect in mice.	mmpS4/mmpS5 double mutant showed a 10,000-fold lower bacterial burden in mouse lungs, and no infected mouse died within 180 days.	The MmpL4 and MmpL5 complexes are functionally redundant with each other, but the pair is not.	MmpL4 and MmpL5 are functionally redundant with each other, so both would have to be blocked; no inhibitor reported.
Mandal, 2021 [9]	Repertoire of ABC transporter s for metal uptake: Rv0265c (MhuP), Rv3044, Rv1857, and Rv2059 (solute-binding proteins, SBPs); Rv1858, Rv2060, and Rv2325c (TMDs);	rv0265c (mhuP), rv3044, rv1857, rv1858, rv1859, rv2059, rv2060, rv2325c, rv2326c, rv3041c, rv0908, rv1030	Iron (Fe^{3+}-siderophore, Fe^{3+}-citrate/dicitrate), Zinc (Zn^{2+}), Manganese (Mn^{2+}), Cobalt (Co^{2+}), Molybdenum (MoO_4^{2-}; also WO_4^{2-})	Periplasm/cell envelope (SBPs); plasma membrane (TMDs, NBDs and P-type ATPases)	Influx (uptake) of free and complexed metal ions, assigned by in silico prediction	Entirely in silico study. Genome mining of M. tuberculosis H37Rv identified 19 ORFs organised into seven metal uptake ABC transport systems and two P-type ATPases. Homology modelling, structure-based phylogeny and electrostatic potential analysis of the active site grouped Rv0265c and Rv3044 into a new A-II-b subcluster of SBPs that bind iron complexes; Rv1857 clustered with oxyanion-binding SBPs (D-III-a) and Rv2059 with divalent	Operon and interaction network analysis showed that, except for rv1857-rv1858-rv1859, the transporter subunits are scattered across the genome, supporting a subunit-sharing mechanism in which TMDs and NBDs are shared between distinct systems: Rv0265c would use the subunits of Rv3041c and/or DppABCD, and Rv2059-Rv2060 would lack a canonical NBD. The pair Rv2325c-Rv2326c was assigned to an ECF-type cobalt importer, with no ORF identified for the S component. No functional or transport assay was performed—all	Not determined. In silico identification and characterisation of metal-uptake ABC transporters; no binding or transport assay was performed.	Not assessed in this study.	Not applicable (target not compared in Table 5).	Not applicable (target not compared in Table 5).

	Rv1859, Rv2326c, and Rv3041c (NBDs); Rv0908 and Rv1030 (P-type ATPases)					cation-binding SBPs (A-I). The crystallographic structure of Rv0265c (PDB 4PM4) was used directly and the remaining proteins were modelled. Molecular docking indicated binding of Rv3044 to Fe^{3+}-citrate (−4.84 kcal mol^{-1}) and Fe^{3+}-dicitrate (−6.72 kcal mol^{-1}), with residues Gly162, Gln211, Arg218, Tyr220, Asn224, Phe225, Gln314, Thr315 and Glu317 lining the active site.	substrate assignments are predictive.				
Mandal, 2022 [63]	MhuP; DppABCD (ABC transporter, DppA as binding protein)	mhuP (Rv0265c), dppA, dppB	Iron (Fe^{2+}-Heme)	Periplasm (MhuP); Inner membrane (DppABCD)	Heme transfer and transport (acquisition of iron derived from heme)	Crystallographic structure of MhuP shown. MhuP binds specifically to heme; molecular docking confirms interaction with heme and DppA. Suggested mechanism of transient heme transfer between MhuP and DppA prior to entry into the DppBCD transporter.	Binding studies show specific affinity of MhuP for heme. MhuP acts as an auxiliary protein in supplying heme to DppABCD. The system contributes to iron acquisition in environments with limitations imposed by host immunity.	Binding/delivery for DppA (heme binding abolished by the Arg179 mutation); Transport inferred (influx) for DppB, DppC and DppD, based on growth and utilization phenotypes; transmembrane flux was not measured.	Impaired heme utilization in THP-1 macrophages; mouse infection not reported for the dpp mutant.	Siderophore-mediated uptake.	Transport by the permease and ATPase subunits is inferred rather than measured, and the siderophore route compensates for the loss of this pathway.
Meikle, 2023 [28]	MmpS4-MmpL4, MmpS5-MmpL5 (complexes); Rv0455c (periplasmic)	mmpL4, mmpS4, mmpL5, mmpS5, rv0455c	Iron (Fe^{3+}-Mycobactin, Fe^{3+}-Carboxymycoctin; siderophore export)	Inner membrane (MmpL4/MmpL5), periplasm (Rv0455c)	Siderophore export and drug efflux	MmpL4/MmpL5 form functional complexes with MmpS4/MmpS5 for siderophore export. Inactivation leads to toxic accumulation of siderophores and virulence deficiency. Rv0455c is essential for siderophore	Mutants lacking MmpS4-MmpL4/MmpS5-MmpL5 are more sensitive to bedaquiline, clofazimine, and rifabutin. Complementation shows that MmpS4-MmpL4 does not restore drug efflux, unlike MmpS5-MmpL5. The Rv0455c mutant is also more sensitive to the same drugs. Blocking	Transport (efflux) for MmpL4 and MmpL5, based on ^{14}C-labelled siderophores quantified in cells and culture filtrate; Accessory for MmpS4, MmpS5 and Rv0455c. Essential for siderophore secretion, with a strong virulence defect in mice.	mmpS4/mmpS5 double mutant showed a 10,000-fold lower bacterial burden in mouse lungs, and no infected mouse died within 180 days.	The MmpL4 and MmpL5 complexes are functionally redundant with each other, but the pair is not.	MmpL4 and MmpL5 are functionally redundant with each other, so both would have to be blocked; no inhibitor reported.

						secretion into the periplasm.	siderophore secretion impairs iron acquisition and potentiates antibiotics by blocking efflux at the .				
Mitra, 2019 [58]	DppABCD (DppA); PPE36, PPE62	dppA, dppB, dppC, dppD; ppe36, ppe62	Iron (Fe^{2+}-Heme, Fe^{2+}-Hemoglobin)	Inner membrane (DppABCD); outer membrane (PPE36, PPE62)	Uptake of iron derived from heme and hemoglobin	Crystallographic structure of DppA (1.27 Å) reveals site for tetrapeptide and exposed cavity for heme. Mutation of Arg179 eliminates binding to heme and prevents use of heme.	Mutants in dppA, ppe36, and ppe62 do not grow using heme or hemoglobin as an iron source. Albumin allows heme entry even without these proteins. DppABCD and PPE36/62 converge for efficient iron import from heme; their inactivation compromises survival under iron limitation.	Binding/delivery for DppA (heme binding abolished by the Arg179 mutation); Transport inferred (influx) for DppB, DppC and DppD, based on growth and utilization phenotypes; transmembrane flux was not measured.	Impaired heme utilization in THP-1 macrophages; mouse infection not reported for the dpp mutant.	Siderophore-mediated uptake.	Transport by the permease and ATPase subunits is inferred rather than measured, and the siderophore route compensates for the loss of this pathway.
Ni, 2025 [53]	IrtAB (ABC transporter, heterodime r)	irtA, irtB	Iron (Fe^{3+}-carboxymicobactin , Fe^{3+}-micobactin)	Internal membrane	Fe^{3+}-siderophore import (iron uptake from the host)	The binding mechanism between IrtAB and the N14G ligand (analogous to mycobacterial siderophores) was explored. It allows ultrasensitive detection by fluorescent probe mimicking the IrtAB substrate. It confirms IrtAB as a molecular target for recognition and possible inhibition.	N14G and N14G-Fe are specifically internalized via IrtAB, allowing rapid labeling and detection of M. smegmatis, M. bovis BCG, and M. tuberculosis H37Rv in clinical samples. Knockout of irtA/irtB eliminates internalization of probes and fluorescent labeling, confirming the functional specificity of the system. It allows detection of up to 34 CFU of M. tuberculosis in clinical samples, demonstrating the functional role of IrtAB in iron uptake and as a molecular marker.	Transport (influx). ATP-dependent import of Fe3+-mycobactin reconstituted in proteoliposomes; cryo-EM structures resolved in several conformational states.	irtAB mutant attenuated in human macrophages and in mouse lungs.	Heme-mediated uptake; GAPDH-mediated acquisition of transferrin-bound iron.	Iron can also be obtained through the heme and transferrin routes; no inhibitor reported.
Novoa-Aponte, 2012 [77]	Various P-type ATPases (12	Various (e.g., ctpA, ctpB, ctpC, ctpG, etc.; Rvxxxx)	Heavy metals (Cu^{+}/Cu^{2+}, Zn^{2+}, Co^{2+}, Ni^{2+}, Cd^{2+}, etc.), Na^{+}, K^{+}, Ca^{2+}, Mg^{2+}	Plasma membrane (predicted topology)	Active transport of essential or toxic metals; ionic homeostasis	Probabilistic profiles (HMM) and phylogenetic analyses identified 12 P-type ATPases in MTBC. Membrane topology,	There is no experimental functional validation, but the predicted high content of heavy metal transporters suggests a role in the survival of the	Not determined. Genomic and transcriptomic survey of the P-type ATPase repertoire of M. tuberculosis and its	Not assessed in this study.	Not applicable (target not compared in Table 5).	Not applicable (target not compared in Table 5).

	candidates identified)					hydrophobicity profiles, and conserved motifs (e.g., CPC, NKT, DKTGT) were determined to predict specificity for heavy metals, alkali metals, and alkaline earth metals. Absence of β subunits of H^+/K^+ and Na^+/K^+ ATPases; presence of prokaryotic β subunit of K^+ pump.	bacillus in macrophages. The results serve as a starting point for future functional studies and experimental validation.	expression under stress; no transport assay was performed.			
Ojha, 2007 [74]	Exochelin system (synthesis/transport); mycobactin ABC transporter	exochelin genes (cluster), mycobactin genes (mbt operon), ABC transporter	Iron (Fe^{3+}-Exochelin; Fe^{3+}-Mycobactin)	Exochelin: extracellular/membrane; Mycobactin: membrane/cytoplasm	Iron uptake under limitation; essential for biofilm and surface motility	Exochelin and mycobactin siderophores are small iron chelators exported into the environment. Uptake involves receptor proteins and ABC transporters. Exochelin genes are highly induced in biofilm.	Exochelin mutants show defective biofilm formation under low iron conditions, but grow normally in planktonic culture. Mycobactin genes are induced, but mutants show no relevant defect in biofilm under iron limitation. Iron acquisition via exochelin is essential for transition to biofilm, suggesting a regulatory role for iron.	Not determined. Deletion of the fxuABC exochelin-uptake operon reduced biofilm biomass to 9.5% of the control in the standard biofilm medium (which requires a 2 mM iron supplement), without affecting planktonic growth. M. smegmatis.	Not assessed in this study.	Not applicable (target not compared in Table 5).	Not applicable (target not compared in Table 5).
Owens, 2012 [62]	Rv0203 (secreted heme-binding protein)	Rv0203	Iron (Fe^{2+}-Heme)	Extracellular (secreted)	Heme uptake/binding (acquisition of iron derived from heme)	Heme coordination environment depends on state/tag: His-tag form exhibits bis-His coordination (low-spin), native form exhibits high-spin, similar to oxygen-bound penta-coordinated proteins. Mutagenesis reveals Tyr59, His63, His89 residues essential for heme binding. Affinity and kinetic measurements show similar binding rates	Mutagenesis confirms the importance of Tyr59, His63, and His89 for heme binding. Rv0203 is a secreted protein essential for iron acquisition via heme in M. tuberculosis. Structural differences between recombinant and native forms may affect physiological function.	Binding/delivery. Heme ligation properties of the secreted heme-binding protein Rv0203 characterised spectroscopically. Transport not demonstrated.	Not assessed in this study.	Not applicable (target not compared in Table 5).	Not applicable (target not compared in Table 5).

						but different dissociation rates.					
Owens, 2013 [60]	Rv0203 (secreted); MmpL3, MmpL11 (membrane)	rv0203, mmpL3, mmpL11	Iron (Fe^{2+}-Heme)	Rv0203: extracellular; MmpL3/MmpL11: inner membrane (extracellular domains)	Rv0203 transfers heme to extracellular domains of MmpL3/MmpL11, which import heme into the cytosol	E1 domains of MmpL3 and MmpL11 have high affinity for heme (characterization by absorption spectroscopy/EPR). Rv0203 efficiently transfers heme to these domains.	Mutants in mmpL11 show deficiency in heme acquisition. MmpL3/MmpL11 are necessary for efficient import of heme into the cytosol. Mechanism represents a potential target for inhibition of iron uptake in M. tuberculosis.	Binding/delivery for Rv0203 and MmpL3; Accessory for MmpL11. Heme transfer from Rv0203 to the extracellular domains of MmpL3 and MmpL11 demonstrated in vitro. Transmembrane transport not demonstrated.	Not assessed in this study.	Not applicable (target not compared in Table 5).	Not applicable (target not compared in Table 5).
Padilla-Benavides, 2013 [83]	CtpC (P1B-type ATPase)	ctpC	Manganese (Mn^{2+}), Zn^{2+} resistance (export)	Plasma membrane	Mn^{2+} transport, assembly of secreted metalloproteins, resistance to Zn^{2+} toxicity	CtpC has Mn^{2+}-dependent ATPase activity. Mutations in ctpC alter Mn^{2+} homeostasis, increase sensitivity to redox stress, and decrease Mn-superoxide dismutase activity. It is responsible for the correct metallization of Mn-dependent secreted proteins.	Mutants in ctpC are more sensitive to oxidative stress and have deficient assembly of secreted metalloproteins. Mutants lose virulence in a murine model. CtpC also protects against Zn^{2+} toxicity by exporting the metal out of the cytoplasm.	Transport inferred (efflux). Zn^{2+} accumulation in the ctpC mutant by ICP-MS, Mn^{2+} accumulation by atomic absorption, and metal-stimulated ATPase activity of the protein reconstituted in micelles; transmembrane flux was not measured.	No attenuation of the ctpC null mutant was detected in immunocompetent or immunodeficient animals at high inoculum.	Other zinc efflux routes.	Substrate assignment is disputed between Zn^{2+} and Mn^{2+}, transmembrane flux was not measured, and no attenuation was detected in vivo.
Pandey, 2015 [94]	MntR (transcriptional regulator), MntH (importer), MntABCD (ABC transporter)	mntR (Rv2788), mntH, mntA, mntB, mntC, mntD	Manganese (Mn^{2+})	MntR: cytoplasmic; MntH/MntABCD: inner membrane	Mn^{2+} import (MntH/MntABCD); repression of expression of these transporters (MntR)	MntR is a DtxR/MntR regulator that represses the expression of manganese import genes in response to Mn^{2+}. MntH is an importer of the Nramp family; MntABCD is an ABC transporter for Mn^{2+}.	Repression of manganese transport by MntR is essential under high Mn^{2+} availability. MntR is dispensable during infection. MntH/MntABCD are indispensable for replication in macrophages. Mutants in mntH/mntABCD have severely impaired growth in macrophages.	Transport (influx) for MntH ($^{65}Zn^{2+}$/$^{55}Fe^{2+}$ uptake in Xenopus oocytes); Transport inferred for MntABCD (manganese content by ICP-MS). The two systems were identified as the main Mn^{2+} importers of Mtb.	Inactivation of MntABCD partially attenuated Mtb in macrophages; mntR mutants, which constitutively express import functions, were unaffected in macrophages and in mice.	The two systems substitute for each other.	The two systems substitute for each other, and constitutive expression of import functions produced no phenotype in macrophages or in mice.

Patel, 2016 [70]	CtpD, CtpJ (P1B4-type ATPases)	ctpD, ctpJ	Iron (Fe^{2+}), Cobalt (Co^{2+})	Plasma membrane	Efflux and homeostasis of Fe^{2+} and Co^{2+}; protection against ionic toxicity	CtpD/CtpJ activated by Fe^{2+} and Co^{2+}, bind 1 eq of each at the transport site. CtpD has higher affinity/activity for Fe^{2+} than CtpJ. Mutagenesis identifies Ser, His, Glu, and Cys as invariant at the binding site; Cys mutation reduces affinity for Fe^{2+}.	ctpJ mutant (M. smegmatis) affects Fe^{2+}/Co^{2+} homeostasis; in M. tuberculosis, ctpJ mutant is more sensitive to Co^{2+}, ctpD mutant to Fe^{2+}. Differential expression/alternative function ensure survival in variable Fe^{2+}/Co^{2+} environments.	Transport inferred (efflux). Metal-stimulated ATPase activity and metal content in mutants; CtpD binds Fe^{2+} with a K_D of 0.12 ± 0.04 μM versus 2.41 ± 0.27 μM for CtpJ. Transmembrane flux not measured.	Not assessed in this study.	Not applicable (target not compared in Table 5).	Not applicable (target not compared in Table 5).
Quezada, 2019 [97]	CorA (Mg^{2+}/Co^{2+} ion channel)	corA	Magnesium (Mg^{2+}), Cobalt (Co^{2+})	Plasma membrane (inflow channel)	Mg^{2+}/Co^{2+} influx/homeostasis; maintenance of intracellular concentration	CorA is a predicted ion channel specific for Mg^{2+}/Co^{2+}. Resistance mutations map to transmembrane and regulatory regions, altering affinity/transport. Predicted structure of five subunits with central pore.	Bactericidal compounds deplete intracellular Mg^{2+} and select resistant corA mutants. Excess extracellular Mg^{2+} reverses the bactericidal effect. Mg^{2+} homeostasis is an essential vulnerability of the bacillus.	Accessory. Resistance mutations in corA were selected by 4-hydroxyquinolines and a barbituric acid derivative; the authors concluded that CorA is not the target of these compounds but mitigates their bactericidal effect through a compensatory influx of Mg^{2+}. No transport assay was performed.	Not assessed; the compounds were evaluated only in vitro.	Other divalent cation routes.	The compounds that select corA mutations do not act on CorA; no transport assay and no infection model are available.
Raimunda, 2012 [85]	CtpD (P1B4-type ATPase)	ctpD	Cobalt (Co^{2+}), Nickel (Ni^{2+}), residual activity for Zinc (Zn^{2+})	Plasma membrane	Co^{2+} and Ni^{2+} efflux/homeostasis; detoxification/protection against oxidative stress	CtpD exhibits Co^{2+}-, Ni^{2+}-, and Zn^{2+}-dependent ATPase activity (20% of maximum). Purified protein binds 1 eq of Co^{2+}, Ni^{2+}, or Zn^{2+} at the transport site.	The ΔctpD mutant (M. smegmatis) is hypersensitive to Co^{2+} and Ni^{2+}, accumulating these metals in the cytoplasm. CtpD transcription is induced by Co^{2+} and oxidative stress. The ortholog in M. tuberculosis is thought to have a similar function in cellular stress resistance via metal regulation.	Transport inferred (efflux). Co^{2+}/Ni^{2+} content in M. smegmatis mutants and metal-stimulated ATPase activity; transmembrane flux not measured.	Not assessed in this study.	Not applicable (target not compared in Table 5).	Not applicable (target not compared in Table 5).
Raimunda, 2014 [84]	CtpD (Rv1469), CtpJ	ctpD, ctpJ	Cobalt (Co^{2+}), Nickel (Ni^{2+})	Plasma membrane	Co^{2+} and Ni^{2+} efflux/homeostasis; distinct functions	Both activated by Co^{2+}/Ni^{2+}; both required for efflux. CtpJ: high turnover rate,	CtpJ mutation leads to cytoplasmic accumulation of Co^{2+}. CtpD mutation reduces	Transport inferred (efflux). Co^{2+} content and metal sensitivity in mutants and	Competitive infection of female C57BL/6J	Not applicable (target not	Not applicable (target not

	(Rv3743) — P1B4 ATPases				in virulence and redox response	induced by Co^{2+}, mutant accumulates Co^{2+}. CtpD: low turnover rate, induced by redox stress, co-transcribed with thioredoxin/enoyl-CoA hydratase genes. TrxA binds Co^{2+}/Ni^{2+}.	fitness in mice. CtpD essential for virulence/redox maintenance in vivo; CtpJ does not affect growth in vivo.	Fe^{2+}-stimulated ATPase activity; a ΔctpD ΔzitA double mutant showed that CtpD does not contribute to zinc efflux. Transmembrane flux not measured.	mice: only ctpD deletion significantly reduced bacterial fitness in the lungs, whereas ctpJ deletion did not affect virulence.	compared in Table 5).	compared in Table 5).
Rodriguez, 2006 [35]	IrtAB (ABC transporter)	irtA, irtB	Iron (Fe^{3+}-Carboxymycobactin)	Inner membrane (ABC transporter)	Import of Fe^{3+}-carboxymicobactin; essential for growth under iron limitation and virulence	IrtAB similar to the YbtPQ system of Yersinia pestis (ABC transporter). Does not participate in siderophore synthesis/secretion, but necessary for efficient use of Fe^{3+}-carboxymicobactin. Located in a cluster dedicated to siderophore synthesis and transport, regulated by IdeR.	Inactivation of irtAB reduces the ability to grow under iron limitation and survive in macrophages/lungs of mice. Does not affect siderophore synthesis/secretion, only internalization/utilization. IrtAB cluster essential for virulence/host adaptation.	Transport (influx). ATP-dependent import of Fe3+-mycobactin reconstituted in proteoliposomes; cryo-EM structures resolved in several conformational states.	irtAB mutant attenuated in human macrophages and in mouse lungs.	Heme-mediated uptake; GAPDH-mediated acquisition of transferrin-bound iron.	Iron can also be obtained through the heme and transferrin routes; no inhibitor reported.
Ryndak, 2010 [54]	IrtAB (ABC transporter; FAD-binding domain in IrtA)	irtA, irtB	Iron (Fe^{3+}-Exomycobactins; Fe^{3+}-Carboxymycobactin)	Inner membrane	Fe^{3+}-siderophore import; intracellular iron assimilation	IrtA has an N-terminal FAD-binding domain, essential for iron acquisition and coupling transport to imported iron metabolism. IrtAB regulated by iron availability (IdeR).	IrtA/irtB mutants do not grow under iron limitation even in the presence of exogenous siderophores. Mutation in the FAD-binding domain abolishes iron import/assimilation. IrtAB is essential for replication in macrophages and virulence in mice. FAD domain connects transport to iron use/metabolism.	Transport (influx). ATP-dependent import of Fe3+-mycobactin reconstituted in proteoliposomes; cryo-EM structures resolved in several conformational states.	irtAB mutant attenuated in human macrophages and in mouse lungs.	Heme-mediated uptake; GAPDH-mediated acquisition of transferrin-bound iron.	Iron can also be obtained through the heme and transferrin routes; no inhibitor reported.
Sandhu, 2017 [31]	MmpL5-MmpS5 (membrane complex)	mmpL5, mmpS5	Iron (Fe^{3+}-Mycobactin, Fe^{3+}-Carboxymycobactin)	Inner/periplasmic membrane	Export of mycobactin/carboxymycoctin to the	Docking/molecular dynamics show mycobactin in the cytoplasmic and	Interactions of MmpL5 with proteins associated with iron acquisition and mycobactin biosynthesis suggested in	Transport (efflux) for MmpL4 and MmpL5, based on ^{14}C-labelled siderophores quantified in	mmpS4/mmpS5 double mutant showed a 10,000-fold	The MmpL4 and MmpL5 complexes are functionally	MmpL4 and MmpL5 are functionally redundant with

					extracellular/periplasmic space	periplasmic sites of MmpL5. MmpL5 transports mycobactin from the cytoplasm to the periplasm via the central channel. MmpS5 facilitates periplasmic release, increasing transport efficiency.	protein interaction network. Exporter is a potential target for drug development.	cells and culture filtrate; Accessory for MmpS4, MmpS5 and Rv0455c. Essential for siderophore secretion, with a strong virulence defect in mice.	lower bacterial burden in mouse lungs, and no infected mouse died within 180 days.	redundant with each other, but the pair is not.	each other, so both would have to be blocked; no inhibitor reported.
Sankey, 2023 [64]	ESX-4 (T7SS); Rv0125, Rv1085c; PPEs (pore proteins)	esx-4 locus (esxU, esxT), rv0125, rv1085c, PPE genes	Iron (Fe^{2+}-Heme)	ESX-4: inner membrane/secretion system; Rv0125/Rv1085c: secreted; PPEs: membrane (pore-forming)	Import of extracellular heme via secretion and pore formation in the membrane	ESX-4 controls secretion of Rv0125 and Rv1085c proteins, both essential for heme uptake. Mtb PPEs are pore proteins, experimentally demonstrated as membrane channels.	Deletion of ESX-4, Rv0125, or Rv1085c reduces/abolishes heme uptake. Deletion of the alternative sigma factor SigM reduces expression of core genes of heme uptake pathways. PPEs identified as membrane channels functionally associated with heme uptake.	Accessory for EccC4, PepA (Rv0125) and Rv1085c; Binding/delivery for PPE36 and PPE64. An intracellular heme biosensor showed reduced cytoplasmic heme in the ΔeccC4 mutant; PPE64 binds heme and forms high-conductance (~11 nS) channels in artificial lipid bilayers. Transmembrane heme transport not demonstrated.	Not assessed in this study.	Not applicable (target not compared in Table 5).	Not applicable (target not compared in Table 5).
Santhanagopalan, 2012 [42]	Rv2895c (homologous to ViuB of V. cholerae)	rv2895c	Iron (Fe^{3+}-siderophores: mycobactin/carboxymycocytin)	Predicted cytoplasmic (intracellular stage of siderophore iron acquisition/utilization)	Predicted to be involved in the assimilation/utilization of siderophore-transported iron	Homologous to V. cholerae ViuB protein (Fe^{3+} reductase in siderophores). Sequence and in vitro studies suggest a role in intracellular iron processing.	Deletion of rv2895c does not affect growth under iron limitation or in human macrophages. Rv2895c is not essential for iron uptake/utilization under normal laboratory conditions or infection. Contrary to previous predictions, showing that the protein is not critical for adaptation to iron limitation in vivo.	Binding/delivery (in vitro; dispensable in vivo). Rv2895c (ViuB homologue) was dispensable for replication under iron limitation; no transport assay.	The ST212 mutant replicated like the wild type in THP-1 derived macrophages, indicating that Rv2895c is not required to overcome iron limitation in host cells.	Not applicable (target not compared in Table 5).	Not applicable (target not compared in Table 5).

Serafini, 2009 [57]	ESX-3 type VII secretion system (11-gene cluster)	esx-3 gene cluster (Rv0282–Rv0292)	Iron (Fe^{3+}) and Zinc (Zn^{2+})	Cell envelope (secretion apparatus)	Secretion system required for optimal iron and zinc uptake; no direct metal transport demonstrated	No structural data. A conditional mutant was constructed by replacing the two native promoters upstream of Rv0282, which are under Zur and IdeR control, with the Pptr promoter regulated by the Pip repressor, so that transcription of the whole ESX-3 cluster can be downregulated by anhydrotetracycline.	Downregulation of the ESX-3 cluster arrested growth, showing that this type VII secretion system is essential for M. tuberculosis. The growth defect was complemented by ferric ammonium citrate, by $ZnSO_4$ or by conditioned supernatant from a wild-type culture, indicating that ESX-3 secretes a soluble factor required for optimal iron and zinc uptake. Transcription of the cluster is driven by two promoters regulated by the zinc uptake repressor Zur and the iron-dependent repressor IdeR.	Accessory. Essential for growth in a conditional mutant, with the defect reversed by iron, by zinc or by conditioned supernatant; TnSeq showed the system is required for both siderophore- and heme-mediated iron utilization.	Δesx-3 and ΔesxH highly attenuated in human macrophages; median survival of infected mice 65 days for ΔesxH versus 22 days for the wild type, largely reversed by complementation.	None identified. The system is shared by the siderophore and the heme routes.	Essentiality requires conditional approaches for validation, and the species translocated by the system is not defined; it is a secretion machine rather than a transporter.
Serafini, 2013 [47]	ESX-3 system (T7SS—Type VII Secretion System)	esx-3 locus (eccC3, esxG, esxH, etc.)	Iron (Fe^{3+}-siderophores: carboximycopactin /mycopactin), Zinc (Zn^{2+})	Transmembrane secretion complex (inner membrane, bacterial envelope)	Homeostasis/import of iron and zinc; essential for viability and adaptation to limitation/non-limiting metals	ESX-3 is a type VII secretion system composed of multiple subunits. Conditional deletion compromises the expression/functioning of siderophore transporters and Fe uptake. In M. smegmatis, it is essential for Fe limitation; in Mtb, it is essential for Fe and Zn (d function in general homeostasis).	ESX-3-depleted mutants have severely impaired growth under Fe and Zn limitation and under non-limiting conditions. In M. smegmatis, there is only a phenotype under Fe limitation. ESX-3 affects the expression of Fe/Zn metabolism genes (shown by gene expression/phenotype).	Accessory. Essential for growth in a conditional mutant, with the defect reversed by iron, by zinc or by conditioned supernatant; TnSeq showed the system is required for both siderophore- and heme-mediated iron utilization.	Δesx-3 and ΔesxH highly attenuated in human macrophages; median survival of infected mice 65 days for ΔesxH versus 22 days for the wild type, largely reversed by complementation.	None identified. The system is shared by the siderophore and the heme routes.	Essentiality requires conditional approaches for validation, and the species translocated by the system is not defined; it is a secretion machine rather than a transporter.
Shankar, 2024 [52]	FecA (putative TonB-dependent	fecA homolog, accession SGD04621.1 (M.	Iron (Fe^{3+}-siderophore)	Outer membrane	Influx (uptake) of ferric-siderophore complexes, assigned by in silico prediction	Entirely in silico study. The putative FecA sequence was retrieved from the NCBI protein database using the E. coli FecA	The sequence analysed comes from a recently updated M. tuberculosis genome assembly (strain 2926STDY5723586) rather than from the H37Rv	Not determined. In silico characterisation of a putative TonB-dependent transporter (FecA homologue, strain	Not assessed in this study.	Not applicable (target not compared in Table 5).	Not applicable (target not compared in Table 5).

	transporter, TBDT)	tuberculosis strain 2926STDY57 23586 genome assembly)				sequence as query (43.95% identity, 95% query coverage) and modelled by homology. The model adopts the classic barrel-and-plug architecture, with a 22-stranded transmembrane β-barrel enclosing a 124-residue plug domain connected by a 10-residue linker. Molecular docking followed by 100 ns molecular dynamics simulations placed the initial binding of the ferric siderophore at an extracellular pocket formed by the plug residues Gln136, Leu137 and Ser138 and the barrel residues Glu291, Asn292, Arg294, Ser317, Lys318, Lys319, His345 and Ala348, with the conserved residues Gly95 and Tyr132 also contributing. Transport follows an induced-fit model, with ligand binding eliciting shifts of 2–10 Å in the barrel and plug regions that widen the tunnel.	reference annotation. No experimental, functional or transport assay was performed; all mechanistic assignments are computational.	2926STDY5723586); no binding or transport assay.			
Shey-Njila, 2022 [80]	CtpB (P1B-type ATPase)	ctpB	Copper (Cu^{+}/Cu^{2+})	Plasma membrane	Copper transport; facilitates growth under Cu limitation;	P-type ATPase, with ATP-binding motifs and typical metal transport domains. N-terminal metal-binding	ΔctpB mutant grows normally in copper-rich media, but growth is impaired under copper limitation.	Transport inferred (efflux) for MctB (100-fold copper accumulation in the mutant) and for CtpV	mctB mutant showed 1,000-fold and 100-fold lower	Four partially redundant components, in addition to	Four partially redundant components plus cytoplasmic

					homeostasis and protection	domain, essential for response to copper limitation. Required for growth under low Cu availability.	Complementation restores phenotype. Expression induced by limitation, but not by excess copper. ΔctpB is no longer sensitive to copper toxicity, indicating a specific function in acquisition under scarcity.	(csoR induction indicating higher intracellular copper); Accessory for CtpA and CtpB, whose disruption produced neither growth impairment nor copper accumulation. Transmembrane flux was not measured for any of them.	burden in lungs and lymph nodes of guinea pigs; the ctpV mutant reached wild-type levels in mice and guinea pigs, with reduced lung damage and longer survival.	cytoplasmic buffering by the metallothionein MymT.	buffering by MymT; deletion of ctpV does not reduce bacterial burden.
Siegrist, 2009 [45]	ESX-3 system (T7SS—Type VII Secretion System)	esx-3 locus	Iron (Fe^{3+}-Mycobactin)	Multicomplex system crossing envelope/membrane (secretion/transport)	Essential for iron internalization/assimilation via mycobactin; necessary for growth/virulence	ESX-3 is a type VII secretion system, conserved in mycobacteria, required for the utilization of iron bound to mycobactin. It does not affect siderophore synthesis, only its utilization.	Δesx-3 mutants synthesize mycobactin but do not utilize iron captured by it, leading to severe growth and infection defects. Mycobacteria require ESX-3 to internalize/utilize iron from the siderophore. ESX-3 is a key target for interference in iron homeostasis.	Accessory. Essential for growth in a conditional mutant, with the defect reversed by iron, by zinc or by conditioned supernatant; TnSeq showed the system is required for both siderophore- and heme-mediated iron utilization.	Δesx-3 and ΔesxH highly attenuated in human macrophages; median survival of infected mice 65 days for ΔesxH versus 22 days for the wild type, largely reversed by complementation.	None identified. The system is shared by the siderophore and the heme routes.	Essentiality requires conditional approaches for validation, and the species translocated by the system is not defined; it is a secretion machine rather than a transporter.
Siegrist, 2014 [46]	ESX-3 system (EsxG, EsxH, EccC3, MycP3, etc.)	esx-3 locus (esxG, esxH, eccC3, mycP3, etc.)	Iron (Fe^{3+}-Mycobactin)	Multicomplex transmembrane system (bacterial membrane/envelope)	Iron acquisition via mycobactin; export of extracellular EsxH/EsxG	All components tested (except partially MycP3) are essential for growth under iron limitation and EsxH/EsxG export. MycP3 partially dispensable: mutant allows sufficient residual export for some growth. Minimum levels of	esx-3 mutants (including ΔmycP3) do not grow under iron limitation; complementation restores growth at different levels. Traces of extracellular EsxH/EsxG are sufficient for function rescue. Study reveals complexity in the relationship between structure/secretion	Accessory. Essential for growth in a conditional mutant, with the defect reversed by iron, by zinc or by conditioned supernatant; TnSeq showed the system is required for both siderophore- and heme-mediated iron utilization.	Δesx-3 and ΔesxH highly attenuated in human macrophages; median survival of infected mice 65 days for ΔesxH versus 22 days for the wild	None identified. The system is shared by the siderophore and the heme routes.	Essentiality requires conditional approaches for validation, and the species translocated by the system is not defined; it is a secretion machine

					extracellular EsxH/EsxG restore iron acquisition.	and function of ESX-3 in iron homeostasis.		type, largely reversed by complementation.		rather than a transporter.
Speer, 2013 [87]	Porins of the MspA family (outer membrane channel)	mspA	Copper (Cu^{2+}, Cu^{+})	Outer membrane (porin channel)	Mediates copper ion entry through the outer membrane	MspA is a high-conductance porin channel. Deletion of mspA reduces growth with trace amounts of copper, but increases tolerance to high copper. Heterologous expression in Mtb reduces growth in the presence of copper. Polyamines (porin inhibitors) increase copper tolerance.	Deletion of mspA increases copper resistance but impairs growth under limitation. Expression of mspA in Mtb increases copper sensitivity. The outer membrane is the primary barrier to copper, and porins regulate the metal's access to the cell interior.	Transport (influx) for MspA and the ZnuABC subunits, based on uptake assays, or Binding/delivery; demonstrated in M. smegmatis only.	Not assessed; the evidence was obtained in M. smegmatis.	Not assessable in M. tuberculosis.	Characterized only in M. smegmatis; relevance to M. tuberculosis is not established.
Sun, 2022 [41]	IrtAB (atypical ABC transporter)	irtA, irtB	Iron (Fe^{3+}-siderophores)	Inner membrane (transmembrane)	Active import (ATP-dependent) of iron-loaded siderophores, essential for viability and replication	Typical structure of ABC exporters, but functions as an importer. Unexpected occluded conformation, hydrophilic internal cavity (~4600 Å^3). NBDs form head-to-tail dimers with ATP. Asymmetric allosteric mechanism: rigid rotation of NBDs and concerted movement of the helical region of the TMD.	IrtAB essential for Mtb growth and viability due to unique ability to internalize iron-loaded siderophores. Detailed molecular mechanism provides basis for understanding ATP-mediated transport, suggesting potential therapeutic target.	Transport (influx). ATP-dependent import of Fe3+-mycobactin reconstituted in proteoliposomes; cryo-EM structures resolved in several conformational states.	irtAB mutant attenuated in human macrophages and in mouse lungs.	Heme-mediated uptake; GAPDH-mediated acquisition of transferrin-bound iron.	Iron can also be obtained through the heme and transferrin routes; no inhibitor reported.
Sun, 2024 [51]	Rv1258c (major facilitator superfamily, MFS, efflux pump); ESX-3	rv1258c; esx-3 cluster (eccA3, eccB3, eccC3, eccD3, eccE3, ppe4)	Iron (indirectly, through regulation of ESX-3)	Plasma membrane	Drug efflux pump that regulates ESX-3-mediated iron uptake; no direct metal transport demonstrated	No experimental structure. The Protein–Protein Affinity Predictor (PPA-Pred2) and AlphaFold 2 predicted direct interactions of Rv1258c with PPE4 and EccD3 and a weak interaction with	qRT-PCR of the H37RvΔRv1258c mutant showed altered expression of eccA3, eccB3, eccC3, eccD3 and eccE3 relative to wild-type H37Rv. ICP-MS measurements revealed a higher iron concentration in the culture	Not determined. Association with the pathway reported without demonstration of binding or transport.	Not assessed.	Not applicable.	Neither binding nor transport has been demonstrated.

	components PPE4, EccB3 and EccD3					EccB3; these interactions were subsequently confirmed with a bacterial two-hybrid expression system.	medium and a lower iron concentration in the lysed bacilli of the mutant, indicating impaired iron uptake. Deletion of rv1258c also raised the intracellular H^+ potential and the NAD^+/NADH ratio, resulting in oxidative stress.				
Tufariello, 2016 [48]	ESX-3 system (especially EsxG and EsxH)	esxG, esxH, esx-3 operon	Iron (Fe^{3+}-Mycobactin/Fe^{3+}-Carboxymycobactin)	Transmembrane membrane complex (bacterial membrane/envelope), EsxG/EsxH extracellular	Efficient iron uptake via siderophores, essential for growth and virulence	EsxG and EsxH are effector proteins secreted by ESX-3, essential for iron uptake via mycobactin/carboxymycoctin. Their absence severely compromises iron acquisition.	Δesx-3 mutants with defective internalization of siderophore-bound iron show a drastic reduction in their ability to infect macrophages and grow in iron-poor media. Supplementation with iron complexes allows mutants to recover, confirming the primary defect in iron acquisition. EsxG/EsxH play an additional role in virulence.	Accessory. Essential for growth in a conditional mutant, with the defect reversed by iron, by zinc or by conditioned supernatant; TnSeq showed the system is required for both siderophore- and heme-mediated iron utilization.	Δesx-3 and ΔesxH highly attenuated in human macrophages; median survival of infected mice 65 days for ΔesxH versus 22 days for the wild type, largely reversed by complementation.	None identified. The system is shared by the siderophore and the heme routes.	Essentiality requires conditional approaches for validation, and the species translocated by the system is not defined; it is a secretion machine rather than a transporter.
Tullius, 2011 [61]	Heme acquisition system (Rv0202c, Rv0203, Rv0207c)	rv0202c, rv0203, rv0207c	Iron (Fe^{2+}-Heme)	Internal membrane (Rv0202c, Rv0207c); extracellular/periplasmic secreted protein (Rv0203)	Iron uptake via free heme and hemoglobin	Rv0203 (heme carrier) with a unique crystallographic structure, distinct from other known heme-binding proteins. Direct interaction with heme indicating a specific role in metal uptake.	Deletion of the Rv0202c-Rv0207c locus severely compromises the use of heme as an iron source. Specific system of mycobacteria with no functional homologues in other pathogens, confirming functional and pathogenic relevance, potential specific therapeutic target against Mtb.	Binding/delivery for Rv0203; Accessory for MmpL11; Not determined for Rv0204c, Rv0205 and Rv0207c. Heme uptake impaired in deletion mutants of the Rv0202c-Rv0207c region; heme transfer from Rv0203 to MmpL3/MmpL11 proposed. Transmembrane transport not demonstrated.	The ΔmmpL11 mutant established infection normally in a murine low-dose aerosol model but was significantly less lethal than the wild type.	Not applicable (target not compared in Table 5).	Not applicable (target not compared in Table 5).

Tullius, 2019 [59]	PPE37 (PPE family)	ppe37	Iron (Fe^{2+}-Heme)	Cell surface/outer membrane (predicted)	Essential iron acquisition via heme (HIA), growth in low heme concentration	Specific deletion of nine amino acids (aa 31-39) near the N-terminal in PPE37 of BCG causes severe defect in iron acquisition via heme. N-terminal region crucial for correct function.	Deletion of ppe37 in Mtb results in severe attenuation in iron acquisition via heme, requiring 200x more heme for parental-like growth. Genetic complementation with ppe37 restores iron acquisition capacity via heme. High genetic variability in ppe37 among Mtb strains, frequent mutations affect iron acquisition function.	Accessory. ppe37 shown to be essential for heme-iron acquisition; more than 60% of complete M. tuberculosis genomes carry defective ppe37 variants and BCG lacks a functional copy. Mechanism and transport not determined.	Not assessed in this study.	Not applicable (target not compared in Table 5).	Not applicable (target not compared in Table 5).
Wang, 2020 [86]	Porin A (MspA)	mspA	Ca^{2+}, Mn^{2+}, Co^{2+}, Ni^{2+}, Zn^{2+}, Pb^{2+}, Cd^{2+}	Outer membrane	Outer membrane channel for passage/interaction with metal ions	Cone structure superior to α-hemolysin for direct interaction with metal ions. Significantly greater amplitude of events (up to 10 pA), facilitating structural/dynamic analysis and analysis.	pH modulation (6.8 to 8.0) controls the binding time of ions to MspA, revealing potential as a tool for analyzing ions and small molecules.	Transport (influx) for MspA and the ZnuABC subunits, based on uptake assays, or Binding/delivery; demonstrated in M. smegmatis only.	Not assessed; the evidence was obtained in M. smegmatis.	Not assessable in M. tuberculosis.	Characterized only in M. smegmatis; relevance to M. tuberculosis is not established.
Ward, 2008 [92]	CtpV (copper efflux ATPase)	ctpV (cso operon)	Copper (Cu^{2+}, Cu^{+})	Plasma membrane	Active copper efflux, protection against toxicity	P-type ATPase with ATP- and metal-binding domains, typical structure of these ATPases for copper removal.	ctpV gene specifically induced by copper, not by other metals. Microarray revealed 30 copper-responsive genes. Experimental confirmation of the essential function of ctpV in copper homeostasis in Mtb.	Transport inferred (efflux) for MctB (100-fold copper accumulation in the mutant) and for CtpV (csoR induction indicating higher intracellular copper); Accessory for CtpA and CtpB, whose disruption produced neither growth impairment nor copper accumulation. Transmembrane flux was not measured for any of them.	mctB mutant showed 1,000-fold and 100-fold lower burden in lungs and lymph nodes of guinea pigs; the ctpV mutant reached wild-type levels in mice and guinea pigs, with reduced lung damage and longer survival.	Four partially redundant components, in addition to cytoplasmic buffering by the metallothionein MymT.	Four partially redundant components plus cytoplasmic buffering by MymT; deletion of ctpV does not reduce bacterial burden.

Wells, 2013 [29]	MmpS4/MmpL4 and MmpS5/MmpL5	mmpS4, mmpS5, mmpL4, mmpL5	Iron (Fe^{3+}-Mycobactin/Fe^{3+}-Carboxymycobactin)	Inner membrane, periplasmic space	Export of siderophores; essential for growth in low iron availability and virulence	Structure of the soluble region of MmpS4 (residues 52-140) resolved by NMR; specific interaction with the periplasmic loop of MmpL4. MmpS5 binds specifically to MmpL5, MmpS4 interacts with MmpL4 and MmpL5. Structurally similar to periplasmic adapter proteins.	ΔmmpS4/S5 mutants do not grow in low iron concentrations and are deficient even in iron-rich media. Severe defect in siderophore secretion and synthesis. Membrane association of MbtG suggests a link between siderophore synthesis and transport.	Transport (efflux) for MmpL4 and MmpL5, based on ^{14}C-labelled siderophores quantified in cells and culture filtrate; Accessory for MmpS4, MmpS5 and Rv0455c. Essential for siderophore secretion, with a strong virulence defect in mice.	mmpS4/mmpS5 double mutant showed a 10,000-fold lower bacterial burden in mouse lungs, and no infected mouse died within 180 days.	The MmpL4 and MmpL5 complexes are functionally redundant with each other, but the pair is not.	MmpL4 and MmpL5 are functionally redundant with each other, so both would have to be blocked; no inhibitor reported.
Ward, 2010 [93]	CtpV (copper exporting ATPase)	ctpV	Copper (Cu^{2+}, Cu^{+})	Plasma membrane	Active copper export, resistance to toxicity, and complete virulence	P-type ATPase with predicted copper and ATP binding domains, typical of these metal exporting ATPases. No detailed crystallographic structure.	Deletion of ctpV causes transcriptional response in 98 genes (high cellular stress). ΔctpV mutants survive close to the wild type, but with less lung damage, lower immune response, and higher host survival. First direct link between bacterial copper response and virulence in Mtb.	Transport inferred (efflux) for MctB (100-fold copper accumulation in the mutant) and for CtpV (csoR induction indicating higher intracellular copper); Accessory for CtpA and CtpB, whose disruption produced neither growth impairment nor copper accumulation. Transmembrane flux was not measured for any of them.	mctB mutant showed 1,000-fold and 100-fold lower burden in lungs and lymph nodes of guinea pigs; the ctpV mutant reached wild-type levels in mice and guinea pigs, with reduced lung damage and longer survival.	Four partially redundant components, in addition to cytoplasmic buffering by the metallothionein MymT.	Four partially redundant components plus cytoplasmic buffering by MymT; deletion of ctpV does not reduce bacterial burden.
Williamson, 2020 [49]	PE5–PPE4–EspG3 heterotrimer (ESX-3 type VII secretion substrates and their	pe5 (Rv0285), ppe4 (Rv0286), espG3 (Rv0289); EspG3 orthologue	Iron (Fe^{3+}) and Zinc (Zn^{2+}), indirectly through ESX-3	Cytosol (EspG3 chaperone); cell envelope (ESX-3 apparatus)	Chaperone-mediated recognition and targeting of PE–PPE substrates to ESX-3; no direct metal transport demonstrated	Crystal structure of the PE5mt–PPE4mt–EspG3mm heterotrimer from the ESX-3 system, solved in two crystal forms: $P2_12_12_1$ at 3.00 Å (PDB 6UUJ) and I422 at 3.30 Å (PDB 6VHR). EspG3 interacts exclusively	No transcriptomic or transport data. The structure provides a structural basis for the absence of cross-talk between ESX systems, showing how each EspG recognises its own subset of PE–PPE substrates.	Accessory. Essential for growth in a conditional mutant, with the defect reversed by iron, by zinc or by conditioned supernatant; TnSeq showed the system is required for both	Δesx-3 and ΔesxH highly attenuated in human macrophages; median survival of infected mice 65 days for	None identified. The system is shared by the siderophore and the heme routes.	Essentiality requires conditional approaches for validation, and the species translocated by the system is not

	cytosolic chaperone)	from M. marinum (MMAR_0548)				with PPE4, at the tip distal to the PE5 interaction, shielding the hydrophobic tip of PPE4 from solvent in a manner similar to EspG5. The C-terminal helical domain of EspG3 is dynamic, alternating between open and closed states. In contrast to the ESX-5 heterotrimers solved previously, the PE–PPE heterodimer engages its chaperone at a markedly different angle and presents different faces of the PPE protein to it.		siderophore- and heme-mediated iron utilization.	ΔesxH versus 22 days for the wild type, largely reversed by complementation.		defined; it is a secretion machine rather than a transporter.
Wolschendorf, 2011 [96]	MctB (Rv1698)	rv1698 (mctB in Mtb), ms3747 (in M. smegmatis)	Copper (Cu^{2+}, Cu^{+})	Outer membrane	Copper export, resistance to copper toxicity, and essentiality for virulence	Outer membrane channel essential for maintaining low intracellular copper levels. Mutants accumulate copper 100 times above normal, becoming highly sensitive.	ΔmctB mutant reduces bacterial load by 1,000-fold in the lungs and 100-fold in the lymph nodes of infected guinea pigs. In mice, persistence defect exacerbated by dietary copper supplementation, indicating a key role for copper in the host antimicrobial response.	Transport inferred (efflux) for MctB (100-fold copper accumulation in the mutant) and for CtpV (csoR induction indicating higher intracellular copper); Accessory for CtpA and CtpB, whose disruption produced neither growth impairment nor copper accumulation. Transmembrane flux was not measured for any of them.	mctB mutant showed 1,000-fold and 100-fold lower burden in lungs and lymph nodes of guinea pigs; the ctpV mutant reached wild-type levels in mice and guinea pigs, with reduced lung damage and longer survival.	Four partially redundant components, in addition to cytoplasmic buffering by the metallothionein MymT.	Four partially redundant components plus cytoplasmic buffering by MymT; deletion of ctpV does not reduce bacterial burden.
Zhang, 2020 [32]	ESX-3 system, MmpL5,	esx-3 (locus),	Iron (Fe^{3+}-Mycobactin/Carbo	Internal membrane (MmpL5, ESX-	Essentiality ESX-3 iron uptake via siderophore/heme;	No detailed structural information, predominant	TnSeq revealed opposing requirements for 165 genes in the presence of	Accessory. Essential for growth in a conditional mutant, with the defect	Δesx-3 and ΔesxH highly attenuated in	None identified. The system is	Essentiality requires conditional

	Rv2047c, Zur (transcriptional repressor)	mmpL5, rv2047c, zur	xymycoctin; Fe^{2+}-Heme)	3), cytoplasmic (Zur, Rv2047c)	MmpL5 siderophore secretion; Rv2047c mycobactin use; Zur regulates heme genes	functional/genetic characterization.	heme/hemoglobin versus siderophores. ESX-3 essential for siderophores and heme; MmpL5 main pump for siderophores; Rv2047c essential specifically for mycobactin; Zur required for efficient heme utilization.	reversed by iron, by zinc or by conditioned supernatant; TnSeq showed the system is required for both siderophore- and heme-mediated iron utilization.	human macrophages; median survival of infected mice 65 days for ΔesxH versus 22 days for the wild type, largely reversed by complementation.	shared by the siderophore and the heme routes.	approaches for validation, and the species translocated by the system is not defined; it is a secretion machine rather than a transporter.
Zhang, 2022 [33]	Rv0455c	rv0455c	Iron (Fe^{3+}-Mycobactin/Carboxymycobactin)	Periplasmic (soluble and membrane-anchored forms)	Essential for siderophore secretion; promotes efficient export to the extracellular environment	Crystallographic structure reveals new folding: helical bundle with 'cinch' by essential disulfide bridge. Both forms (soluble and anchored) are functional in siderophore secretion.	Δrv0455c mutants with almost complete blockage in the secretion of mycobactins/carboxymycobactins. Intracellular accumulation of siderophores generates cellular toxicity. Severely compromised replication in infected mice. Rv0455c is essential for the Mtb siderophore secretion system.	Transport (efflux) for MmpL4 and MmpL5, based on ^{14}C-labelled siderophores quantified in cells and culture filtrate; Accessory for MmpS4, MmpS5 and Rv0455c. Essential for siderophore secretion, with a strong virulence defect in mice.	mmpS4/mmpS5 double mutant showed a 10,000-fold lower bacterial burden in mouse lungs, and no infected mouse died within 180 days.	The MmpL4 and MmpL5 complexes are functionally redundant with each other, but the pair is not.	MmpL4 and MmpL5 are functionally redundant with each other, so both would have to be blocked; no inhibitor reported.

Note: The references presented in this table were described in the main manuscript.

Table S3. Preferred Reporting Items for Systematic reviews and Meta-Analyses extension for Scoping Reviews (PRISMA-ScR) Checklist [101].

SECTION	ITEM	PRISMA-ScR CHECKLIST ITEM	REPORTED ON PAGE #
**TITLE**			
Title	1	Identify the report as a scoping review.	1
**ABSTRACT**			
Structured summary	2	Provide a structured summary that includes (as applicable): background, objectives, eligibility criteria, sources of evidence, charting methods, results, and conclusions that relate to the review questions and objectives.	1
**INTRODUCTION**			
Rationale	3	Describe the rationale for the review in the context of what is already known. Explain why the review questions/objectives lend themselves to a scoping review approach.	2
Objectives	4	Provide an explicit statement of the questions and objectives being addressed with reference to their key elements (e.g., population or participants, concepts, and context) or other relevant key elements used to conceptualize the review questions and/or objectives.	3
**METHODS**			
Protocol and registration	5	Indicate whether a review protocol exists; state if and where it can be accessed (e.g., a Web address); and if available, provide registration information, including the registration number.	39
Eligibility criteria	6	Specify characteristics of the sources of evidence used as eligibility criteria (e.g., years considered, language, and publication status), and provide a rationale.	39 and SM
Information sources*	7	Describe all information sources in the search (e.g., databases with dates of coverage and contact with authors to identify additional sources), as well as the date the most recent search was executed.	39 and SM
Search	8	Present the full electronic search strategy for at least 1 database, including any limits used, such that it could be repeated.	39 and SM
Selection of sources of evidence†	9	State the process for selecting sources of evidence (i.e., screening and eligibility) included in the scoping review.	39 and SM
Data charting process‡	10	Describe the methods of charting data from the included sources of evidence (e.g., calibrated forms or forms that have been tested by the team before their use, and whether data charting was done independently or in duplicate) and any processes for obtaining and confirming data from investigators.	39 and SM
Data items	11	List and define all variables for which data were sought and any assumptions and simplifications made.	39 and SM, Table S4
Critical appraisal of individual sources of evidence§	12	If done, provide a rationale for conducting a critical appraisal of included sources of evidence; describe the methods used and how this information was used in any data synthesis (if appropriate).	Not applicable
Synthesis of results	13	Describe the methods of handling and summarizing the data that were charted.	39 and SM
**RESULTS**			
Selection of sources of evidence	14	Give numbers of sources of evidence screened, assessed for eligibility, and included in the review, with reasons for exclusions at each stage, ideally using a flow diagram.	3 and Figure 2
Characteristics of sources of evidence	15	For each source of evidence, present characteristics for which data were charted and provide the citations.	11, 17, 20 and 30 (Tables 1–4)
Critical appraisal within sources of evidence	16	If done, present data on critical appraisal of included sources of evidence (see item 12).	Not applicable
Results of individual sources of evidence	17	For each included source of evidence, present the relevant data that were charted that relate to the review questions and objectives.	4 to 28
Synthesis of results	18	Summarize and/or present the charting results as they relate to the review questions and objectives.	5, 16 and 29 (Figures 4–6)

**DISCUSSION**			
Summary of evidence	19	Summarize the main results (including an overview of concepts, themes, and types of evidence available), link to the review questions and objectives, and consider the relevance to key groups.	Page 34 and 35 (Table 5)
Limitations	20	Discuss the limitations of the scoping review process.	Table 5
Conclusions	21	Provide a general interpretation of the results with respect to the review questions and objectives, as well as potential implications and/or next steps.	39
**FUNDING**			
Funding	22	Describe sources of funding for the included sources of evidence, as well as sources of funding for the scoping review. Describe the role of the funders of the scoping review.	41

Note: JBI = Joanna Briggs Institute; PRISMA-ScR = Preferred Reporting Items for Systematic reviews and Meta-Ana'lyses extension for Scoping Reviews; SM = Supplementary Material.

Table S4. Databases, terms and operators used in the search strategy.

**Data Bases:**		
Pubmed	Web Of Science	Embase
**Query Terms:**		
#1 Species/Pathology: Tuberculosis[MH] OR “Mycobacterium tuberculosis”[MH] OR “Mycobacterium smegmatis”[MH] OR Tuberculosis[TIAB] OR “Mycobacterium tuberculosis”[TIAB] OR “Mycobacterium smegmatis”[TIAB] OR “Koch”[TIAB] #2 Membrane Transporter: “Membrane Transport Proteins”[MH] OR “Membrane Transport Proteins”[TIAB] OR “Transporter”[TIAB] OR “Pump”[TIAB] OR “Periplasmic protein”[TIAB] OR “Gated”[TIAB] OR “Channel”[TIAB] OR “ATPase”[TIAB] OR “Metal transport”[TIAB] OR “Ion transport”[TIAB] OR “Ion Channel”[TIAB] OR “ATP-binding cassette”[TIAB] OR “ABC transport”[TIAB] OR “ABC superfamily”[TIAB] OR “Major Facilitator Superfamily”[TIAB] OR “MFS superfamily”[TIAB] OR “MATE family”[TIAB] OR “SMR family”[TIAB] OR “RND superfamily”[TIAB] OR “Siderophore”[TIAB] #3 Heavy Metals: “Metals”[MH] OR “Metal”[TIAB] OR “Heavy Metal”[TIAB] OR “Iron”[TIAB] OR “Copper”[TIAB] OR “Manganese”[TIAB] OR “Zinc”[TIAB] OR “Nickel”[TIAB] OR “Cobalt”[TIAB] OR “Cadmium”[TIAB] OR “Cation”[TIAB] OR “Ion”[TIAB] OR “organometallic”[TIAB] OR “chelates”[TIAB] OR “Heme”[TIAB]	TS=(“Tuberculosis” OR “Mycobacterium tuberculosis” OR “Mycobacterium smegmatis” OR “koch”) AND TS=(“Membrane Transport Protein” OR “transport” OR “pump” OR “periplasmic protein” OR “gated” OR “channel” OR “ATPase” OR “metal transport” OR “ion transport” OR “ion channel” OR “ATP binding cassette” OR “ABC transport” OR “ABC superfamily” OR “Major Facilitator Superfamily” OR “MFS superfamily” OR “MATE family” OR “SMR family” OR “RND superfamily” OR “Siderophore”) AND TS=(“Metals” OR “metal” OR “Heavy Metal” OR “Iron” OR “Copper” OR “Manganese” OR “Zinc” OR “Nickel” OR “Cobalt” OR “Cadmium” OR “cation” OR “Ion” OR “organometallic” OR “chelates” OR “Heme”)	(‘tuberculosis’/exp OR ‘mycobacterium tuberculosis’/exp OR ‘mycobacterium smegmatis’/exp OR tuberculosis:ti,ab OR ‘mycobacterium tuberculosis’:ti,ab OR ‘mycobacterium smegmatis’:ti,ab OR koch:ti,ab) AND (‘membrane transport protein’ OR ‘membrane transport protein’:ti,ab OR transporter:ti,ab OR pump:ti,ab OR ‘periplasmic protein’:ti,ab OR gated:ti,ab OR channel:ti,ab OR atpase:ti,ab OR ‘metal transport’:ti,ab OR ‘ion transport’:ti,ab OR ‘ion channel’:ti,ab OR ‘atp binding cassette’:ti,ab OR ‘abc transport’:ti,ab OR ‘abc superfamily’:ti,ab OR ‘major facilitator superfamily’:ti,ab OR ‘mfs superfamily’:ti,ab OR ‘mate family’:ti,ab OR ‘smr family’:ti,ab OR ‘rnd superfamily’:ti,ab OR siderophore:ti,ab) AND (‘metal’/exp OR metal:ti,ab OR ‘heavy metal’:ti,ab OR iron:ti,ab OR copper:ti,ab OR manganese:ti,ab OR zinc:ti,ab OR nickel:ti,ab OR cobalt:ti,ab OR cadmium:ti,ab OR cation:ti,ab OR ion:ti,ab OR organometallic:ti,ab OR chelates:ti,ab OR heme:ti,ab)

Table S5. Excluded studies, after full-text reading, and justifications.

n	Authors	Article's Title	Year of Publication	Reason of Exclusion
1	Daniel D. Agranoff And Sanjeev Krishna	Metal ion homeostasis and intracellular parasitism	1998	Study design
2	Daniel Agranoff And Sanjeev Krishna	Metal ion transport and regulation in mycobacterium tuberculosis	2004	Study design
3	Qurat Ul Ain, Amandeep Singh, Iqubal Singh et al.	Synthesis, characterization and anti-tubercular activities of copper(ii) Complexes of substituted 2,3-isatin bisthiosemicarbazones: An experimental And theoretical approach	2023	Wrong population
4	José M. Argüello, Daniel Raimunda et al.	Mechanisms of copper homeostasis in bacteria	2013	Study design
5	Lina J. Bird, Maureen L. Coleman, Dianne K. Newmanb	Iron and copper act synergistically to delay anaerobic growth of Bacteria	2013	Wrong population
6	Martine Braibant, Philippe Gilot, Jean Content	The atp binding cassette (abc) transport systems of Mycobacterium tuberculosis	2000	Study design
7	Keith A. Brown And Colin Ratledge	Iron transport In mycobacterium smegmatis: Ferrimycobactin Reductase (nad(p)h:Ferrimycobactin Oxidoreductase), The Enzyme releasing iron from its carrier	1975	Wrong population
8	Hermes Reyes Caballeroa, Gregory C. Campanelloa, And David P. Giedroca,	Metalloregulatory proteins: Metal selectivity and allosteric Switching	2011	Study design
9	Mathieu F. M. Cellier	Nramp: From sequence to Structure and mechanism of Divalent metal import	2012	Study design
10	Debasmita Chatterjee, A. R. Day a Manasi , Sumit Kumar Rastogi et al.	Involvement of cora of mycobacterium smegmatis in Exerting intrinsic resistance towards structurally unrelat ed Antibiotics	2024	Wrong population
11	Liu Chen, Xiaohui Li, Piao Xu, et al.	A novel zinc exporter ctpg enhances resistance to zinc Toxicity and survival in mycobacterium bovi	2022	Wrong population
12	Nicholas Chim, Rodrigo Torres, Yuqi Liu, et al.	The structure and interactions of periplasmic Domains of crucial mmpl membrane proteins from Mycobacterium tuberculosis	2015	Wrong population
13	Ha Yeon Cho, Hyo Je Cho, Myung Hee Kim et al.	Blockage of the channel to heme by the e87 side chain in the gaf domain Of mycobacterium tuberculosis doss confers the unique sensitivity of doss to oxygen	2011	Wrong population

14	Seoung-Ryoung Choi, Barb Switzer, Bradley E. Britigan et al.	Gallium porphyrin and gallium nitrate synergistically inhibit Mycobacterial species by targeting different aspects of iron/heme Metabolism	2020	Wrong population
15	Moloko C. Cholo , Maborwa T. Matjokotja , Ayman G. Osman et al.	Role of the kdpde regulatory Operon of mycobacterium Tuberculosis in modulating bacterial Growth in vitro	2021	Wrong population
16	Tao Cui, Lei Zhang, Xizhou Wang et al.	Uncovering new signaling proteins and potential drug targets Through the interactome analysis of mycobacterium tuberculosis	2009	Wrong population
17	Bonnie J. Cuthbert, Jessica Mendoza, Rodger De Miranda et al.	The structure of mycobacterium thermoresistibile mmps5 Reveals a conserved disulfide bond across mycobacteria	2024	Wrong population
18	Jaiyanth Daniel, Liz Abraham, Amanda Martin et al.	Rv2477c is an antibiotic-sensitive manganese-dependent abc-f Atpase in mycobacterium tuberculosis	2018	Wrong population
19	P. Domenech, A.S. Pym, M. Cellier et al.	Inactivation of the mycobacterium tuberculosis nramp orthologue (mnth) does not a¡ect virulence in a mouse model of tuberculosis	2002	Wrong population
20	Rebecca K. Donegan, Yibo Fu, Jacqueline Copeland, et al.	Exogenously scavenged and endogenously synthesized heme Are differentially utilized by mycobacterium tuberculosis	2022	Wrong population
21	Carine Sao Emani And Norbert Reiling	The efflux pumps rv1877 and Rv0191 play differential roles in The protection of mycobacterium Tuberculosis against chemical Stress	2024	Wrong population
22	Peter M Eze, Viktor Simons, Tino Seidemann, et al.	Serratiochelins a and b from serratia marcescens show Xenosiderophoric characteristics towards acinetobacter Baumannii and mycobacterium tuberculosis	2021	Wrong population
23	Yue Fu, Feng-Ming James Chang And David P. Giedroc	copper transport and trafficking at the host–bacterial pathogen Interface	2014	Study design
24	Amit Gaur, Vijay Kumar Sharma, Sonal Shree et al.	Characterization of ecca3, a cbbx family atpase from the esx-3 secretion Pathway of m. Tuberculosis	2017	Wrong population
25	Kunal Mohan Gokhale, Aditya Manivannan Iyer	Deployment of iron uptake machineries As targets against drug resistant Strains of mycobacterium tuberculosis	2022	Study design

26	James M. Harrington, Heekwang Park, Yongcheng Ying et al.	Characterization of fe(iii) sequestration by an analog of the cytotoxic Siderophore brasilibactin a: Implications for the iron transport Mechanism in mycobacteriaw	2011	Wrong population
27	Ping He , Bing Zhao , Wencong He et al.	Impact of msmeg5257 deletion on mycolicibacterium Smegmatis growth	2024	Wrong population
28	Nadia Herrera, Grigory Maksaev, Elizabeth S. Haswell et al.	Elucidating a role for the Cytoplasmic domain in the Mycobacterium tuberculosis Mechanosensitive channel of large Conductance	2018	Wrong population
29	Victoria Hodgkinson And Michael J. Petris	Copper homeostasis at the Host-pathogen interface	2012	Study design
30	Tianyu Hu, Xiaolin Yang, Yuanchen Zhu et al.	Authors, some righmolecular basis for substrate transport of Reserved; exclusive Licensee american Mycobacterium tuberculosis abc importer dppabcd	2024	Wrong population
31	Irene Iscla, Gal Levin, Robin Wray et al.	Defining the physical gate of a mechanosensitive channel, mscl, by Engineering metal-binding sites	2004	Wrong population
32	Shunsuke Ishida, Masayoshi Arai, Hiroki Niikawa et al.	Inhibitory effect of cyclic trihydroxamate siderophore, desferrioxamine e, On the biofilm formation of mycobacterium species	2011	Wrong population
33	Christopher M. Jones And Michael Niederweis	Mycobacterium tuberculosis can utilize heme as an iron source	2011	Study design
34	Garima Khare, Vibha Gupta, Prachi Nangpal et al.	Ferritin structure from mycobacterium tuberculosis: Comparative study with homologues identifies Extended c-terminus involved in ferroxidase activity	2011	Wrong population
35	Garima Khare, Prachi Nangpal, Anil K. Tyagi	Differential roles of iron storage proteins In maintaining the iron homeostasis in Mycobacterium tuberculosis	2017	Wrong population
36	Chul Won Lee David P. Giedroc	1h, 13c, and 15n resonance assignments of nmtr, a ni(ii)/co(ii) Metalloregulatory protein of mycobacterium tuberculo	2012	Wrong population
37	Debora Lucarelli, Michael L. Vasil, Wolfram Meyer-Klaucke et al.	The metal-dependent regulators fura and furb from Mycobacterium tuberculosis	2008	Study design
38	Cressida A. Madigan, Tan-Yun Chenga, Emilie Layrea et al.	Lipidomic discovery of deoxysiderophores reveals A revised mycobactin biosynthesis pathway in Mycobacterium tuberculosis	2012	Wrong population

39	Cressida A. Madigan, Amanda Jezek Martinot, Jun-Rong Wei et al.	Lipidomic analysis links mycobactin Synthase k to iron uptake and virulence in M. Tuberculosis	2015	Wrong population
40	Rakesh Maharjan, Zhemin Zhang, Philip A. Klenotic et al.	Cryo-em structure of the mycobacterium smegmatis mmpl5- Acpm complex	2024	Study design
41	Sarah A. Marcus, Sarah W. Sidiropoulos, Howard Steinberg et al.	Csor is essential for maintaining copper Homeostasis in mycobacterium tuberculosis	2016	Wrong population
42	Berthold F. Matzanke, Rudolf Böhnke, Ute Möllmann et al.	Transport and utilization of rhizoferrin bound iron in mycobacterium Smegmatis	1999	Wrong population
43	Courtney F. Mcqueen, John T. Groves	Toxicity of the iron siderophore mycobactin j in mouse macrophages: Evidence for a hypoxia response	2021	Wrong population
44		Meeting report: World tuberculosis day symposium 2012	2013	Study design
45	By Annj . M . Messenger, Richardm Hallandcolin Ratledge	Iron uptake processes in mycobactevium vaccae r877r, a mycobacterium Lacking mycobactin	1986	Wrong population
46	Marvin J. Millera, Andrew J. Walza, Helen Zhua et al.	Design, synthesis and study of a mycobactin-artemisinin Conjugate that has selective and potent activity against Tuberculosis and malaria	2011	Wrong population
47	Mushtaq Ahmad Mir, Haryadi S. Rajeswari, Usha Veeraraghavan et al.	Molecular characterisation of abc transporter type ftse and ftsx Proteins of mycobacterium tuberculosis	2006	Wrong population
48	Abhishek Mishra	Proteomic analysis of hydroxyl radical-induced resuscitation Of hypoxia-induced dormant mycobacterial cells	2018	Wrong population
49	Abhinav Mohanty, Biswamaitree Subhadarshanee, Pallavi Barman et al.	Iron mineralizing bacterioferritin a from mycobacterium tuberculosis Exhibits unique catalase-dps-like dual activities	2018	Wrong population
50	Ryan R. Monfeli And Craig Beeson	Targeting iron acquisition by mycobacterium tuberculosis	2007	Study design
51	Olivier Neyrolles, Elisabeth Mintz And Patrice Catty	Zinc and copper toxicity in host defense against pathogens: Mycobacterium tuberculosis as a model example of an Emerging paradigm	2013	Study design
52	Oyebode Olakanmi, Bradley E. Britigan, And Larry S. Schlesinger	Gallium disrupts iron metabolism of mycobacteria residing Within human macrophage	2000	Wrong population
53	Oyebode Olakanmi, Banurekha Kesavalu, Maher Y. Abdalla et al.	Iron acquisition by mycobacterium tuberculosis residing within myeloid dendritic cells	2013	Wrong population

54	Tiara Padayachee, David C. Lamb, David R. Nelson et al.	Structure–function analysis of the essential mycobacterium Tuberculosis p450 drug target, cyp121a1	2024	Wrong population
55	Rahul Pal, Saif Hameed, Zeeshan Fatima	Altered drug efflux under iron deprivation unveils Abrogated mmpl3 driven mycolic acid transport And fluidity in mycobacteria	2018	Wrong population
56	Akankshika Parida, Abhinav Mohanty, Bharat T. Kansara et al.	Impact of phosphate on iron mineralization and mobilization in Nonheme bacterioferritin b from mycobacterium tuberculosis	2019	Wrong population
57	Akankshika Parida, Abhinav Mohanty, Rohit Kumar Raut et al.	Modification of 4-fold and b-pores in bacterioferritin from Mycobacterium tuberculosis reveals their role in fe2+ entry and Oxidoreductase activity	2022	Wrong population
58	Akankshika Parida, Gargee Bhattacharyya, And Rabindra K. Behera	Rational pore engineering reveals the relative Contribution of enzymatic sites and self-assembly Towards rapid ferroxidase activity and Mineralization: Impact of electrostatic guiding and Cage-confinement in bacterioferritin	2025	Wrong population
59	Colin Ratledge	Iron, mycobacteria and tuberculosis	2004	Study design
60	Geetha Vani Rayasam	Mmpl3 a potential new target For development of novel Anti-tuberculosis drugs	2013	Study design
61	G. Marcela Rodriguez, Richard Gardner Navneet Kaur Otto Phanstiel I	Utilization of fe3+-acinetoferrin analogs as an iron source By mycobacterium tuberculosis	2008	Wrong population
62	Elena G. Salina, Olga Ryabova, Anthony Vocat b, Boris Nikonenko et al.	New 1-hydroxy-2-thiopyridine derivatives active against both Replicating and dormant mycobacterium tuberculosis	2017	Study design
63	Paola Santosa, Fabian Lopez-Vallejoa, David Ramírezb et al.	Identification of mycobacterium tuberculosis ctpf as a target for designing New antituberculous compounds	2020	Wrong population
64	Paola Santos, Luz M. Salazar, Milena Maya-Hoyos et al.	Specific targeting to the mycobacterium tuberculosis P-type atpase membrane transporter, ctpf, of antituberculous Compounds obtained by structure-based des	2023	Wrong population
65	Gary J. Sharman, Dudley H. Williams, David F. Ewingt et al.	Isolation, purification and structure of exochelin ms, the extracellular Siderophore from mycobacterium smegmatis	1995	Wrong population
66	R. Sze Kely And S. T. Cole	Mechanistic insight into mycobacterial mmpl protein Function	2016	Study design

67	Yan-Ting Tao, Qing Huang, Ya-Li Jiang et al.	Up-regulation of slc39a2(zip2) mrna in peripheral blood Mononuclear cells from patients with pulmonary tuberculosi	2013	Wrong population
68	Abed Tarapdar, James K. S. Norris, Oliver Sampson et al.	The design and synthesis of an antibacterial Phenothiazine–siderophore conjugate	2018	Wrong population
69	Kristopher E. Van Zandt, Fatoumata B. Sow, William C. Florence et al.	The iron export protein ferroportin 1 is differentially expressed In mouse macrophage populations and is present in the Mycobacterial-containing phagosome	2008	Wrong population
70	Aleksey A. Vatlin , Egor A. Shitikov, Mohd Shahbaaz et al.	Transcriptomic profile of Mycobacterium smegmatis in Response to an Imidazo[,2-b][,,4,5]tetrazine Reveals its possible impact on iron Metabolism	2021	Wrong population
71	Olivia Vergnolle, Hua Xu, And John S. Blanchard1	Mechanism and regulation of mycobactin fatty acyl-amp Ligase fadd33	2013	Wrong population
72	James J. De Voss, Kerry Rutter, Benjamin G. Schroeder Hua Su et al.	The salicylate-derived mycobactin siderophores of Mycobacterium tuberculosis are essential for Growth in macrophages	1999	Wrong population
73	James J. De Voss, Kerry Rutter, Benjamin G. Schroeder et al.	Iron acquisition and metabolism by mycobacteria	1999	Study design
74	Dirk Wagner, JöRg Maser, Barry Lai et al.	Elemental analysis of mycobacterium avium-, mycobacterium Tuberculosis-, and mycobacterium smegmatis-containing Phagosomes indicates pathogen-induced microenvironments Within the host cell’s endosomal system1	2005	Wrong population
75	Dirk Wagner, JöRg Maser, Ivana Moric et al.	Changes of the phagosomal elemental Concentrations by mycobacterium tuberculosis Mramp	2005	Wrong population
76	Sunanda Margrett Williams, Anu V. Chandran, Mahalingam S. Vijayabaskar et al.	A histidine aspartate ionic lock gates the iron passage in Miniferritins from mycobacterium smegmati	2014	Wrong population
77	John T. Williams, Jacob J. Baker, Huiqing Zheng et al.	A genetic selection for mycobacterium smegmatis mutants Tolerant to killing by sodium citrate defines a combined role for Cation homeostasis and osmotic stress in cell death	2023	Wrong population
78	Dong Yang, Yanfang Zhang, Ibrahima Sory Sow et al.	Antimycobacterial activities of hydroxamic acids and their Iron(ii/iii), nickel(ii), copper(ii) and zinc(ii) complexes	2023	Wrong population

79	Veena C. Yeruva, Sridevi Duggirala, V. Lakshmi et al.	Identification and characterization of a major cell wall-associated Iron-regulated envelope protein (irep-28) in Mycobacterium tuberculosis	2006	Wrong population
80	Solomon A. Yimer, Alemayehu G. Birhanu, Shewit Kalayou et al.	Comparative proteomic analysis of Mycobacterium tuberculosis lineage 7 and lineage 4 strains reveals Differentially abundant proteins Linked to slow growth and virulence	2017	Wrong population
81	Jiwon Youm And Milton H. Saier Jr.	Comparative analyses of transport proteins encoded within the Genomes of mycobacterium tuberculosis and mycobacterium Leprae	2012	Study design
82	Chen Zhu, Yanfeng Shi, Jing Yu et al.	Cryo-em structures of a prokaryotic heme transporter Cyddc	2023	Study design
83	B R Byers, J E Arceneaux	Microbial iron transport: Iron acquisition by pathogenic microorganisms	1998	Study design
84	Santos S., Arbuckle A., Greco A. et al.	Determining the effect of kmtr mutants, e101q, h102q, and h111q on the metal binding affinities	2023	Study design
85	Higgins K., Labrie G., Surette V. et al.	Investigating metal binding to kmtr	2023	Study design
86	Argüello J.M.	The roles of heavy metal transport atpases in bacterial virulence	2014	Study design
87	Pandey R., Rodriguez G.M.	Role of iron storage in iron homeostasis, physiology and virulence of mycobacterium tuberculosis	2013	Study design
88	Qing Luo, Huaichuan Duan, Hailian Yan et al.	Specifically targeting mtb cell-wall and tmm transporter: The Impact Factor: 2.520 Development of mmpl3 inhibitors	2021	Study design
89	Celia Whitford Goulding, Cedric Owens, Nick Chim et al.	Further characterization of a unique mycobacterial heme uptake system	2012	Study design
90	Fiorella Meneghettia, Stefania Villaa, Arianna Gelaina et al.	Iron acquisition pathways as targets for antitubercular drugs	2016	Study design
91	Harmston C.A., Iniguez A., Mcmath L. et al.	Investigating heme uptake in mycobacteria	2010	Study design
92	Xiaolin Yang, Tianyu Hu, Jingxi Liang et al.	An oligopeptide permease, oppabcd, requires an iron–sulfur cluster domain for functionality	2023	Wrong population
93	Rodger De Miranda, Alex Chao, Paul Sieminski et al.	Characterization of periplasmic iron transport in mycobacterium tuberculosis	2022	Study design
94	Rodger De Miranda, Alex Chao,	Elucidating iron transport in the periplasm of mycobacterium tuberculosis	2023	Study design

	Thaís Klevorn et al.			
95	Gauri Shankar And Yusuf Akhter	Molecular insights into ferric-siderophore transport by the putative tonb-dependent Transporter in mycobacterium tuberculosis	2024	Wrong population
96	Abhinav Mohanty, Kankshika Parida, Biswamaitree Subhadarshanee et al.	Alteration of coaxial heme ligands reveals the role of heme in Bacterioferritin from mycobacterium tuberculosis	2021	Wrong population
97	Sudhasini Panda, Shah Faisal, Krishna Kumar et al.	Role of regulatory proteins involved in iron homeostasis In pulmonary tuberculosis patients and their household Contacts	2022	Wrong population
98	Christina Grønberg, Qiaoxia Hu, Dhani Ram Mahato et al.	Structure and ion--release mechanism of pib--4--type atpases	2021	Wrong population
99	Dong Yang, David P. Klebl, Bc Sheng Zeng et al.	Interplays between copper and mycobacterium Tuberculosis groel1	2020	Wrong population
100	Peicheng Du, Charles D. Sohaskey And Lanbo Shi	Transcriptional and physiological Changes during mycobacterium Tuberculosis reactivation from Non-replicating persistence	2016	Wrong population
101	Sharmila Talukdar, Radheshyam Modanwal, Gaurav Kumar Chaubey et al.	Mycobacterium tuberculosis exploits sirt2 to trap iron for its Intracellular survival	2024	Wrong population
102	Caroline Barisch, Vera Kalinina, Louise H. Lefrançois et al.	Localization of all four znt zinc transporters in dictyostelium and impact of Znta and b knockout on bacteria killi	2018	Wrong population
103	Berthold F. Matzanke, Rudolf Böhnke, Ute Möllmann et al.	Iron uptake and intracellular metal transfer in mycobacteria mediated By xenosiderophores	1997	Wrong population
104	Ann J. M. Messenger And Colin Ratledge	Iron transport in mycobacterium smegmatis: Uptake of iron From ferric citrate	1982	Wrong population
105	Colin Ratledge	Transport of iron by mycobactin in mycobacterium smegmatis	1971	Wrong population
106	Colin Ratledge And Maureen Ewing	The occurrence of carboxymycobactin, the siderophore of pathogenic mycobacteria, as a second extracellular siderophore in mycobacterium srnegrna t i s	1996	Wrong population
107	Sathyavelu K. Reddy, Madhavi Kamireddi, Kiran Dhanireddy et al.	Eukaryotic-like adenylyl cyclases in mycobacterium tuberculosis H37rv	2001	Wrong population
108	Stephenson And Colin Ratledge	Iron transport in mycobacterium smegmatis : Uptake of iron from ferriexochelin	1979	Wrong population
109	Elke Goethe, Kristin Laarmann, Janita Lührs et al.	Critical role of zur and smtb in zinc homeostasis of Mycobacterium smegmatis	2020	Wrong population

110	By Jovana Gobin And Marcus A. Horwitz	Exochelins of mycobacterium tuberculosis remove iron from Human iron-binding proteins and donate iron to Mycobactins in the m. Tuberculosis cell wall	1996	Wrong population
111	Colin Ratledge And Brian J. Marshall	Iron transport in mycobacterium smegmatis: The role of mycobactin	1972	Wrong concept
112	Lionel P. Macham, Maris C. Stephenson And Colin Ratledge	Iron transport in mycobacterium smegmatis: The isolation, purification and function of exochelin MS	1977	Wrong concept
113	Colin Ratledge, Pankaj V. Patel And Janice Mundy	Iron transport in mycobacterium smegmatis: The location of mycobactin by electron microscopy	1982	Wrong concept
114	Stephen J. Lane, Peter S. Marshall, Richard J. Upton And Colin Ratledge	Isolation and characterization of carboxymycobactins as the second extracellular siderophores in mycobacterium smegmatis	1998	Wrong concept
115	Suraj Dhungana, Colin Ratledge And Alvin L. Crumbliss	Iron chelation properties of an extracellular siderophore exochelin MS	2004	Wrong concept
116	Minkui Luo, Evgeny A. Fadeev And John T. Groves	Mycobactin-mediated iron acquisition within macrophages	2005	Wrong concept
117	Shan Sun, Yan Gao, Xiaolin Yang et al.	Structural insights as to how iron-loaded siderophores are imported into mycobacterium tuberculosis by IrtAB	2022	Duplicate report
